# Profiling the interactome of oligonucleotide drugs by proximity biotinylation

**DOI:** 10.1038/s41589-023-01530-z

**Published:** 2024-01-17

**Authors:** Alfred Hanswillemenke, Daniel Tobias Hofacker, Michèle Sorgenfrei, Carolin Fruhner, Mirita Franz-Wachtel, Dirk Schwarzer, Boris Maček, Thorsten Stafforst

**Affiliations:** 1 Interfaculty Institute of Biochemistry, University of Tübingen, Tübingen, Germany; 2 Interfaculty Institute of Cell Biology, University of Tübingen, Tübingen, Germany; 3 Gene and RNA Therapy Center (GRTC), Faculty of Medicine, University of Tübingen, Tübingen, Germany

**Keywords:** RNA, Target identification, Nucleic acids

## Abstract

Drug-ID is a novel method applying proximity biotinylation to identify drug–protein interactions inside living cells. The covalent conjugation of a drug with a biotin ligase enables targeted biotinylation and identification of the drug-bound proteome. We established Drug-ID for two small-molecule drugs, JQ1 and SAHA, and applied it for RNaseH-recruiting antisense oligonucleotides (ASOs). Drug-ID profiles the drug–protein interactome de novo under native conditions, directly inside living cells and at pharmacologically effective drug concentrations. It requires minimal amounts of cell material and might even become applicable in vivo. We studied the dose-dependent aggregation of ASOs and the effect of different wing chemistries (locked nucleic acid, 2′-methoxyethyl and 2′-Fluoro) and ASO lengths on the interactome. Finally, we demonstrate the detection of stress-induced, intracellular interactome changes (actinomycin D treatment) with an in situ variant of the approach, which uses a recombinant biotin ligase and does not require genetic manipulation of the target cell.

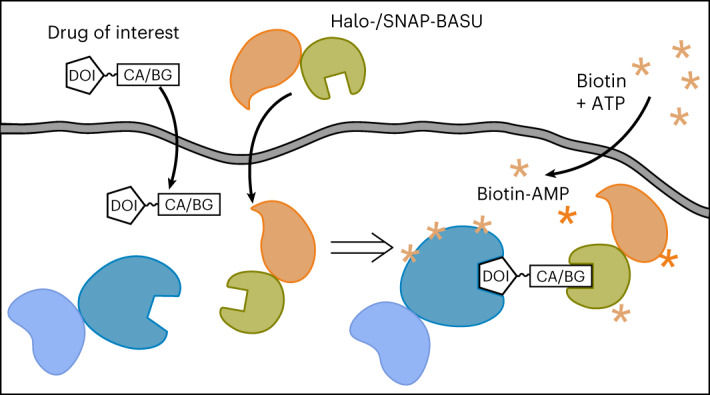

## Abstract (web summary)

Hanswillemenke, Hofacker and colleagues developed a proximity labeling-based method to identify protein interactome of small molecules and RNA drugs within living cells, facilitating the design and development of RNA drugs with enhanced pharmacological properties.

## Main

The interaction of drugs with proteins determines drug effects, including pharmacokinetics and pharmacodynamics, such as bioavailability, metabolism, efficacy and adverse effects^1^. Furthermore, drug–protein interactions play important roles in toxicity and immune response. To study small-molecule drug–protein interaction, various tools have been developed, including (photo)affinity-based and activity-based methods^2,3^, which enable the unbiased, drug-centered and comprehensive analysis of drug–protein interactomes. However, such methods require the design and synthesis of rather complex bi-functional and tri-functional capture probes, which comprise the drug, a warhead or photoreactive group and an enrichment tag^4,5^. Besides target identification, such probes found wide application in chemical genetics—for example, to profile the activity of numerous kinases with a single, unspecific kinase-reactive probe^6^. In contrast, the repertoire of methods to define the protein interactome of oligonucleotide (ON)-based drugs is more limited. Often applied is an in vitro assay in which an ON drug is immobilized on beads and incubated with cell lysate, followed by the elution of the protein binders with a competing ON drug^7^. This assay was successfully used to identify a large part of the currently approximately 80 known antisense oligonucleotide (ASO) binders and forms the basis of today’s knowledge on ON drug–protein interactions^8^ and how they modulate the pharmacological properties^9^ of ON drugs. Nevertheless, the assay is limited to in vitro application and, thus, cannot detect interactome changes induced by stress or interactomes specific to subcellular compartments. Furthermore, it is difficult to estimate how the in vitro applied ON drug concentrations refer to the therapeutically effective dose inside the cell. The impressive recent progress in the clinics^10,11^ has demonstrated the importance of a better understanding of ON drug–protein interactions. This includes a better understanding of the Janus-faced effects of various chemical modifications, such as phosphorothioate^8^, locked nucleic acid (LNA)^12^ and 2′-Fluoro (F)^13^, that, on the one hand, improve efficacy but, on the other hand, can cause toxicity, limiting their application in the clinic^1,8^. For example, a critical nucleotide position in RNaseH-recruiting ASOs has been identified recently as a hot spot for adverse ASO–protein interaction, leading to a clear improvement of therapeutic index^14^.

**Fig. 1:**
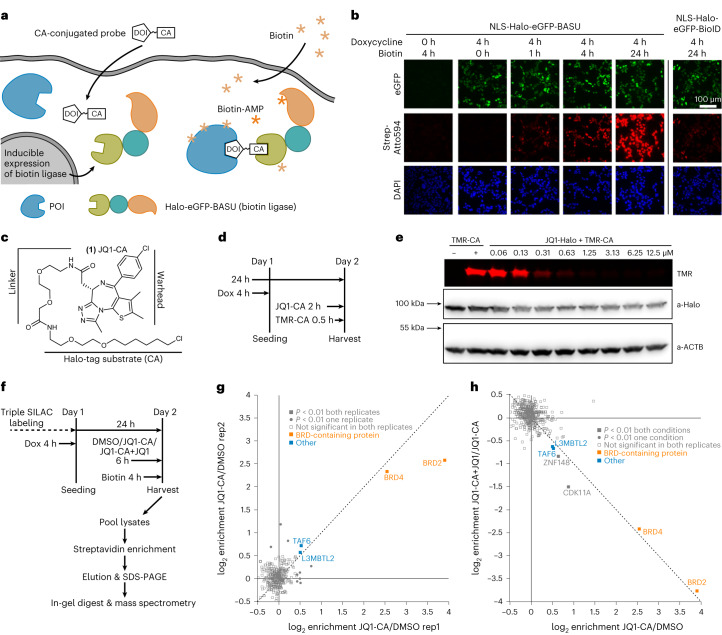
JQ1–protein interactions studied by Drug-ID. **a**, General scheme. After induced expression of a biotin ligase–Halo fusion, the Halo-tag covalently binds to a CA-modified probe. Addition of biotin will lead to the formation of biotin–AMP, which reacts with free lysine residues in close proximity to the probe. **b**, Expression and activity of the biotin ligase NLS-Halo-eGFP-BASU integrated into Flp-In T-REx 293 cells. Transgene expression was induced with doxycycline and followed by eGFP fluorescence (green channel). Cells were incubated with biotin (50 µM) for 0–24 h, and biotin deposition was stained with Atto594–streptavidin (red channel). Cell nuclei were stained with DAPI (blue channel) (*n* = 1). **c**, Structure of the JQ1-CA probe. **d**, Scheme of the passive uptake and conjugation assay of the JQ1-CA probe. **e**, PAGE analysis of JQ1-CA uptake into transgenic Flp-In T-REx 293 cells. Concentration-dependent conjugation of the JQ1-CA probe with the biotin ligase was visualized by conjugation of a competing fluorescent TMR-CA probe (red channel). Western blot against ACTB and Halo-tag served as loading controls (*n* = 1). **f**, Schematic overview of the SILAC–MS/MS experiment. **g**, SILAC–MS/MS for 500 nM JQ1-CA against DMSO. Plotted was the enrichment (log_2_) of replicate 1 against replicate 2 with swapped SILAC labeling. **h**, SILAC–MS/MS for the competition experiment 500 nM JQ1-CA against 500 nM JQ1-CA + 5 µM JQ1. The enrichment with JQ1-CA over DMSO is displayed on the *x* axis, and the depletion upon addition of JQ1 in 10-fold excess is displayed on the *y* axis (JQ1-CA + JQ1/JQ1-CA). Plotted was the enrichment of replicate 1 against replicate 2 with swapped SILAC labels. Statistical significance of hits in **g** and **h** was calculated with respect to the distance of the median of the distribution of all protein ratios as well as protein intensities using Perseus. Threshold for significance was set to *P* < 0.01 for all MS/MS experiments. Dox, doxycycline; rep, replicate. Source data

Here we present a novel method (Fig. 1a) that enables the drug-centered determination of the protein interactome of small-molecule and oligonucleotide-based drugs at pharmacologically relevant conditions directly in living cells.

## Results

### Drug-ID determines the protein interactome of JQ1 and SAHA

To establish Drug-ID, we determined the interactome of two well-known small molecules, JQ1 and SAHA. SAHA is a clinically applied histone deacetylase (HDAC) inhibitor. Its zinc-chelating hydroxamate moiety inhibits several HDACs from different classes of Zn^2+^-dependent amidohydrolases, including HDAC1, HDAC2 and HDAC3 (class I) as well as HDAC6 (class IIb)^15^. Most HDACs (for example, 1–3) form the catalytic part of larger multicomponent complexes, including the CoREST, NuRD, NCoR and Sin3 complex, which function in the epigenetic regulation of cell cycle and metabolism. Their inhibition is promising for the therapy of various indications^15^. JQ1 is an inhibitor selective for the bromodomain and extra-terminal (BET) family members, such as BRD4, of the larger class of bromodomain-containing proteins (BCPs), which recognize acetylated lysine residues, a common epigenetic mark^16^. Inhibition of BRD4 and further BET family members are highly relevant for cancer therapy. JQ1 binds to bromodomains in competition with acetylated lysine chains and, thereby, interferes with epigenetic regulation. For both small molecules, there are recent data on their respective protein–drug interactome obtained from activity-based^17^ and (photo)affinity-based^18,19^ approaches but also a very similar approach targeting Halo-SAHA^20^.

**Extended Data Fig. 1:**
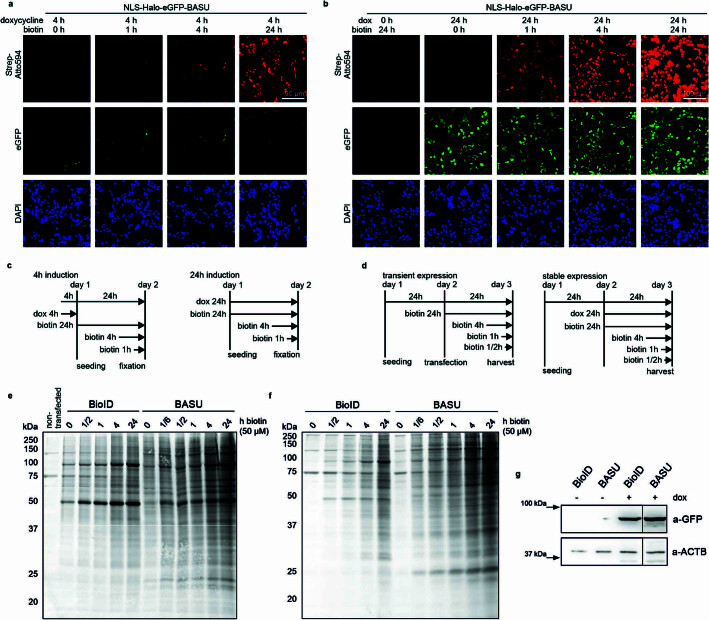
Characterization of HEK293T Flp-In T-REx NLS-Halo-eGFP-BASU and NLS-Halo-eGFP-BioID cell line. **a)** Expression and activity of the biotin ligase NLS-Halo-eGFP-BASU integrated into 293 Flp-In T-REx cells. Expression was induced with doxycycline for 4 h and followed by eGFP fluorescence (green channel). Cells were incubated with biotin (50 µM) for 0–24 h and biotin deposition was stained with Strep-Atto594 (red channel) after fixation and permeabilization of cells. Cell nuclei were stained with DAPI (blue channel). Acquisition and contrast settings are kept identical for panels a+b and Supplementary Fig. 1 for better comparison. **b)** Same as panel a but with doxycycline induction for 24 h (N=1 for panels a+b) **c)** Experimental setup for comparison of NLS-Halo-eGFP-BASU and NLS-Halo-eGFP-BioID in fluorescence microscopy (see panels a+b) **d)** Experimental setup for comparison of NLS-Halo-eGFP-BASU and NLS-Halo-eGFP-BioID in Western blot (see panels g+h). **e)** Western blot against biotinylated proteins in total cell lysates of HEK293T cells transiently expressing NLS-Halo-eGFP-BioID or NLS-Halo-eGFP-BASU. Cells were transfected with the respective construct 24 h before lysis and incubated with biotin (50 µM) for the indicated time. **f)** Western blot against biotinylated proteins in total cell lysates of HEK293T Flp-In T-REx cells stably expressing NLS-Halo-eGFP-BioID or NLS-Halo-eGFP-BASU. Transgene expression was induced for 24 h with 10 ng/ml doxycycline. **g)** Western blot against GFP and ACTB in HEK293T Flp-In T-Rex cell lines. Transgene expression was induced for 24 h with 10 ng/ml doxycycline. NLS-Halo-eGFP-BioID and NLS-Halo-eGFP-BASU show similar expression levels and expression is doxycycline dependent (N=1 for panels e-g).

First, we identified a convenient biotin ligase, such as BioID or BASU^21^. To achieve reasonable and homogenous expression, we created Flp-In T-REx 293 cell lines^22^ stably expressing either Halo-tagged^23^ eGFP-BASU^24^ or eGFP-BioID^25^. After weak transgene induction with doxycycline (4 h), we incubated cells with biotin for 1–24 h and analyzed biotin deposition (Fig. 1b, Extended Data Fig. 1 and Supplementary Fig. 1). We found Halo-BASU to give considerably more biotinylation than Halo-BioID and continued with that cell line. In a proof-of-principle experiment, we determined the protein interactome of JQ1. For that, we synthesized a CA-JQ1 (**1**) probe comprising JQ1 and 1-chloroalkane (CA)^26^. The latter is required for covalent attachment of JQ1 to the biotin ligase, mediated by the self-labeling activity of the Halo-tag (Fig. 1c). We tested the passive uptake of the probe and followed its covalent conjugation to Halo-eGFP-BASU inside the cell (Fig. 1d,e). Above a concentration of 500 nM, CA-JQ1 had reacted with the better part of the available biotin ligase (Fig. 1e).

The JQ1–protein interactome was identified in a stable isotope labeling by amino acids in cell culture (SILAC)–tandem mass spectrometry (MS/MS) experiment^27^ comparing mock-treated cells (DMSO), cells treated with CA-JQ1 (500 nM) and cells treated with CA-JQ1 (500 nM) in the presence of a 10-fold excess of competing JQ1 (5 µM). After transgene induction with doxycycline, cells (approximately 1.5 × 10^6^ in one six-well plate) were incubated with the respective drug and biotin according to Fig. 1f. After cell lysis, lysates were mixed, and biotinylated proteins were concentrated on streptavidin-coated magnetic beads, trypsinated in-gel and applied to high-resolution (HR) MS/MS analysis. The analysis gave a very clean picture (Fig. 1g). Out of the 325 different protein groups that were detected and quantified in both replicates, only very few proteins groups (4) were significantly enriched in both replicates (*P* < 0.01) (Fig. 1g). These comprised the well-known BET family members BRD2 and BRD4 with notable enrichment factors of up to 4 log_2_ units, but also TAF6, which was previously found^28^ in association with BRD4. Notably, all significantly enriched proteins got lost in the presence of a 10-fold excess of JQ1 (Fig. 1h), demonstrating the specific interactions of JQ1 responsible for the observed enrichments. TAF6 does not contain a bromodomain and is not expected to bind JQ1 directly. Its identification suggests that also interaction partners of direct binders of a probe can be biotinylated. This is rather typical for proximity profiling and in agreement with the estimated 10-nm labeling radius of activated biotin–AMP in living cells^29^.

**Fig. 2:**
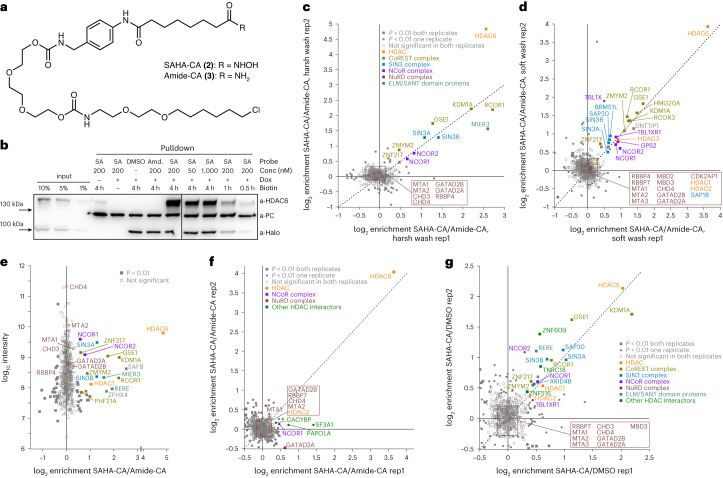
SAHA–protein interactions studied by Drug-ID. **a**, Structure of the SAHA-CA and the control probe Amide-CA. **b**, Biotinylation experiment with SAHA probes. Western blot after streptavidin pulldown (harsh wash) against HDAC6, Halo-eGFP-BASU and pyruvate carboxylase (PC). PC contains one biotin and served as loading/pulldown control. Halo-eGFP-BASU served as control for transgene. The analog western blot under soft wash conditions can be found in Supplementary Fig. 2b (*n* = 1). **c**, SILAC–MS/MS for 200 nM SAHA-CA probe against 200 nM Amide-CA probe under harsh wash conditions. Plotted was the enrichment of replicate 1 against replicate 2 with swapped SILAC labeling (*n* = 2). **d**, The same experiment as shown in **c** but under soft wash conditions. **e**, Data obtained from one replicate for the experiment shown in **c**, but raw MS intensity (log_10_ units) was plotted against enrichment. **f**, The same experiment as shown in **c** using Flp-In T-REx 293 NES-Halo-eGFP-BASU cells that localizes the biotin ligase to the cytoplasm. **g**, SILAC–MS/MS for 10 µM SAHA-CA probe against DMSO under harsh wash conditions. The biotin ligase was induced for 24 h, and biotinylation was carried out for 1 h in contrast to previous experiments. Plotted was the enrichment of replicate 1 against replicate 2 with swapped SILAC labels. Statistical significance of hits in **c–g** was calculated with respect to the distance of the median of the distribution of all protein ratios as well as protein intensities using Perseus. Threshold for significance was set to *P* < 0.01 for all MS/MS experiments. Conc, concentration; Dox, doxycycline; rep, replicate. Source data

**Extended Data Fig. 2:**
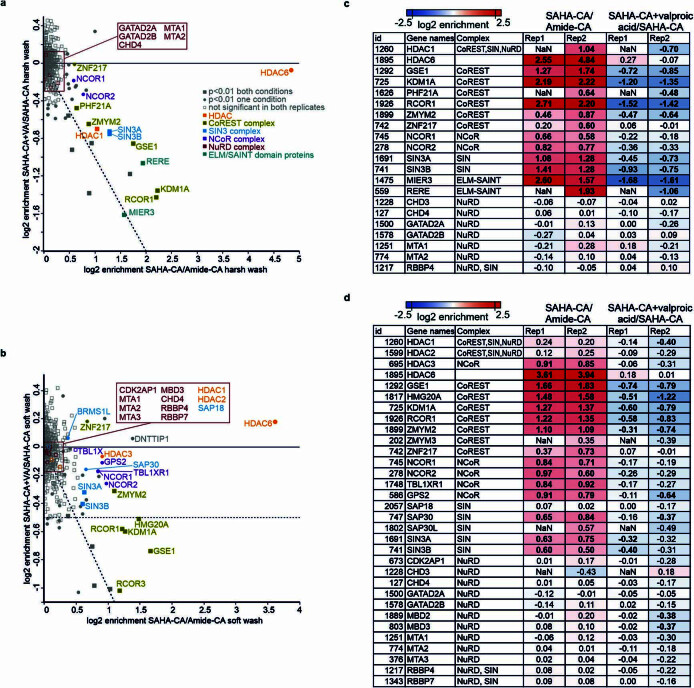
Enrichment of HDAC proteins and their complexes in HEK293T Flp-In T-REx cells with SAHA-CA probe. **a)** SILAC-MS/MS for the competition experiment 200 nM SAHA-CA against 200 nM SAHA-CA + 1 mM valproic acid. The enrichment with SAHA-CA over Amide-CA is displayed on the x-axis and the depletion upon addition of valproic acid is displayed on the y-axis (SAHA-CA+VA/ SAHA-CA). **b)** Same as panel a but under soft wash conditions. **c)** Enrichment of HDAC proteins and their previously established complex partners in SILAC-MS/MS experiment with 200 nM SAHA-CA under harsh wash conditions. Only protein groups quantified in at least one replicate are shown. Statistically significant changes (p < 0.01) in enrichment are highlighted in bold. Replicate 2 corresponds to the enrichment plot in panel a. **d)** Enrichment of HDAC proteins and their previously established complex partners in SILAC-MS/MS experiment with 200 nM SAHA-CA under soft wash conditions. Only protein groups quantified in at least one replicate are shown. Statistically significant changes (p < 0.01) in enrichment are highlighted in bold. Replicate 1 corresponds to the enrichment plot in panel b.

As a second example, we studied the SAHA–protein interactome. We relied on a recently characterized, cell-permeable SAHA-CA (**2**) probe^25^. As a control probe, we synthesized Amide-CA (**3**), containing a carboxamide instead of the hydroxamate moiety (Fig. 2a). Uptake, conjugation and western blot assays suggested that the SAHA-CA probe can be used in the range of 50 nM to 1 µM (Fig. 2b and Supplementary Fig. 2a). We performed SILAC–MS/MS experiments comparing SAHA-CA probe (200 nM) with Amide-CA probe (200 nM) with swapped SILAC labels. When the biotinylated proteins were washed under harsh conditions during pulldown, a very clean picture with 12 significantly enriched hits was obtained (Fig. 2c). Besides HDAC6, we found members of the CoREST, NCoR and SIN3 complexes. Interestingly, initially, we missed detecting and quantifying the class I HDACs (HDAC1–3) unambiguously, even though they are the core HDAC enzymes of the said complexes and expected to be direct binders of SAHA. Only in one replicate, we could detect a weak enrichment of HDAC1 (Fig. 2e). However, when we applied a soft wash^15^ after pulldown, we found members of all HDAC complexes, including HDAC6 and HDAC1–3 (Fig. 2d). Although HDAC3 was enriched, this was not the case for HDAC1 and HDAC2. Because HDAC1 and HDAC2 are members of the significantly enriched CoREST and SIN3A complexes, the SAHA-CA probe must have interacted with HDAC1/2 to initiate complex biotinylation. We speculate that HDAC1 and HDAC2 could have been sequestered by tight binding within their complexes, thus escaping from direct biotinylation. To test this hypothesis, we performed SILAC experiments under competition conditions. As competitor, we chose either valproic acid (VA, 1 mM), a class I-specific HDAC inhibitor, preferring HDAC1/2 over HDAC3, or Tacedinaline (CI-994, 2 µM) preferring HDAC3 over HDAC1/2 (ref. ^15^). Both competitors do not interact with HDAC6. Under competition with VA, we found HDAC6, HDAC3 and the NCoR complex rather unaffected compared to the CoREST complex (Extended Data Fig. 2). As expected, we found the inverse picture under Tacedinaline competition (Supplementary Fig. 3). Besides HDAC1/2, several members of the NuRD complex were detected but not enriched. However, some of the NuRD complex proteins—for example, CHD4—gave the highest intensities in the MS/MS experiment (Fig. 2e). We speculate that the large, unstructured CHD4 protein, which contains 176 lysine residues, was readily biotinylated independent of the probe, and, thus, the NuRD complex was not detectably enriched^30^. Furthermore, Drug-ID should be able to determine binding interactomes in subcellular compartments. To demonstrate this, we generated a transgenic 293 Flp-In T-REx cell line that expressed Halo-BASU with a nuclear export signal (NES), which forced the biotin ligase to the cytoplasm (Supplementary Fig. 4). As expected, NES-Halo-eGFP-BASU was only able to detect the interaction of SAHA with the cytosolic HDAC6 and missed all nuclear interaction partners (Fig. 2f).

The data demonstrate the general feasibility of Drug-ID to determine small-molecule–protein interactions with very little off-target hits. The results with JQ1 and SAHA largely overlap with those obtained from previous photoaffinity capture experiments^17–19^. However, in contrast to the latter, our method circumvents material UV irradiation and requires a relatively low number of cells. Furthermore, our method uses simpler probes and can be readily applied at physiologically relevant drug concentrations (for example, 200 nM). The ectopic expression of the biotin ligase might complicate its application but also opens the avenue to run the method tissue specific in vivo in the future^31^, which is hardly conceivable with photoaffinity-based or activity-based approaches. Still, we were not satisfied with missing out the known SAHA binders HDAC1/2. We speculated that we may find more hits if we could increase the amount of drug–biotin ligase inside the living cell. However, the cloud of hundreds of non-enriched proteins suggested that new hits might be covered in the background when simply more biotin ligase is expressed. Thus, we reduced the biotinylation time (from 4 h down to 1 h) such that a similar level of background biotinylation was obtained when the expression level of the biotin ligase was strongly increased (24-h versus 4-h doxycycline induction) (Extended Data Fig. 1a,b and Supplementary Fig. 5). At this expression level, SAHA-CA probe concentrations up to 10 µM should be possible until saturation of the Halo-tag is achieved (Supplementary Fig. 2a). We repeated the Drug-ID protocol with 10 µM SAHA-CA probe under harsh wash conditions and were able to find substantially more significantly enriched hits, including the direct interaction partners HDAC1/2, but also further components of the SIN3 and NCoR complexes (SAP30, ARID4B and TBL1XR1) and further HDAC interactors, such as ZNF609, ZNF516 and TNRC18 (Fig. 2g). Some of our findings—for example, the identification of GSE1—overlap with recent findings from a study that also established proximity biotinylation for small-molecule drugs^20^.

### Drug-ID identifies the protein interactome of gapmer ASOs

**Fig. 3:**
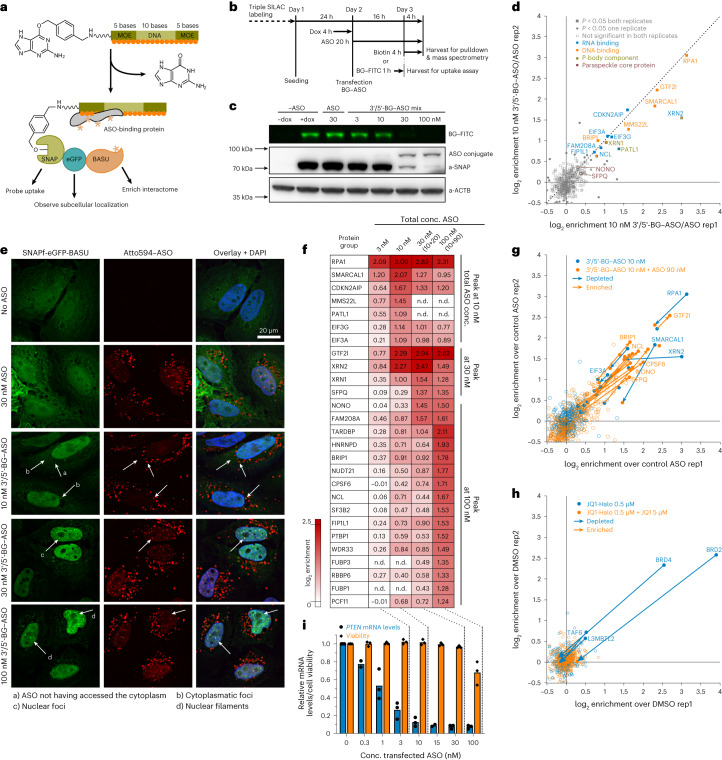
ASO–protein interactions studied by Drug-ID. **a**, Drug-ID was applied to an RNaseH-recruiting gapmer. For covalent conjugation to SNAP-eGFP-BASU, a benzylguanine moiety was attached. **b**, Schematic overview of uptake/conjugation and SILAC–MS/MS experiments. **c**, PAGE analysis of BG–ASO uptake into transgenic HeLa cells. Conjugation of the BG–ASO was visualized by conjugation of a competing fluorescent BG–FITC probe. Western blot served as loading control (*n* = 1). **d**, SILAC–MS/MS for 10 nM BG–ASO against 10 nM control ASO. Plotted was the enrichment (log_2_) of replicate 1 against replicate 2 with swapped SILAC labeling. Statistical significance of hits was calculated with respect to the distance of the median of the distribution of all protein ratios as well as protein intensities using Perseus (significance threshold *P* < 0.05). **e**, Fluorescence microscopy of ASO and biotin ligase. BG–ASO was transfected into transgenic HeLa cells expressing SNAP-eGFP-BASU (green channel). For visualization, 2 nM Atto594-labeled ASO (red channel) was spiked into transfections. Nuclei were stained with DAPI (blue channel). White arrows indicate structures with co-localization of Atto594–ASO and biotin ligase (*n* = 1). **f**, Enrichment profile (log_2_, against control ASO) for selected protein groups in four settings (3–100 nM ASO transfection). Shown are mean values from two replicates with alternated SILAC labeling. **g**, Competition experiment with the ASO drug. Overlaid plots for 10 nM BG–ASO against 10 nM control ASO in two replicates (blue), with the plot for the competition experiment 10 nM BG–ASO + 90 nM control ASO against 100 nM control ASO in two replicates (orange). **h**, Competition experiment for JQ1. Overlaid were the plot for JQ1 against DMSO in two replicates (blue) with the plot for the competition experiment JQ1-CA + 10-fold excess JQ1 against DMSO in two replicates (orange). **i**, Cell viability and knockdown efficacy of the BG–ASO. Shown are mean values and individual data points from independent biological replicates (*n* = 2–3). Dox, doxycycline; conc., concentration; rep, replicate; n.d., not determined. Source data

We wondered whether Drug-ID could profile the protein interactome of ON drugs at physiologically relevant drug concentrations inside living cells, an endeavor that has not been realized before. As a drug candidate, we chose an RNaseH gapmer against *PTEN* mRNA. It comprised a 10-nucleotide (nt) phosphorothioate DNA stretch flanked by 5-nt methoxyethyl (MOE) wings at both ends and resembles the design of clinically applied ASOs (Fig. 3a)^10^. To effect the properties of the ASO as little as possible, we attached an *O*^6^-benzylguanine (BG) moiety^32^ to the ASO to enable covalent in situ conjugation with the biotin ligase SNAP-eGFP-BASU^33,34^. We used a 1:1 mixture of 5′-terminal and 3′-terminal BG-modified ASO to avoid blocking one terminus for interaction with proteins completely^12^ and, thus, to capture the interactome more comprehensively. Analog to the small-molecule drugs, we characterized the formation of the covalent ASO–biotin transferase conjugate by transfecting varying amounts of the BG-modified ASO into a transgenic HeLa cell line expressing SNAP-eGFP-BASU under control of doxycycline (Fig. 3b,c and Supplementary Fig. 6). When 10 nM ASO was transfected, approximately 30% of the biotin ligase was conjugated to it (Fig. 3c).

**Extended Data Fig. 3:**
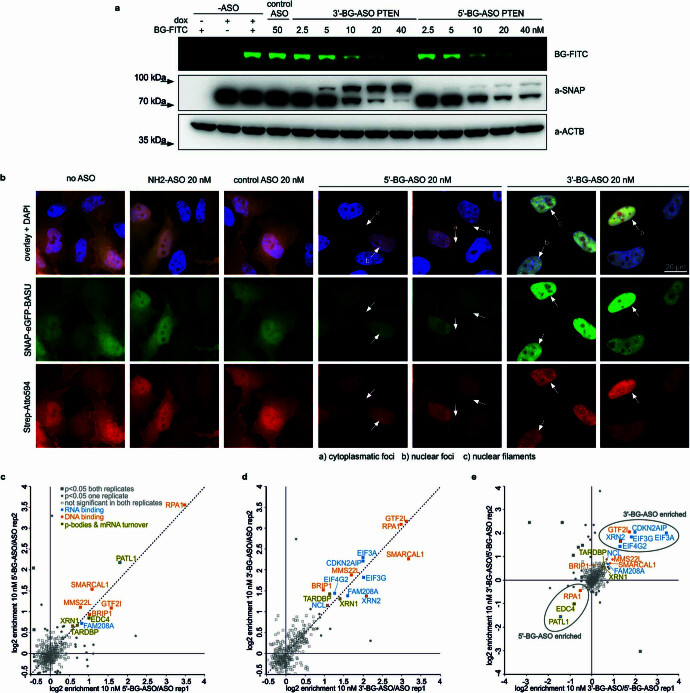
Drug-ID. Comparison of 3′-BG- and 5′-BG-ASO (20mer MOE) in uptake, localization and protein interactome. **a)** Uptake of 3′-BG-ASO and 5′-BG-ASO into transgenic HeLa cells. Concentration-dependent conjugation of the respective BG-ASO was visualized by progressive loss of conjugation of a competing fluorescent BG-FITC probe via denaturing SDS-PAGE-analysis (green channel). Western blot against ACTB and SNAP-tag served as loading controls. Conjugation of the biotin ligase with the ASO led to a clear shift (ASO-conjugate). Saturation of the biotin ligase was observed at the same concentration for 3´-BG- and 5´-BG-ASO (N = 1). **b)** Biotin deposition followed the localization of the ASO-SNAP-eGFP-BASU conjugate. Transgene expression (eGFP signal, green channel), biotinylation (Strep-Atto594 signal, red channel), fixation and DAPI staining (blue channel) were performed as in Extended Data Fig. 1a. Cells were transfected with either 20 nM unmodified ASO (no BG), 20 nM NH2-ASO, with 20 nM 3′-BG-ASO, or with 20 nM 5′-BG-ASO. Only BG-ASOs led to a localization of the eGFP and biotin signal to the nucleus. The 5′-BG-ASO-conjugate also localized to cytosolic foci (arrow a) while the 3′-BG-ASO-conjugate exhibited a stronger accumulation in nuclear foci (arrow b) (N=1). **c)** SILAC-MS/MS for 10 nM 5′-BG-ASO against control ASO (lacking BG). Plotted was the enrichment (log2) of replicate 1 against replicate 2 with swapped SILAC labels. All proteins significantly (p < 0.05) enriched in both replicates are highlighted and colorized according to their molecular function (blue = RNA binding, orange = DNA binding, gold = P-body components). **d)** Same as panel c) but for 10 nM 3′-BG-ASO **e)** SILAC-MS/MS for 10 nM 3′-BG-ASO against 10 nM 5′-BG-ASO. Plotted was the enrichment (log2) of replicate 1 against replicate 2 with swapped SILAC labels. All proteins enriched with 3′-BG-ASO or 5′-BG-ASO (see panels c+d) are highlighted and colorized to their molecular function (blue = RNA binding, orange = DNA binding, gold = P-body components). Some proteins were preferentially enriched by one of the ASOs. The 3´-BG-ASO preferred several nuclear proteins and proteins that require a free 5´-terminus, like GTF2I and the nuclear 5′>3′ exoribonuclease XRN2. In contrast, the 5´-BG-ASO preferred cytosolic p-body components, like EDC4 and PATL1. Intriguingly, these binding preferences match with the localization observed in microscopy (panel b).

**Extended Data Fig. 4:**
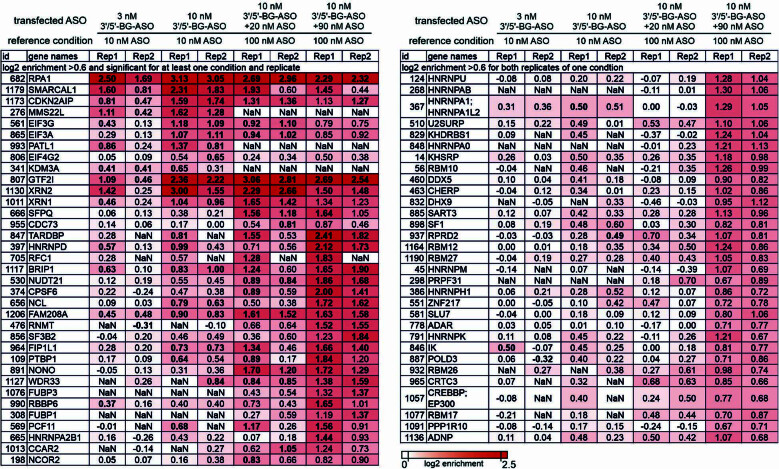
Drug-ID. Dose-dependency of the ASO-protein interactome. Extended table of enrichment profiles (log2, against control ASO lacking BG) in all four settings, differing in the amount of applied total ASO (3–100 nM). For 30 nM and 100 nM total ASO, the amount of BG-ASO (3´+5´mix) was kept constant at 10 nM and filled up to the total amount with control ASO lacking BG. Shown are the values from two replicates with swapped SILAC labeling. Statistically significant changes (p < 0.05) are highlighted in bold. Left table: protein groups significantly enriched in at least one replicate (p < 0.05) with a log2 enrichment of >0.6. Right table: protein groups enriched in both replicates with a log2 enrichment of >0.6. The corresponding Scatterplots are displayed in Supplementary Fig. 7.

To identify the protein binders, we performed a SILAC–MS/MS experiment in replicate with label swap (Fig. 3b,d). An ASO lacking the BG moiety served as control. In the initial experiment, we transfected 10 nM ASO, an amount sub-stoichiometric regarding the biotin ligase (Fig. 3c). We identified more than a dozen interaction partners with an enrichment of up to 3.5 log_2_ units (Fig. 3d, Extended Data Fig. 4b,d and Supplementary Fig. 7). All significantly enriched hits belonged to the group of DNA or RNA binding proteins, several of which have been described extensively previously^7,8^, such as RPA1 (ref. ^35^) and NCL. Others have been described only very recently^36^, such as PATL1 and XRN1, or, to our knowledge, not at all, such as XRN2 and GTF2I. We also detected various proteins that have been reported as ASO binding proteins before, but we did not find them significantly enriched. Notably, these include the two core paraspeckle proteins p54nrb (NONO) and PSF (SFPQ)^8^, which can interact with ASOs to form nuclear foci (paraspeckle-like) and nuclear filaments^37^. However, when we used fluorescence microscopy to follow localization of the ASO–biotin ligase conjugate inside the cell (eGFP channel; Fig. 3e), we observed the formation of small cytosolic foci and larger nuclear foci in a strongly ASO dose-dependent manner. The process was independent of covalent conjugation of ASO and SNAPf-eGFP-BASU. Accordingly, the induction of foci was also found when we kept the amount of BG-modified ASO constant at 10 nM and complemented the total ASO amount to 100 nM by filling up with the terminally unmodified, conjugation-incompetent control ASO (Supplementary Fig. 8). Furthermore, we spiked in small amounts of Atto594-labeled ASO to show that the unconjugated ASO resided in the foci, too (Fig. 3e and Supplementary Fig. 8). At the highest ASO concentration (100 nM), we observed the formation of nuclear filaments containing ASO–biotin ligase conjugates and non-conjugated ASO. To better understand this aggregation phenomenon, we determined the protein interactome at increasing ASO concentration between 3 nM and 100 nM in four steps. Specifically, we treated the cells either with 3 nM BG–ASO or 10 nM BG–ASO or with 10 nM BG–ASO + 20 nM competing control ASO (total 30 nM ASO) or with 10 nM BG–ASO + 90 nM competing control ASO (total 100 nM ASO). As discussed above, we used a 1:1 mixture of 5′-BG and 3′-BG modified ASO as the two individual ASOs enriched slightly different protein groups (Extended Data Fig. 3), which is in accordance with literature^12^. We found clear differences among the four conditions (Fig. 3f, Extended Data Fig. 4 and Supplementary Fig. 7). Some proteins, for example RPA1, PATL1 and XRN1/2, were clearly enriched at low ASO dose (3 nM and 10 nM) and are (partly) outcompeted when adding the conjugation-incompetent control ASO (for example, 10 nM BG–ASO + 90 nM ASO). However, a larger number of proteins, including the paraspeckle core proteins NONO and SFPQ, are initially not enriched but appear very prominently at higher ASO doses. Clearly, ASO drugs behave very differently from small-molecule drugs (Fig. 3g,h). The latter are applied in high concentration (for example, 200 nM; Fig. 3g) and ideally bind to few, lowly expressed target proteins very specifically. Accordingly, a small-molecule drug is readily outcompeted (for example, the JQ1 probe by a 10-fold excess JQ1; Fig. 3h). In contrast, an oligonucleotide drug is applied at low concentrations (for example, 10 nM in media; the actual and effective intracellular concentration inside the cell is difficult to estimate) but binds to dozens of highly expressed unspecific proteins. How these manifold interactions and aggregation phenomena—as demonstrated by the transfection of a competing ASO—modulate the pharmacological properties of an ASO drug is a matter of debate^8,38^. It appears plausible that the changes that we observed in the ASO protein interactome might directly correlate with the formation of cytosolic foci, nuclear foci and nuclear filaments—for example, with increasing ASO dose as observed by microscopy. In fluorescence microscopy (Fig. 3e and Supplementary Fig. 8), we found the formation of cytosolic foci already at 10 nM ASO, whereas the formation of nuclear foci started at ASO concentration ≥30 nM. In accordance with that, we found P-body components—for example, XRN1 and PATL1—enriched in the MS analysis of the 10 nM ASO sample, before we found paraspeckle components enriched—for example NONO and SFPQ—which required ≥30 nM ASO concentration. The observations fit to data from the literature, which describe the localization of ASOs to P-bodies at low ASO concentration and the localization of NONO/SFPQ to paraspeckle-like nuclear foci and nuclear filaments with increasing ASO amounts^8^. With Drug-ID, we can correlate the MS-based interactome analysis not only with fluorescence microscopy but also with the pharmacological activity of the ASO drug at that specific, applied dose. Specifically, we tested the knockdown activity of the *PTEN* ASO by RT–qPCR (Fig. 3i and Supplementary Figs. 9 and 10) and determined a half maximal effective concentration (EC_50_) of 0.9 nM. Above an ASO concentration of 10 nM, there was virtually no further gain in knockdown, suggesting that the observed ASO aggregation phenomena are likely not important for effective knockdown. In contrast, many of the identified late-onset hits that appear side by side with nuclear foci and filaments might cause ASO-induced toxicity, which would be, at least for paraspeckle proteins, in agreement with the literature^8,13^. Indeed, using an ATP cell viability assay, we observed incipient loss of cell viability at 100 nM (Fig. 3i).

### Drug-ID identifies wing chemistry–dependent binding profiles

**Fig. 4:**
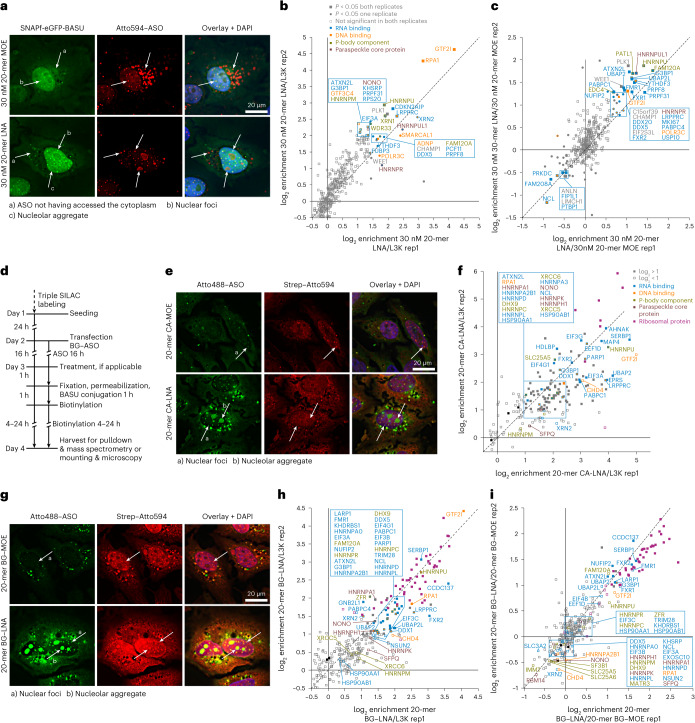
isASO-ID overcomes intracellular localization limitations of Drug-ID. **a**, Microscopy as in Fig. 3e. LNA-modified and MOE-modified ASO (red channel) were transfected at a final concentration of 30 nM (10 nM BG–ASO and 20 nM control ASO) into transgenic HeLa cells expressing SNAP-eGFP-BASU (green channel). White arrows indicate co-localization of Atto594–ASO and the biotin ligase (*n* = 1). **b**, SILAC–MS/MS for 10 nM BG–ASO and 20 nM control ASO for the LNA-modified ASO against Lipofectamine 3000. Plotted was the enrichment (log_2_) of replicate 1 against replicate 2 with swapped SILAC labeling (*n* = 2). **c**, SILAC–MS/MS for 10 nM BG–ASO and 20 nM control ASO for the LNA-modified ASO over the MOE-modified ASO. Plotted was the enrichment (log_2_) of replicate 1 against replicate 2 with swapped SILAC labeling. Statistical significance of hits in **b** and **c** was calculated with respect to the distance of the median of the distribution of all protein ratios as well as protein intensities using Perseus. Significance was set to *P* < 0.05 in **b** and **c**. **d**, Schematic overview of isASO-ID experiments. **e**, Fluorescence microscopy using Halo-BASU-His for biotinylation. Either 25 nM MOE-modified or LNA-modified Halo-ASO (20-mer) was transfected, followed by conjugation of Halo-BASU-His. To visualize ASO localization, 2 nM Atto488-labeled ASO (green channel) was spiked into transfections of BG–ASO. Biotin deposition was stained with Atto594–streptavidin (red channel). Cell nuclei were stained with DAPI (blue channel). White arrows indicate the appearance of structures with co-localization of Atto488–ASO and the biotin signal (*n* = 2). **f**, SILAC–MS/MS with 25 nM CA-ASO (20-mer LNA) over Lipofectamine 3000. Plotted was the enrichment (log_2_) of replicate 1 against replicate 2 with swapped SILAC labeling. **g**, Fluorescence microscopy using SNAP-BASU-His for biotinylation. Microscopy was carried out as described in **e**, transfecting either 25 nM MOE-modified or LNA-modified BG–ASO (20-mer) (*n* = 2). **h**, SILAC–MS/MS for 25 nM BG–ASO (20-mer LNA) over Lipofectamine 3000. **i**, SILAC–MS/MS experiment for comparison of enrichment after transfection with either 30 nM MOE-modified ASO or LNA-modified ASO. In **h** and **i**, the normalized enrichment (log_2_) of replicate 1 against replicate 2 with swapped SILAC labeling was plotted. rep, replicate.

**Extended Data Fig. 5:**
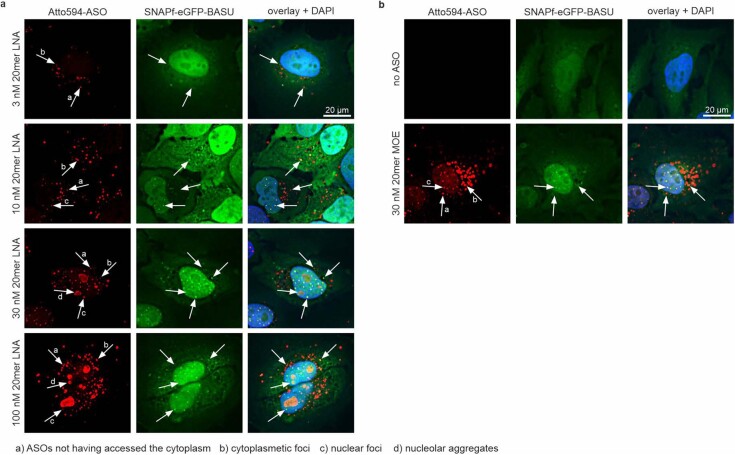
Drug-ID. Localization of the ASO-SNAP-eGFP-BASU conjugate depends on ASO dose and ASO chemistry. Determining localization of ASO and biotin ligase via fluorescence microscopy. Analog to Supplementary Fig. 5, increasing amounts of BG-ASO (Gapmers containing MOE and LNA wings) were transfected with Lipofectamine 3000 into transgenic HeLa cells expressing SNAP-eGFP-BASU (green channel). To visualize ASO localization, 2 nM Atto594-labeled ASO (red channel) were spiked into transfections of BG-ASO. Nuclei were stained with DAPI (blue channel). As in Supplementary Fig. 5 the concentration of BG-ASO was kept at 10 nM and control ASO (lacking BG) was co-transfected to increase total ASO amount. ASOs not having accessed the nuclei (arrow a), cytoplasmic foci (arrow b), nuclear foci (arrow c), and nucleolar aggregates (arrow d) are indicated with white arrows in all panels. **a)** In contrast to the 20mer MOE-ASO, the 20mer containing LNA wings accumulated already at 10 nM in nuclear foci and shifts to the nucleoli at the highest concentration (100 nM). b**)** The 20mer MOE ASO is shown for comparison at a concentration of 30 nM, further data see Supplementary Fig. 5. Transfection with Lipofectamine 3000 served as negative control.

**Extended Data Fig. 6:**
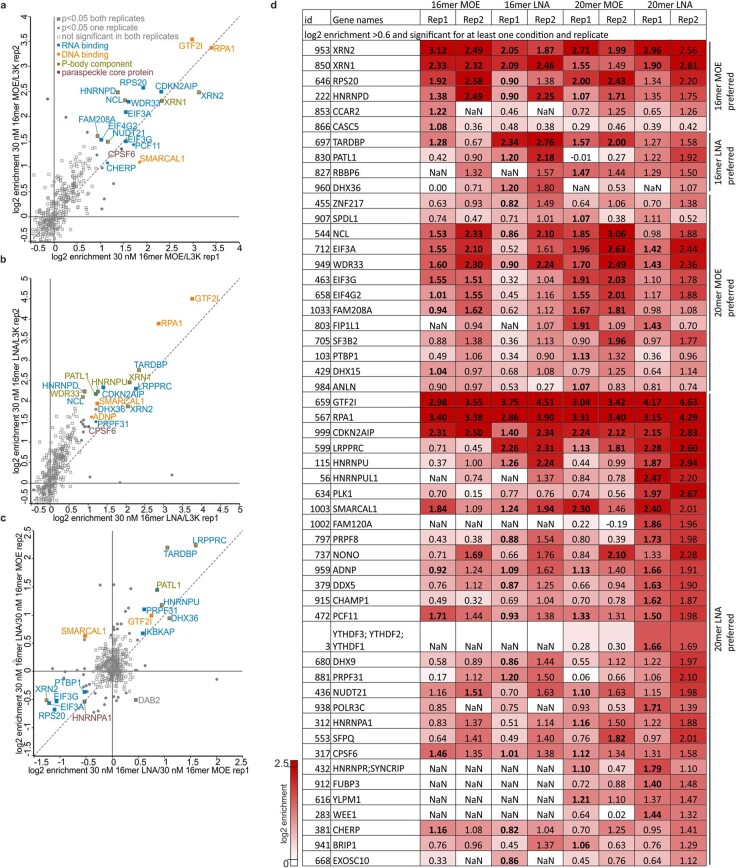
Drug-ID. Wing chemistry and ASO length determines ASO-protein interactome. **a)** - **c)** SILAC-MS/MS for BG-ASO (16mer or 20mer MOE or LNA modified ASOs) against Lipofectamine 3000 at 30 nM. Plotted was the enrichment (log2) of replicate 1 against replicate 2 with swapped SILAC labels. All proteins significantly (p < 0.05) enriched in both replicates are highlighted and colored according to their RNA- and/or DNA-binding function (blue = RNA binding, orange = DNA binding). Furthermore, selected paraspeckle core proteins (brown) and P-body components (gold) are highlighted. **a)** Enrichment with 10 nM BG-ASO + 20 nM NH_2_-ASO (16mer MOE) over Lipofectamine 3000. **b)** Enrichment with 10 nM BG-ASO + 20 nM NH_2_-ASO (16mer LNA) over Lipofectamine 3000. **c)** Enrichment with 10 nM BG-ASO + 20 nM NH_2_-ASO (16mer LNA) over 10 nM BG-ASO + 20 nM NH_2_-ASO (16mer MOE). **d)** Extended table of enrichment profiles (log2, against Lipofectamine 3000) in all four settings. 30 nM total ASO was transfected (10 nM BG-modified ASO and 20 nM NH_2_-modified ASO). Shown are the values from two replicates with swapped SILAC labeling. Statistically significant changes (p < 0.05) are highlighted in bold. Protein groups significantly enriched in at least one replicate (p < 0.05) with a log2 enrichment of >0.6 in at least one replicate and condition are shown.

**Extended Data Fig. 7:**
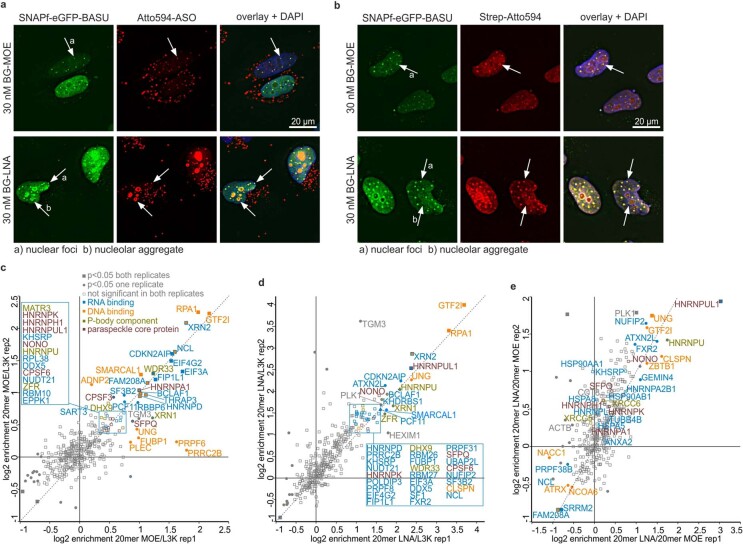
Drug-ID. NLS-SNAP-eGFP-BASU allows better biotinylation in nucleoli. Determining localization of ASO and biotin ligase via fluorescence microscopy. Analog to Extended Data Fig. 5, 30 nM of ASO containing either MOE or LNA wings were transfected with Lipofectamine 3000 into transgenic HeLa cells expressing SNAP-eGFP-BASU (green channel). To visualize ASO localization, 2 nM Atto594-labeled ASO (red channel) were spiked into transfections of BG-ASO. Nuclei were stained with DAPI (blue channel). As in Extended Data Fig. 5, 10 nM BG-ASO were transfected and 20 nM control ASO were spiked in. **a)** Colocalization of BG-modified ASOs and NLS-SNAP-eGFP-BASU. In case of transfection with LNA-modified ASO, NLS-SNAP-eGFP-BASU is capable of entering nucleoli in contrast to the enzyme without nuclear localization sequence (Extended Data Fig. 5) (N=1). **b)** Biotin is deposited in nucleoli using NLS-SNAP-eGFP-BASU (N=1). **c)-e)** SILAC-MS/MS for BG-ASO (20mer containing either MOE or LNA wings) at 30 nM against Lipofectamine 3000. Plotted was the enrichment (log2) of replicate 1 against replicate 2 with swapped SILAC labels. All proteins significantly (p < 0.05) enriched in both replicates are highlighted and colored according to their RNA- and/or DNA-binding function (blue = RNA binding, orange = DNA binding). Furthermore, selected paraspeckle core proteins (brown) and P-body components (gold) are highlighted. **c)** Enrichment with 10 nM BG-ASO + 20 nM NH_2_-ASO (20mer MOE) over Lipofectamine 3000. **d)** Enrichment with 10 nM BG-ASO + 20 nM NH_2_-ASO (20mer LNA) over Lipofectamine 3000. **e)** Enrichment with 10 nM BG-ASO + 20 nM NH2-ASO (20mer LNA) over 10 nM BG-ASO + 20 nM NH_2_-ASO (20mer MOE).

The chemical modification pattern of a specific ASO sequence is known to alter its target engagement, potency and toxicity. For RNaseH gapmers, the introduction of three to five 2′–4′ bridged nucleotides, such as LNA^39^ or constrained ethyl (cET)^40^, replacing the MOE wings in the flanking region of an ASO can improve the potency up to 10-fold^41^. Unfortunately, the inclusion of bridged nucleotides typically makes ASOs more sticky to proteins, which can lead to toxicity^42^. To see whether we can detect differential interactomes inside living cells, we repeated the Drug-ID protocol with the same ASO replacing all 10 terminal 2′-MOE with LNA nucleotides, at a total ASO concentration of 30 nM (10 nM BG–ASO + 20 nM NH_2_–ASO) (Fig. 4a–c and Extended Data Fig. 5). Indeed, we found a large number of known ASO-binding proteins significantly enriched, some of which are shared, some unshared, with the analog MOE ASO (Fig. 4b and Extended Data Fig. 6d,e). When we plotted the proteins by enrichment (MOE versus LNA ASO; Fig. 4c), we detected a small number of MOE-preferred hits (for example, NCL) but a rather large number of LNA-preferred ones, in accordance with literature^8,12^. We repeated the experiments also with 16-mer MOE and LNA ASOs (with three modified bases at each terminus) and got similar results, with the shorter ASOs tending to bind less strongly to the same interactome as the respective 20-mer analog (Extended Data Fig. 6). Still, we could define specific preferred binders for each chemistry and ASO length (Extended Data Fig. 6e). The microscopic analysis revealed that the (20-mer) LNA ASO—other than the MOE ASO—was strongly accumulated in the nucleoli; however, the biotinylation signal did not follow the ASO readily into this compartment, which suggests that some nucleolar binders might get missed out (Fig. 4a, Extended Data Fig. 5 and Supplementary Fig. 11). We equipped the SNAP-BASU enzyme with a nuclear localization signal (NLS), which made the ligase follow the ASO, resulting in a biotin deposition that better co-localized with the LNA ASO in the nucleoli (Extended Data Fig. 7a,b). However, the protein interaction patterns of both, the MOE and the LNA ASO, did not change notably (Extended Data Fig. 7c–e and Supplementary Fig. 12).

### In situ ASO-ID identifies more ASO–protein interactions

Although Drug-ID enabled the identification of known ASO binders with high confidence, the overall number of hits was rather limited with respect to the approximately 80 known ASO binders identified today^8^. The main reason for this seems to be the high background biotinylation, which is a general problem of proximity biotinylation tools^43^. As a consequence, many known ASO binders were detected but not (significantly) enriched due to their high level of background biotinylation already in the absence of an ASO, which may also be fostered by the lysine-rich nature and high intracellular concentration of many RNA and DNA binding proteins. The restriction of the biotinylation enzyme to a specific intracellular compartment is an additional factor to miss out relevant hits. To overcome such limitations, we developed another novel protocol for ASO drugs that we dub in situ ASO-ID (isASO-ID) where, in contrast to the Drug-ID protocol, the biotin ligase was not expressed inside the cells. Instead, wild-type cells were simply transfected with the ASO as before (Fig. 4d), and, after transfection was effective, cells were fixated with formaldehyde, permeabilized with a mild triton wash and then rinsed with a SNAP-BASU-His solution (prepared from *Escherichia coli*) to covalently trap the biotin ligase to the fixated ASOs inside the permeabilized cells. After washing the excess ligase away, cells were treated with biotin/ATP and harvested. Crosslinking was reversed by boiling the samples for 1 h, followed by streptavidin pulldown, gel purification, trypsin digest and MS analysis. Alternatively, the localization of the ASO and the deposition of biotin was followed by fluorescence microscopy (Fig. 4d).

**Extended Data Fig. 8:**
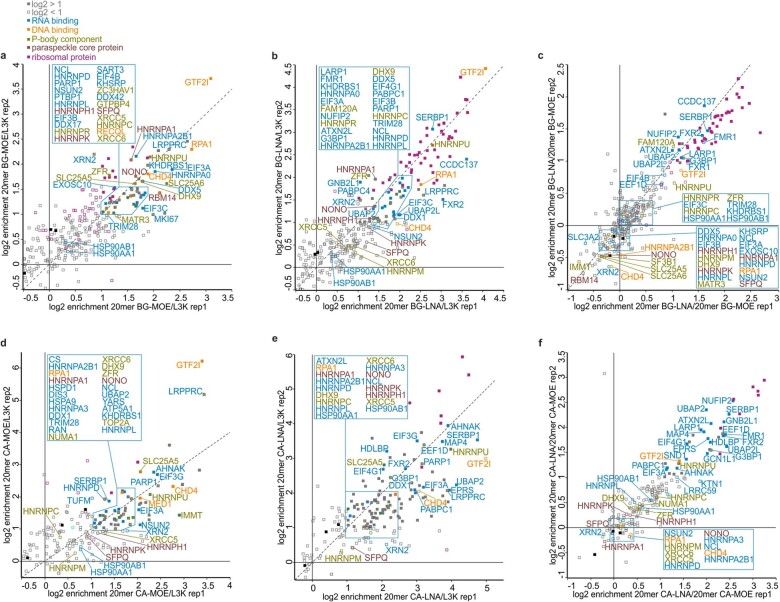
isASO-ID. Wing chemistry determines the ASO-protein interactome. SILAC-MS/MS for either BG-ASO or CA-ASO (20mer containing either MOE or LNA wings) at 25 nM against Lipofectamine 3000. Plotted was the normalized enrichment (log2) of replicate 1 against replicate 2 with swapped SILAC labels. In a), b), d) and e) all proteins enriched >2-fold in both replicates are highlighted and colored according to their RNA- and/or DNA-binding function (blue = RNA-binding, orange = DNA binding). Furthermore, selected paraspeckle core proteins (brown), P-body components (gold), and ribosomal proteins (purple) are highlighted as well as selected known ASO interactors not passing the threshold. In c) and f) the same proteins annotated in a), b), d) and e) are highlighted regardless their enrichment in this particular part of the experiment. **a)** Enrichment with 25 nM BG-ASO (20mer MOE) over Lipofectamine 3000 **b)** Enrichment with 25 nM BG-ASO (20mer LNA) over Lipofectamine 3000. **c)** Enrichment with 25 nM BG-LNA (20mer) over 25 nM BG-MOE (20mer). **d)** Enrichment with 25 nM CA-MOE (20mer) over Lipofectamine 3000. **e)** Enrichment with 25 nM CA-LNA (20mer) over Lipofectamine 3000. **f)** Enrichment with 25 nM CA-LNA (20mer) over 25 nM CA-MOE (20mer). For the isASO-ID SILAC-MS/MS experiments in panel a-f, detected proteins were colored according to their biological function as P-body component (gold), paraspeckle protein (red) and the annotated GO terms DNA binding (orange) and RNA binding (blue). Ribosomal proteins were colored in purple. Proteins passing the applied enrichment threshold (>1 log2 unit) are represented as filled squares. Carboxylases are highlighted as filled black squares and represent unregulated control proteins.

**Extended Data Fig. 9:**
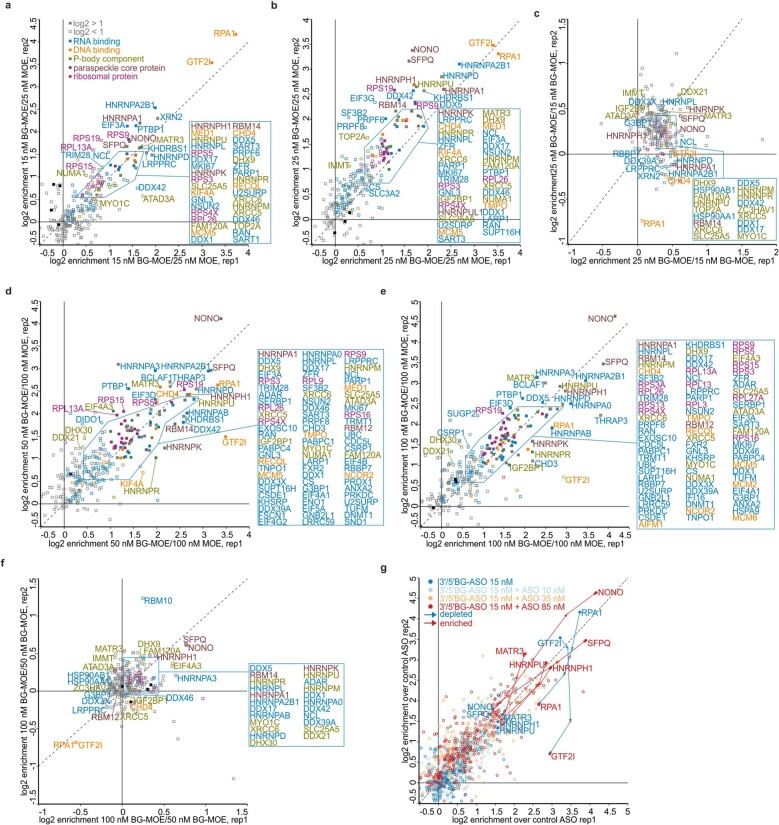
isASO-ID. Dose-dependent changes in the ASO-protein interactome. **a)-f)** SILAC-MS/MS for BG-ASO (20mer MOE) at increasing concentrations (15 nM, 25 nM, 50 nM, and 100 nM) against the control ASO. The amount of BG-ASO was constant for all transfections at 15 nM and conjugation-incompetent control ASO (20mer MOE) was spiked in to obtain the final ASO concentration to promote equal amounts of bound SNAP-BASU-His in all conditions. Plotted was the normalized enrichment (log2) of replicate 1 against replicate 2 with swapped SILAC labels. **a)** 15 nM BG-ASO over 25 nM control ASO. **b)** 15 nM BG-ASO and 10 nM control ASO over 25 nM control ASO. **c)** 15 nM BG-ASO and 10 nM control ASO over 15 nM BG-ASO. **d)** 15 nM BG-ASO and 35 nM control ASO over 100 nM control ASO. **e)** 15 nM BG-ASO and 85 nM control ASO over 100 nM control ASO. **f)** 15 nM BG-ASO and 85 nM control ASO over 15 nM BG-ASO and 35 nM control ASO. **g)** Four stacked plots. 15 nM BG-ASO over 25 nM control ASO (dark blue), 15 nM BG-ASO and 10 nM control ASO over 25 nM control ASO (light blue), 15 nM BG-ASO and 35 nM control ASO over 100 nM control ASO (orange), and 15 nM BG-ASO and 85 nM control ASO over 100 nM control ASO (red). Changes between the enrichments are indicated with arrows for panels g and h (blue for depletion upon increased ASO dose, red for further enrichment upon increased ASO dose). Highlighted are known ASO interactors and proteins with notable changes. For the isASO-ID SILAC-MS/MS experiments in panel a-f, detected proteins were colored according to their biological function as P-body component (gold), paraspeckle protein (red) and the annotated GO terms DNA binding (orange) and RNA binding (blue). Ribosomal proteins were colored in purple. Proteins in panels a-f passing the applied enrichment threshold (>1 log2 unit) are represented as filled squares. Carboxylases are highlighted as filled black squares and represent unregulated control proteins.

In the first isASO-ID experiment, we fused the biotin ligase BASU either to the SNAP-tag or to the Halo-tag and compared their performance with the 20-mer MOE and the 20-mer LNA ASO (at 25 nM ASO concentration), which carried the respective self-labeling moiety (BG or CA), by means of fluorescence microscopy and triple SILAC–MS/MS. According to fluorescence microscopy (Fig. 4e,g), the total biotinylation signal with the in situ protocol was much lower compared to the Drug-ID protocol. Accordingly, the acquisition time was increased approximately 10-fold, and the fluorescence signal upon Strep-594 staining was typically so low that the background of mitochondrial carboxylases was visible (Fig. 4e,g and Extended Data Fig. 8). Overall, SNAP-BASU-His gave more biotinylation signal than Halo-BASU-His, and the former gave a more intense staining of the nuclear and nucleolar structures (Fig. 4g). Triple SILAC–MS/MS experiments (Fig. 4f,h and Extended Data Fig. 9) revealed clear differences to the corresponding Drug-ID experiments (Fig. 3d and Extended Data Fig. 6). In accordance with the strongly reduced background (Supplementary Fig. 13), the total number of identified and quantified proteins was smaller, and no massive cloud of unregulated proteins was detected. Given the lack of a cloud in the origin of the scatter plots, we were unable to calculate statistical significance and to assign *P* values to the enriched proteins. We rather defined an arbitrary enrichment factor, typically >1 log_2_ unit, to call a protein a high-confidence hit. We used the mitochondrial carboxylases (always represented as filled black squares in isASO-ID plots) to estimate the quality of normalization in the scatter plots. By this, we assigned a large number of high-confidence hits with log_2_ enrichment factors between 1 and 5 (represented by filled squares), applied a Gene Ontology (GO) term analysis and colored hits accordingly (Fig. 4f,h,i). High-confidence hits belong to the group of RNA or DNA binding, P-body components, paraspeckle core proteins and ribosomal proteins. They now include many proteins that are known ASO binders but were not detected or not significantly enriched with Drug-ID before, such as XRCC5/6 and HSP90AA1/B1 (Extended Data Fig. 8). With a few exceptions (for example, NCL), the 20-mer LNA ASO typically gave higher enrichment factors than the analog MOE ASO (Fig. 4i and Extended Data Fig. 8), in accordance with the literature and the Drug-ID results. Halo-BASU-His and SNAP-BASU-His shared many hits; however, some differences were also observed. As in fluorescence microscopy (Fig. 4e versus Fig. 4g), SNAP-BASU-His showed stronger biotin deposition in nuclear and nucleolar structures when the 20-mer LNA ASO was applied (Fig. 4g). Accordingly, SNAP-BASU-His detected many more ribosomal proteins in the MS analysis (Fig. 4h). On the other hand, Halo-BASU-His was able to detect cytosolic proteins like chaperones as HSP90, HSPA8 and CCT8 with higher enrichment (Fig. 4f). Moreover, we monitored the interactome of a 20-mer ASO carrying 2′-F wings by fluorescence microscopy and SILAC–MS/MS (Supplementary Fig. 14) and compared it to the analog 2′-MOE ASO. Upon interaction with 2′-fluorinated ASOs, paraspeckle proteins can be degraded, a mechanism that substantially contributes to ASO toxicity^13^. Both ASOs largely shared the same protein binders; however, we observed that the 2′-F ASO binds them with higher enrichment at low ASO concentrations (for example, 15 nM). This fits well with data from literature, obtained with Nano-BRET^44,45^ and pulldown assays^12,13^, which also showed that 2′-F ASOs tend to be more sticky than 2′-MOE ASOs for the same proteins.

**Fig. 5:**
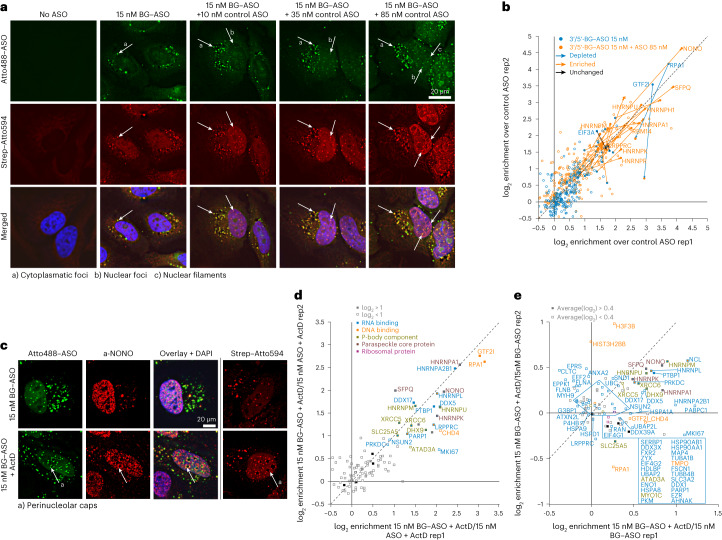
isASO-ID identifies the ASO interactome more comprehensively. **a**, Determining localization of ASO and biotinylated proteins via fluorescence microscopy. Increasing amounts of BG–ASO (15, 25, 50 and 100 nM; 20-mer MOE) were transfected (with Lipofectamine 3000) into wild-type HeLa cells. Microscopy was carried out as for Fig. 4e (*n* = 2). **b**, Exemplary competition experiment with the ASO drug. Overlaid was the enrichment plot for 15 nM BG–ASO against 25 nM control ASO in two replicates (blue), with the enrichment plot for the competition experiment 15 nM BG–ASO + 85 nM control ASO against 100 nM control ASO in two replicates (orange). **c**, Immunofluorescence microscopy for 15 nM BG–ASO (20-mer MOE) and the influence of ActD treatment (1.5 µg ml^−1^ for 1 h) on ASO localization and its co-localization with NONO. On the right, the influence of ActD treatment on biotin deposition is shown under the same conditions (*n* = 2). **d**, SILAC–MS/MS experiment for 15 nM BG–ASO (20-mer MOE) treated with ActD plotted against 15 nM control ASO treated with ActD. Plotted was the normalized enrichment (log_2_) of replicate 1 against replicate 2 with swapped SILAC labeling. **e**, SILAC–MS/MS experiment showing the effect of ActD treatment for the 15 nM BG–ASO (20-mer MOE) against the untreated control. Plotted was the normalized enrichment (log_2_) of replicate 1 against replicate 2 with swapped SILAC labeling. For the isASO-ID SILAC–MS/MS experiments in **d** and **e**, detected proteins were colored according to their biological function as P-body component (gold), paraspeckle protein (red) and the annotated GO terms DNA binding (orange) and RNA binding (blue). Ribosomal proteins are colored in purple. Proteins passing the applied enrichment threshold (>1 log_2_ unit for **d** and >0.4 in average for **e**) are represented as filled squares. Carboxylases are highlighted as filled black squares and represent non-enriched control proteins, which are pulled down due to their natural covalent modification with biotin. rep, replicate.

Because biotinoyl-5′-adenylate has a labeling range at the nanometer scale, not all hits can be assigned as direct binders, but the isASO-ID protocol may allow the illumination of changes in the ASO proximity with high sensitivity due to the largely reduced background. With Drug-ID, we detected a concentration-dependent change of ASO localization and aggregation, which resulted in a change in the detected interactome. We were wondering what this experiment would give with isASO-ID. For this, we repeated the concentration row with the 20-mer MOE ASO (15 nM to 100 nM), and, again, we kept the concentration of conjugation-competent BG–MOE ASO constant at 15 nM and filled up with control MOE ASO lacking the BG self-labeling moiety. Fluorescence microscopy showed, again, the formation of cytoplasmic foci at ≥10 nM ASO concentration, of nuclear foci at ≥25 nM as well as the formation of nuclear filaments at 100 nM (Fig. 5a). Among the most highly enriched hits in the MS analysis was, again, RPA1, and, again, this hit lost enrichment when we increased the dose of competing ASO (Fig. 5b and Extended Data Fig. 9). In contrast to Drug-ID (Fig. 3f, Extended Data Fig. 4 and Supplementary Fig. 7), we now quantified NONO/SFPQ already at a lower ASO concentration (15 nM), but, again, their enrichment factors increased steadily when increasing the ASO dose (Fig. 5b and Extended Data Fig. 9g), supporting the view that many of the detected ASO protein interactions are caused by ASO-induced aggregation and phase separation phenomena. Compared to Drug-ID, the isASO-ID protocol detected notably more known ASO binders with high enrichment—for example, XRCC5/6 and HSP90—likely due to the strongly reduced background biotinylation (Supplementary Fig. 13).

### isASO-ID identifies interactome changes induced by stress

Finally, we wanted to test if isASO-ID can track changes in the ASO–protein interactome that are induced by stress. Notably, the isASO-ID protocol freezes a cellular state by formaldehyde fixation, which is a relatively fast process (for example, compared to the biotinylation reaction in Drug-ID) and may allow to track changes down to a minute timescale. We chose actinomycin D (ActD) treatment, which leads to global block of translation, to induce stress. It was shown previously that perinucleolar caps are formed in response to ActD treatment that contain MOE ASOs in close proximity to the paraspeckle protein NONO^37^.

**Extended Data Fig. 10:**
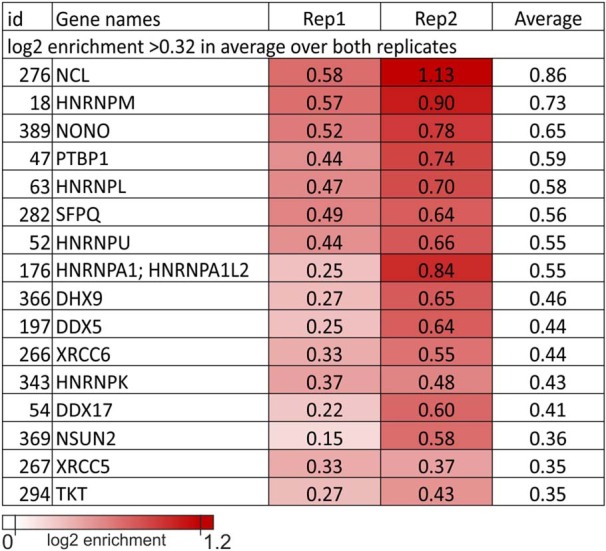
isASO-ID. Detecting Actinomycin D-induced changes of the ASO-protein interactome. SILAC-MS/MS for BG-ASO (20mer MOE) at 15 nM treated with 1.5 µg/ml Actinomycin D for 1 h prior fixation against 15 nM BG-ASO untreated (corresponding to Fig. 5e). Extended table of proteins enriched by treatment with Actinomycin D after transfection with 15 nM BG-MOE. Shown are the normalized values from two replicates with swapped SILAC labeling and the log2 of the average thereof. Proteins with an enrichment >0.32 log2 units in average over both replicates are displayed.

Indeed, microscopy confirmed the shift of the MOE ASO (15 nM) and NONO into perinucleolar cap structures in response to ActD treatment, accompanied by biotin deposition at the rim of the nucleoli (Fig. 5c, arrow a). We determined the BG–MOE ASO interactome against the control MOE under ActD treatment (Fig. 5d) and found, compared to the analog experiment with the same ASOs in the absence of ActD (Extended Data Fig. 9a), an increased enrichment >1 log_2_ unit of various P-body components (for example, HNRNPM and DHX9) and paraspeckle proteins (for example, SFPQ and NONO). When we plotted the BG–MOE ASO with ActD treatment against the BG–MOE ASO in absence of ActD (Fig. 5e and Extended Data Fig. 10), we observed, as expected, the enrichment (>0.4 log_2_ units) of several members of paraspeckle proteins (SFPQ, NONO, HNRNPK and HNRNPA1). Furthermore, we found various P-body components (for example, DHX9, XRCC5/6 and HNRNPM/U) and further RNA-binding proteins enriched. All these proteins are in accordance with recent literature on perinucleolar cap structure induced by ActD^37,46,47^.

Overall, these experiments highlight that isASO-ID allows the direct detection of dynamic changes of ASO–protein interactions, which are induced by stress inside the living cell, which was not possible with prior art approaches working in cell lysate^7,36^.

## Discussion

Drug-ID is a novel approach to profile drug–protein interactions in a drug-centric way without the need for prior knowledge of candidates. With respect to small-molecule drugs, Drug-ID might well complement existing methods^17,19^. The high efficiency of the method allows the profiling of drug–protein interactions at physiologically relevant drug concentrations inside the cell. Furthermore, proximity biotinylation not only identifies direct binders but also allows the detection of many components of multi-protein complexes, as exemplified here for the HDAC complexes. The expression of the biotin ligase could be restricted to specific tissues—for example, in future in vivo applications^30^—or can be restricted to subcellular compartments, as exemplified here by the highly selective detection of cytosolic HDAC6. However, the flipside for the latter is that binders will not be identified in compartments where the ligase is absent. Furthermore, binders can be missed out if a target protein is difficult to be biotinylated—for example, due to sequestration or lack of lysine residues or if a component of a target complex is already heavily biotinylated in the background, thus masking biotinylation due to the probe.

For the fast-growing field of ON therapeutics^10,11^, however, Drug-ID and, in particular, isASO-ID break new ground. For the first time, we can follow the interactome of ASOs with numerous endogenous proteins at the pharmacologically relevant ASO concentration directly inside living cells. Our technology does not require prior knowledge of potential candidates and does not rely on the (over)expression of such candidates as luciferase fusions, as required for Nano-BRET^45^ assays. Drug-ID/isASO-ID may allow the correlation of effects—for example, subcellular localization, aggregation, filament formation and response to stress—with important pharmacological properties, such as the knockdown efficacy and toxicity, directly with the protein interactome of the drug. The detected change in the ASO protein interactome toward paraspeckle (NONO/SFPQ) and P-body (DHX9 and XRCC5/6) proteins upon ActD-induced stress showcases this clearly. Furthermore, the approach enables the side-by-side comparison of various key elements of ON drug optimization^1,8^, such as dose and chemistries, as exemplified here with the interaction profiling at various ASO concentrations around the pharmacologically relevant dose and the comparison of LNA versus MOE wings at two different ASO lengths. However, the findings have to be interpreted with care. Given the estimated 10-nm labeling radius, not every hit is a direct binder. In the case of ASO drugs, their tendency to form aggregates and foci within the cell leads to very large numbers of detected hits, which most likely contain only a small number of direct interaction partners. The applied ASO doses can be directly correlated with knockdown efficiencies and cellular toxicity; however, the actual intracellular ASO concentration remains undetermined. Thus, concentration-dependent interaction profiles do not allow the determination of binding constants inside the cell directly. However, in comparison to the 2′-MOE ASO, we found that the 2′-F ASO bound the same protein interactors with higher enrichment factors at low ASO concentration, which is in accordance with the stronger affinity of the 2′-F ASO for such binders and, thus, might reflect differences in binding constants. As promiscuous protein binding of ASOs often leads to unfavorable clinical properties^9^, a better understanding of this, also in terms of structure^48^, has led^14,49^ and will likely lead to further improvements in the clinics. Although the in vivo Drug-ID protocol gave high-confidence hits, which were all in accordance with reports from the literature, the number of hits was comparably low. By establishing the low-background in situ variant called isASO-ID, we could address this issue. Moreover, the in situ protocol has further advantages. It will enable the following of fast processes, as the fixation time (min), and not the biotinylation time, is decisive. This might enable the study of fast processes, such as the ASO uptake process^50^. Furthermore, the need for genetic engineering of the target cell is not applicable, simplifying the procedure. Finally, the biotin ligase can (theoretically) access all compartments of a cell, likely including organelles, endosomes and other vesicles, without the need to engineer transgene expression in those compartments. Potentially, formaldehyde fixation could be problematic as it might compete for biotinylation, as both reactions take place at lysine residues. To asses this, we stained cells fixated with formaldehyde or ethanol with an NHS ester of a fluorescent dye (Supplementary Fig. 15). Compared to ethanol fixated cells, no decrease in fluorescence signal was visible for the settings used in the isASO-ID protocol, indicating sufficient lysine availability for biotinylation.

Overall, we expect our Drug-ID/isASO-ID protocols to work for other classes of ON drugs, too^1,8^, including splice-switching ASOs, siRNAs, antimiRs and ADAR-recruiting ASOs^51^. This may open new insights that are crucial for the development of clinically successful ON drugs.

## Methods

### Drug compounds

JQ1 (BLDpharm), valproic acid (Sigma-Aldrich) and Tacedinaline (Sigma-Aldrich) were obtained commercially and used without further purification. JQ1-CA was synthesized starting from JQ1-carboxylic acid and 2-(2-((6-chlorohexyl)oxy)ethoxy)ethanamine. Synthesis of SAHA-CA was carried out as described by Friedman Ohana et al.^26^, and the synthesis of Amide-CA was carried out in analogy. Synthesis and characterization of JQ1-CA and Amide-CA can be found in Supplementary Note 1.

### Synthesis of BG–ASOs, CA–ASOs and Atto594–ASOs

ASOs were obtained from Eurogentec in SEPOP desalted quality or from Biospring in HPLC purified quality. All ASO sequences, modification patterns and modified versions thereof can be found in Supplementary Table 3. For attachment of the Atto594 dye or the BG/CA moiety, the ASO contained either an additional C6 5′-aminolinker or a C7 3′-aminolinker. Before coupling reactions, desalted ASOs were precipitated with NaCl in ethanol and solved in RNase-free water (6 μg μl^−1^). BG-modified and CA-modified ASOs were synthesized and PAGE purified as recently described in detail^52^. The synthesis and the molecular structure of the BG-linker and CA-linker^53^ moieties after coupling is shown in Supplementary Note 1. For Atto594 labeling, Atto594-OSu ester (AttoTec) was solved in DMSO, and 2 µl of this stock solution (7 mM) was incubated with 2 µl of amino-modified ASO (6 µg μl^−1^) in 16 µl of labeling buffer (20 parts PBS, pH 7.4, plus 1 part 0.2 M aqueous sodium bicarbonate, pH 9) at room temperature overnight. ASOs were precipitated with NaOAc in ethanol and used without further purification. Labeling efficiency was determined by absorbance measurement at 260 nM and 603 nM (Atto594, ε603nm = 120 mM^−1^cm^−1^) or 500 nM (Atto488, ε603nm = 90 mM^−1^cm^−1^) and was more than 90% for all ASOs.

### Subcloning of NLS-Halo-eGFP-BASU, NLS-Halo-eGFP-BioID and SNAP-eGFP-BASU

The open reading frame (ORF) for the biotin ligase BASU, codon-optimized for mammalian expression, was obtained from commercial gene synthesis (GeneArt). For the generation of Flp-In T-REx 293 cells, BASU and BioID were subcloned into the pcDNA5 vector (Thermo Fisher Scientific). Furthermore, they were N-terminally tagged with an SV40 nuclear localization sequence, with a HaloTag (Promega) and with eGFP to obtain pcDNA5-NLS-Halo-eGFP-BASU and pcDNA5-NLS-Halo-eGFP-BioID constructs, respectively. The full coding sequences can be found in Supplementary Note 2. To generate transgenic HeLa cell lines, the PiggyBac-transposon system was used. For this, the XLone-GFP vector was purchased from Addgene (cat. no. 96930), and the ORF of the blasticidin resistance gene (Bsd) was replaced with a puromycin resistance gene (PuroR) by restriction cloning. Afterwards, the GFP ORF was entirely replaced by the SNAP-eGFP-BASU fusion ORF, resulting in the XLone-Puro_SNAP-eGFP-BASU plasmid. The full sequence can be found in Supplementary Note 2. A mammalian codon-optimized PiggyBac-transposase (mPB) gene^54^, was subcloned into a pcDNA3.1 backbone, resulting in the pcDNA3.1_mPB plasmid.

### Cell culture

If not stated otherwise, cells were cultivated in DMEM (Thermo Fisher Scientific) supplemented with 10% FBS (Thermo Fisher Scientific) at 37 °C with 5% CO_2_ in a water saturated steam atmosphere. For sub-cultivation and seeding, cells were washed with PBS, detached with 0.25% trypsin/EDTA (Sigma-Aldrich) and resuspended in fresh DMEM/FBS. Sub-cultivation was performed every 3–5 d.

### Creation of NLS-Halo-eGFP-BASU and NLS-Halo-eGFP-BioID Flp-In T-REx 293 cells

Creation of HEK293T Flp-In T-REx cell line generation was performed as described previously^21^. In brief, 4 × 10^6^ parental Flp-In T-REx 293 cells were seeded in DMEM/FBS + 100 µg ml^−1^ zeocin + 15 µg ml^−1^ blasticidin (DMEM/FBS/Z/B) in a 10-cm dish. After 24 h, medium was replaced with DMEM/FBS, and cells were transfected with 9 µg of pOG44 plasmid and 1 µg of the respective pcDNA 5 vector using 30 µl of Lipofectamine 2000 (Thermo Fisher Scientific) in OptiMEM (Thermo Fisher Scientific) according to the manual. After 24 h, medium was replaced with DMEM/FBS/15 µg ml^−1^ blasticidin/100 µg ml^−1^ hygromycin (DMEM/FBS/B/H), followed by selection for approximately 2 weeks (medium change every 3 d). Then, stable cell lines were transferred to 75-cm^2^ cell culture flasks and subsequently cultivated in DMEM/FBS/B/H.

### Creation of SNAP-eGFP-BASU HeLa cells

We used the previously described all-in-one Tet-On 3G inducible PiggyBac plasmid XLone for generating stable, doxycycline-inducible transgenic HeLa cell lines^55^. For this, 2 × 10^5^ HeLa cells (DSMZ, no: ACC 57) were seeded in DMEM/FBS in a 12-well plate. After 24 h, medium was replaced with fresh DMEM/FBS, and 1,600 ng of XLone-puro_SNAP-eGFP-BASU and 400 ng of pcDNA3.1_mPB were transfected with 6 µl of FuGENE 6 (Promega) in OptiMEM. After 24 h, the cells were transferred to a 10-cm cell culture dish. Forty-eight hours later, the medium was replaced with DMEM/FBS/5 µg ml^−1^ puromycin (DMEM/FBS/P). After 5 d of selection, the medium was replaced by DMEM/FBS and subsequently cultured in DMEM/FBS.

### SILAC labeling

For SILAC labeling, DMEM (PAN-Biotech) deficient in lysine and arginine was supplemented with 10% dialyzed FBS (PAN-Biotech) and 1% penicillin–streptomycin. Additionally, ^12^C_6_, ^14^N_2_ L-lysine (Sigma-Aldrich) and ^12^C_6_, ^14^N_4_ L-arginine (Sigma-Aldrich) were added to ‘light’ medium; D_4_ L-lysine (Cambridge Isotope Laboratories) and ^13^C_6_ L-arginine (Silantes) were added to the ‘medium’ condition; and ^13^C_6_, ^15^N_2_ L-lysine (Cambridge Isotope Laboratories) and ^13^C_6_, ^15^N_4_ L-arginine (Silantes) were added to the ‘heavy’ medium. Flp-In T-REx 293 cells, HeLa XLone cells or HeLa wild-type cells were cultivated in the respective SILAC medium for a minimum of 10 d before experiments. For sub-cultivation, SILAC cells were washed twice with PBS and treated with 1 mM EDTA (pH 8, Gibco) in PBS (EDTA/PBS) until being fully detached.

### Comparison of biotinylation activity of biotin ligases via western blot

For transient transfection, 300,000 HEK293T cells were seeded in a 12-well plate in DMEM/FBS. After 24 h, medium was replaced, and cells were transfected with 500 ng of the respective pcDNA5 vector using 2 µl of Lipofectamine 2000/12-well (Thermo Fisher Scientific) in OptiMEM (Thermo Fisher Scientific). Biotin (1 mM in PBS, sterile filtered) was added to a final concentration of 50 µM at the indicated timepoint, and cells were harvested 24 h after transfection. For stable expression, 300,000 Flp-In T-REx 293 NLS-Halo-eGFP-BASU or NLS-Halo-eGFP-BioID cells were seeded in a 12-well plate in DMEM/FBS/B/H. After 24 h, doxycycline was added to a final concentration of 10 ng ml^−1^. Biotin was added to a final concentration of 50 µM at the indicated timepoint, and cells were harvested 24 h after doxycycline induction. For this, cells were washed once with cold PBS and detached in cold PBS by pipetting up and down. Lysis was performed in urea lysis buffer (8 M urea, 100 mM NaH_2_PO_4_, 10 mM Tris, pH 8.0) by drawing the solutions 12 times through a 19-gauge syringe. Cell debris was removed by centrifuging for 20 min at 20,000*g* at 4 °C in a pre-cooled table centrifuge. Then, 10 µg of total protein lysate was loaded for SDS-PAGE (4% stacking, 12% separating gel), and proteins were transferred on a PVDF membrane using a tank-blotting system at 30 V overnight. The membrane was washed once with Tris-buffered saline with 0.1% Tween 20 (TBST) and incubated with a streptavidin–alkaline phosphatase conjugate (KPL, medac, 1:2,000 in 1% BSA (Fraction V, Roche) in TBST) for 2 h at room temperature. After washing twice in TBST, the membrane was equilibrated in staining solution (0.1 M Tris-HCl, pH 9.5, 0.1 M NaCl, 0.05 M MgCl_2_) and treated with 2% NBT/BCIP stock solution (nitro blue tetrazolium chloride/5-bromo-4-chloro-3-indolyl phosphate, toluidine salt, Roche) in staining solution. The reaction was stopped by rinsing the membrane with water and letting it dry. Results are reported in Extended Data Fig. 1e,f and Fig. 5.

### Comparison of NLS-Halo-eGFP-BASU and NLS-Halo-eGFP-BioID biotinylation activity by microscopic imaging

Next, 200,000 Flp-In T-REx 293 cells expressing either NLS-Halo-eGFP-BASU or NLS-Halo-eGFP-BioID were seeded on poly-d-lysine hydrobromide (Sigma-Aldrich) coated coverslips (12 mm Ø) in a 24-well plate. For 24-h induction of the biotin ligase, 10 ng ml^−1^ doxycycline was added to the medium. In contrast, 4-h induction was achieved by treating the cells with 10 ng ml^−1^ doxycycline before seeding and removing doxycycline during trypsination. Biotin (1 mM in PBS, sterile filtered) was added to a final concentration of 50 µM at the indicated timepoint, and cells were fixed and stained 24 h after seeding. Cells were washed with PBS, fixed (3.7% formaldehyde/PBS, 20 min, room temperature), washed twice with PBS and permeabilized (0.1% Triton-X100/PBS, 20 min, room temperature). After two more washes with PBS, biotin was stained with 1 µg ml^−1^ streptavidin-Atto594 (Sigma-Aldrich), and nuclei were stained with NucBlue (Thermo Fisher Scientific, 1:200) in PBS for 60 min at room temperature. The slides were rinsed with PBS, mounted in Fluorescence Mounting Medium (DAKO) and subjected to fluorescence microscopy as described below. Results are reported in Fig. 1b, Extended Data Fig. 1a,b and Supplementary Fig. 1.

### Probing small molecule–protein interactions in Flp-In T-REx 293 cells

SILAC-labeled Flp-In T-REx 293 NLS-Halo-eGFP-BASU cells were induced by the addition of 10 ng µl^−1^ doxycycline to the growth medium 4 h before seeding or while seeding. Cells were washed twice with PBS and detached with EDTA/PBS. The cells were collected in SILAC growth medium, spun down (50*g*, 5 min) and resuspended in fresh SILAC medium to remove residual doxycycline. Then, 1.5 × 10^6^ cells were seeded into a six-well plate in 2.5 ml of SILAC medium (typically two wells per condition). Eighteen hours after seeding, cells were treated with the drug–CA conjugate as indicated or DMSO. For competition experiments, the competitor was added at the indicated concentration together with the CA-conjugated probe. Two hours later, biotinylation was started by adding biotin to a final concentration of 50 µM (1 mM biotin stock in PBS). Biotinylation was carried out for 4 h at 37 °C. Cells were washed once with cold PBS and detached in 500 µl of PBS by pipetting up and down. Cells were spun down (300*g*, 5 min); PBS was carefully removed; and lysis buffer was added (225 µl per well). If not indicated otherwise, the lysis buffer contained 50 mM Tris-HCl, 0.8% Igepal-CA630, 5% glycerol, 150 mM NaCl, 1.5 mM MgCl2, pH 7.5. Then, 1 mM DTT and one tablet of Roche cOmplete mini protease (EDTA-free) inhibitor per 25 ml were added just before usage^15^. Cells were homogenized using shear-force by drawing the solutions 12 times through a 19-gauge syringe. Cell debris was removed by centrifuging for 20 min at 20,000*g* and 4 °C. The supernatant was carefully transferred to a fresh tube, snap frozen and stored at −80 °C. The protein concentration was determined using a Pierce BCA Protein Assay Kit. A summary of all Drug-ID experiments can be found in Supplementary Table 2.

### Probing ASO–protein interactions in HeLa cells

In a typical Drug-ID experiment for ASO interactome profiling, 250,000 HeLa cells expressing SNAP-eGFP-BASU in a doxycycline-dependent manner were seeded into a six-well plate (typically two wells per condition). After 20 h, doxycycline was added to a final concentration of 500 ng ml^−1^. Four hours later, the cells were washed twice with PBS, and the medium was replaced with fresh SILAC DMEM without doxycycline. The ASOs were transfected in the indicated concentrations with 10 µl of Lipofectamine 3000/six-well (Thermo Fisher Scientific) in SILAC DMEM without FBS. All ASO concentrations refer to the final concentration within the well. Sixteen hours after transfection, biotinylation was triggered by adding biotin to a final concentration of 50 µM (1 mM biotin stock in PBS and stored for up to 6 months). Biotinylation was carried out for 4 h at 37 °C. Afterwards, the plates were put on ice, washed once with cold PBS and harvested in 225 µl of lysis buffer (4 °C) per six-well using a cell scraper. Proteins were extracted as described above for the probing of small-molecule interactions. The protein concentration was determined using the Pierce BCA Protein Assay Kit. A summary of all Drug-ID experiments can be found in Supplementary Table 2. All ASO sequences and their modifications can be found in Supplementary Table 3.

### Pulldown and SDS-PAGE

For Drug-ID experiments, lysates from three different SILAC conditions (L, M and H) were pooled (typically 200–333 µg of protein per condition) and enriched with Streptavidin Magnetic Sepharose (GE Healthcare, binding capacity >300 µg of biotinylated BSA per milliliter of medium slurry) overnight at 4 °C using an end-over-end rotator. Then, 200 µl of bead slurry was used for 1 mg of total protein extract. Note: if biotinylation activity is increased—for example, by longer doxycycline induction or longer biotin incubation—the amount of Streptavidin Magnetic Sepharose has to be adapted (increased) to capture all biotinylated proteins. Because the protein concentration could not be determined for the isASO-ID experiments, 180 µl of beads was used per milliliter of lysate before dilution with PBS.

In a typical experiment, beads were washed twice with 0.5 ml of wash buffer 1 (2% SDS, all washing steps with 0.5 ml of buffer, 5 min, at room temperature), once with wash buffer 2 (0.1% w/v deoxycholate, 1% w/v Tween 20, 350 mM NaCl, 1 mM EDTA, pH 8.0), once with wash buffer 3 (0.5% w/v deoxycholate, 0.5% w/v Tween 20, 1 mM EDTA, 250 mM LiCl, 10 mM Tris-HCl, pH 7.4), once with wash buffer 4 (50 mM NaCl, 50 mM Tris-HCl, pH 7.4) and three times with 50 mM ammonium bicarbonate. Under these conditions, only covalently biotinylated proteins were retained, and it is referred to as ‘harsh wash’ in this manuscript. In experiments aiming to capture more interacting proteins, the beads were washed five times with 0.5 ml of cold lysis buffer (50 mM Tris-HCl, 0.8% Igepal-CA630, 5% glycerol, 150 mM NaCl, 1.5 mM MgCl2, pH 7.5, 1 mM DTT, cOmplete mini protease EDTA-free inhibitor) containing additional 0.2% NP-40, referred to as ‘soft wash’ in this manuscript. After the last washing step, proteins were eluted by boiling the beads twice with 20 µl of elution buffer (10 mM Tris, pH 7.4, 2% SDS, 5% β-mercaptoethanol and 2 mM biotin)^56^. Then, 6× Laemmli buffer (0.4 M SDS, 60 mM Tris, pH 6.8, 6.5 M glycerol, 0.6 M dithiothreitol, 0.9 mM bromophenol blue) was added, and samples were boiled for 5 min at 95 °C. Samples were loaded on a Novex 8–16% Tris-Glycine Mini Gel (Thermo Fisher Scientific). For western blot detection, proteins were run on the Novex 8–16% Tris-Glycine Mini Gel (Thermo Fisher Scientific) and transferred on a PVDF as described below. For MS, gels were run until the bromophenol blue band migrated approximately 3 cm into the gel. The gels were stained using InstantBlue Safe Coomassie Stain (Expedeon) for 2 h and destained by washing several times with water. Using a scalpel, the whole lanes of each sample were excised. The streptavidin band (running at approximately 17 kDa) was separated and measured independently.

### In-gel digest and MS

Coomassie-stained gel pieces were digested in gel with trypsin^57^, and desalted^58^ peptide mixtures were analyzed on an Easy-nLC 1200 coupled to a Q Exactive HF mass spectrometer (both Thermo Fisher Scientific) as described previously^26^ with slight modifications: peptide mixtures were separated using a 57-min and 87-min segmented gradient, respectively, of 10%–33%–50%–90% of HPLC solvent B (80% acetonitrile in 0.1% formic acid) in HPLC solvent A (0.1% formic acid) at a flow rate of 200 nl min^−1^. In each scan cycle, the seven most intense precursor ions were sequentially fragmented using higher-energy collisional dissociation (HCD) fragmentation. In all measurements, sequenced precursor masses were excluded from further selection for 30 s. The target values for MS/MS fragmentation were 10^5^ charges and, for the MS scan, 3 × 10^6^ charges.

Acquired MS spectra were processed with the MaxQuant software package version 1.5.2.8 (Drug-ID) or version 1.6.14.0 (isASO-ID) with integrated Andromeda search engine^59,60^. Database search was performed against a target-decoy *Homo sapiens* database obtained from UniProt, and commonly observed contaminants were rejected (downloaded 20 December 2017 (Drug-ID) and 7 October 2020 (isASO-ID)). In addition, depending on the experiment, data were searched against the sequences of NLS-Halo-eGFP-BASU, NES-Halo-eGFP-BASU, SNAP-eGFP-BASU and NLS-SNAP-eGFP-BASU, respectively. For isASO-ID, data were searched against the sequences of Halo-BASU-His or SNAP-BASU-His, depending on the experiment. In database search, full trypsin digestion specificity was required, and up to two missed cleavages were allowed. Carbamidomethylation of cysteine was set as fixed modification, and protein N-terminal acetylation and oxidation of methionine were set as variable modifications. Initial precursor mass tolerance was set to 4.5 ppm and 20 ppm at the MS/MS level. The amino acids Lys4/Arg6 and Lys8/Arg10 were defined as medium and heavy labels, respectively. For protein group quantitation, a minimum of two quantified peptides were required. Peptide, protein and modification site identifications were reported at a false discovery rate (FDR) of 0.01, estimated by the target-decoy approach^61^.

Perseus software^62^ (version 1.6.1.3) was used for calculation of the significance B (psigB) for each protein ratio with respect to the distance of the median of the distribution of all protein ratios as well as protein intensities^59^. All proteins in a pairwise comparison with psigB <0.01 and psigB <0.05, respectively, were considered to be differentially regulated. Scatter plots were created with Perseus software (version 1.6.1.3 or version 1.6.15.0) and modified with CorelDraw 2017. An overview of all SILAC–MS/MS experiments performed in this study can be found in Supplementary Table 2.

### Uptake of JQ1-CA and SAHA-CA in Flp-In T-REx 293 NLS-Halo-eGFP-BASU cells

Transgene expression in Flp-In T-REx 293 Halo-eGFP-BASU cells was induced by adding 10 ng µl^−1^ doxycycline to the growth medium 4 h before seeding. Cells were washed with PBS, detached with Trypsin/EDTA, collected in DMEM/FBS, spun down (50*g*, 5 min) and resuspended in fresh DMEM/FBS to remove residual doxycycline. Then, 3 × 10^5^ cells were seeded into a 24-well plate in DMEM/FBS. Eighteen hours after seeding, the cells were treated with the respective drug–CA conjugate (JQ1-CA or SAHA-CA) with the indicated concentration for 90 min. Then, TMR-CA was added to a final concentration of 1 µM and incubated another 30 min. Lysis was carried out similar to a Rapid Protein Extraction (RPE) protocol described in literature^63^. In brief, cells were washed once with PBS and lysed in 50 µl of quick lysis buffer, which was prepared by mixing 5 parts of lysis buffer (50 mM Tris-HCl, 0.8% Igepal-CA630, 5% glycerol, 150 mM NaCl, 1.5 mM MgCl2, pH 7.5, 1 mM DTT and one tablet of Roche cOmplete mini protease (EDTA-free) inhibitor per 25 ml) and 1 part of 6× Laemmli buffer. Lysates were immediately snap frozen and stored at −80 °C. Before SDS-PAGE, protein samples were boiled for 15 min at 98 °C while shaking at 1,500 r.p.m. to break down DNA. Then, 12.5 µl of sample was loaded on a Novex 8–16% Tris-Glycine Mini Gel (Thermo Fisher Scientific). Gels were disassembled and analyzed in a Fujifilm FLA-5100 fluorescence scanner using an excitation wavelength of 532 nm and recording the emission with a Cy3 emission filter (signal amplification = 450 V). Afterwards, proteins were transferred onto a PVDF membrane, and Halo-Tag and GAPDH or ACTB were detected as described in the subsection ‘Western blotting’. Results are reported in Fig. 1e and Supplementary Fig. 2a.

### Uptake of ASOs in HeLa SNAP-eGFP-BASU cells

Next, 50,000 HeLa SNAP-eGFP-BASU cells were seeded into a 24-well plate in DMEM/FBS 20 h before doxycycline induction (final concentration of 500 ng ml^−1^). Four hours later, cells were washed twice with PBS, and the medium was replaced with fresh DMEM/FBS without doxycycline. The ASOs were transfected in the indicated concentrations with 2 µl of Lipofectamine 3000 per well (Thermo Fisher Scientific) in OptiMEM (Thermo Fisher Scientific). All ASO concentrations refer to the final concentration within the well. After 15 h, *O*-acetylated BG–FITC was added to a final concentration of 5 µM and incubated for 1 h. Cell lysis and SDS-PAGE were performed as described for uptake of JQ1-CA and SAHA-CA in Flp-In T-REx 293 NLS-Halo-eGFP-BASU cells. Gels were analyzed in a Fujifilm FLA-5100 fluorescence scanner using an excitation wavelength of 473 nm and recording the emission with a FITC emission filter (signal amplification = 450 V). Results are reported in Fig. 2c and Supplementary Figs. 6b and 8a.

### Western blotting

After SDS-PAGE, protein samples were transferred onto PVDF membranes via western blotting using a wet tank blotting system (Mini Trans-Blot, Bio-Rad). PVDF membranes (Thermo Fisher Scientifc) were activated in methanol and equilibrated in transfer buffer (25 mM Tris hydrochloride, 190 mM glycine, 20% methanol). Protein transfer was performed at 4 °C with a constant current of 30–35 V for 16–18 h. Membranes were washed once with TBST and blocked with 5% non-fat dry milk (Bio-Rad) in TBST for 1 h at room temperature. Primary and secondary antibodies were diluted in 2.5–5% non-fat dry milk in TBST as listed in Supplementary Table 2. After each incubation with antibodies, membranes were washed three times with TBST. HRP-conjugated secondary antibodies (Supplementary Table 2) were developed using Clarity Max Western ECL substrate (Bio-Rad), and chemiluminescence was detected with a FUSION S2 Vilber Lourmat imaging system (Peqlab, VWR). If more than two proteins were analyzed on one blot (Fig. 2b and Supplementary Fig. 2b), detection was performed sequentially. For additional information on antibodies, see the Supplementary Information.

### Co-localization of SNAP-eGFP-BASU with Atto594–ASOs or biotin signal

Next, 50,000 HeLa SNAP-eGFP-BASU cells were seeded into a 24-well plate with 12-mm Ø coverslips. After 20 h, doxycycline was added to a final concentration of 500 ng ml^−1^. Doxycycline induction and transfection were carried out as described above in uptake of ASOs in HeLa SNAP-eGFP-BASU cells. All ASO concentrations refer to the final concentration within the well. Atto594–ASO was co-transfected in a 1:5 ratio to BG–ASO for concentrations ≤10 nM (for example, 10 nM BG–ASO + 2 nM Atto594–ASO). If concentrations higher than 10 nM were transfected, the amount of fluorescent Atto594–ASO was kept constant at 2 nM. After 20 h, cells were carefully washed with PBS, fixed (3.7% formaldehyde/PBS, 20 min, room temperature) and washed with PBS, and cell nuclei were stained with NucBlue (Thermo Fisher Scientific, 1:200) in PBS for 30 min at room temperature. The slides were rinsed with PBS, mounted in Fluorescence Mounting Medium (DAKO) and subjected to fluorescence microscopy as described below. Results are reported in Fig. 3e and Supplementary Fig. 8. For co-staining with streptavidin (Extended Data Figs. 3b and 7b), no fluorescent Atto594–ASO was co-transfected. Instead, biotin was added to a final concentration of 50 µM 16 h after transfection. Four hours later, cells were carefully washed with PBS, fixed (3.7% formaldehyde/PBS, 20 min, room temperature), washed twice with PBS and permeabilized (0.1% Triton X-100/PBS, 20 min, room temperature). After two more washes with PBS, biotin was stained with 1 µg ml^−1^ streptavidin-Atto594 (Sigma-Aldrich), and nuclei were stained with NucBlue (Thermo Fisher Scientific, 1:200) in PBS for 60 min at room temperature. The slides were rinsed with PBS, mounted in Fluorescence Mounting Medium (DAKO) and imaged as described below. Results are reported in Extended Data Figs. 3b, 5 and 7a,b and Supplementary Figs. 8 and 11.

For the isASO-ID protocol, Atto488-modified ASOs were used, and co-localization between ASO and biotinylation signal was investigated following the standard in situ Drug-ID protocol. Typically, biotinylation was performed for 4 h, but signal strength increases at least over 24 h. Results are reported in Figs. 4e,g and 5a,c.

### Immunofluorescence

For co-localization experiments of ASOs and proteins, immunofluorescence was carried out as follows. First, 50,000 HeLa wild-type cells were seeded into a 24-well plate on 12-mm Ø coverslips. Then, 24 h later, ASOs were transfected with the indicated final concentration in the well as described above for the HeLa SNAP-eGFP-BASU cells. Sixteen hours later, cells were washed with PBS, fixed (3.7% formaldehyde/PBS, 15 min, room temperature), washed with PBS, permeabilized (0.1% Triton X-100/PBS, 20 min, room temperature), washed with PBS and blocked (1% BSA/PBS, 1.5 h, room temperature). Primary antibodies were diluted 1:200 in 0.5% BSA in PBS and applied to the cells overnight at 4 °C. After washing with PBS, incubation with secondary antibodies (1:1,000) and NucBlue staining (1:100, 1 h, room temperature) was performed. Before mounting, cells were washed three times with PBS. A list of the antibodies used for immunofluorescence can be found in Supplementary Table 2. Results are shown in Fig. 5c.

### Fluorescence microscopy setup

Microscopy was performed on a Nikon Eclipse Ti2-E inverted fluorescent microscope equipped with a photometrics Prime 95B camera and a Lumencor AURA II light engine. All pictures were acquired using a ×60 oil objective (MRD31605, numerical aperture (NA) 1.4) and Olympus IMMOIL-F30CC immersion oil. The excitation wavelengths and corresponding filter sets used to record each channel are specified as follows. eGFP: 475 nM excitation light, excitation filter: Chroma ET500/×20, beamsplitter: Chroma Chroma T515lp, emission filter: Chroma ET535/30 m. Atto594: 575 nM excitation light, excitation filter: Semrock 585/29 BrightLine HC, beamsplitter: Chroma T610LPXR, emission filter: Semrock 650/60 BrightLine HC. DAPI: 390 nM excitation light, excitation filter: Chroma 89402x, beamsplitter: Chroma 89402bs, emission filter: Chroma 89402 m. Typically, a *z*-stack covering 6 µm (0.2-µm steps) was recorded. The images were deconvoluted using Nikon NIS Offline Deconvolution 4.51 software, and a maximum projection over three layers (covering 0.6 µM) was performed in ImageJ. For each channel, the same acquisition and contrast settings were applied within one sub-figure, and linear lookup tables were used.

### Quantification of *PTEN* knockdown via qRT–PCR

Next, 50,000 HeLa SNAP-eGFP-BASU cells were seeded into a 24-well plate. Transfection and doxycycline induction were carried out as described for the uptake of ASOs in HeLa SNAP-eGFP-BASU cells. Twenty hours after transfection, cells were lysed in 100 µl of passive lysis buffer (1×, Promega), and RNA was isolated using a Monarch RNA Cleanup Kit (New England Biolabs (NEB)). Remaining DNA was removed using a TurboDNase Kit (Thermo Fisher Scientific) according to the manual. Then, 300–600 ng of RNA was used for reverse transcription using a High-Capacity cDNA Reverse Transcription Kit (Thermo Fisher scientific), and the resulting cDNA was purified using a NucleoSpin Gel and PCR clean-up kit (Macherey Nagel). qPCR was carried out in an Applied Biosystems 7500 qPCR machine (96-well qPCR plate, 20 ng of cDNA per well). Fast SYBR Green Master Mix (Thermo Fisher Scientific) was used according to the manufacturerʼs protocol (10 μl of SYBR Green Mix, 7.2 μl nuclease-free water and 0.4 μl of each primer). The samples were measured in 2–3 technical replicates. *ACTB* and *GAPDH* served as internal standard genes. NE elution buffer (Macherey Nagel) was used as negative control for all targets. Primers against *PTEN* (PTEN_fw: 5′-AATGGCTAAGTGAAGATGACAATCAT-3′, PTEN_rev: 5′-TGCACATATCATTACACCAGTTCGT-3′), against *ACTB* (ACTB_fw: 5′-CGGGACCTGACTGACTAC-3′, ACTB_rev: 5′-TAATGTCACGCACGATTTCC-3′) and against *GAPDH* (GAPDH_fw: 5′-CAACAGCCTCAAGATCATCAG-3′, GAPDH_rev: 5′-CCTTCCACGATACCAAAGTTG-3′) were obtained from Sigma-Aldrich in HPLC purified quality. The program consisted of an initial denaturation step (95 °C, 20 s), followed by 40 cycles of denaturation (95 °C, 3 s) and annealing/elongation (60 °C, 30 s). A baseline correction was performed for each dataset using Life Technologies 7500 Software version 2.3 before the C(t) values were determined. The *PTEN* expression was referenced to *GAPDH* using Microsoft Excel 2016. For calculation of ΔC(t), the average C(t) obtained with *GAPDH* primers was subtracted from the average C(t) obtained with *PTEN* primers. ΔΔC(t) values and fold change (2^−ΔΔC(t)^) were determined in comparison to cells treated only with transfection reagent (Lipofectamine 3000, Thermo Fisher Scientific) from the ΔC(t) obtained for each ASO concentration. Results were displayed with GraphPad Prism 8 and are reported in Fig. 3i and Supplementary Figs. 9 and 10.

### ATP cell viability assay

Next, 4,500 HeLa SNAP-eGFP-BASU cells were seeded in a 96-well plate in DMEM/FBS. For each biological replicate, three separate wells were treated identically. After 24 h, the medium was replaced by fresh DMEM/FBS. ASOs were transfected in the indicated concentrations with Lipofectamine 3000 (0.4 µl of transfection reagent per 96-well plate, Thermo Fisher Scientific) in OptiMEM (Thermo Fisher Scientific). Twenty hours after transfection, cells were lysed in 50 µl of Passive Lysis Buffer (1×, Promega), and 10 µl of cell lysate was diluted with 30 µl of PBS in a LumiNunc 96-well plate (white, Thermo Fisher Scientific). Then, 40 µl of CellTiter-Glo 2.0 Reagent (Promega) was added, and the plates were incubated for approximately 10 min at room temperature to allow the luminescence signal to stabilize. Luminescence was measured in a Spark 10M plate reader (Tecan). The average of luminescence counts for each condition was averaged and divided by the average counts of untreated cells to determine the relative viability/ATP content in the wells using Microsoft Excel. Results were displayed with GraphPad Prism 8 and are reported in Fig. 3i.

### Overexpression of Halo-BASU-His and SNAP-BASU-His in *E. coli* (T7 Express Competent *E. coli*, NEB, C2566H) and protein purification

For purification of Halo-BASU-His or SNAP-BASU-His, a preparatory culture of *E. coli* was inoculated overnight at 37 °C shaking with 180 r.p.m. in LB medium supplemented with ampicillin. The next day, 500 ml of LB medium with ampicillin was inoculated with 15 ml of the preparatory culture and incubated at 37 °C at 150 r.p.m. Reaching an OD_600_ between 0.4 and 0.6, Halo-BASU-His or SNAP-BASU-His expression was induced by adding 0.4 mM IPTG to the medium, followed by 4-h incubation at 37 °C. After harvest by centrifugation (3,000*g*, 10 min, 4 °C), the pellet was snap frozen in liquid nitrogen and stored at −80 °C until purification.

For lysis, cells were thawed on ice, resuspended in 10 ml of NPI-10 buffer (50 mM NaH_2_PO_4_, 300 mM NaCl, 10 mM imidazole) and lysed by pulsed sonification (Branson Sonifier 250). Cell debris was removed by centrifugation (15,000*g*, 1 h, 4 °C) and subsequently filtered (0.45 µm). Enrichment of the His-tagged overexpressed protein was performed by nickel-cellulose beads. Then, 0.5 ml of beads per column (Macherey Nagel) was centrifuged (1,500*g*, 5 min), washed with 10 ml of water and equilibrated with 10 ml of NPI-20. The beads were then incubated with the cleared lysate for 1 h at 4 °C before washing with 10 ml of NPI-20 (50 mM NaH_2_PO_4_, 300 mM NaCl, 20 mM imidazole). The biotin ligases were eluted with 2 ml of NPI-250 (50 mM NaH_2_PO_4_, 300 mM NaCl, 250 mM imidazole), followed by buffer exchange using a centrifugal filter (Amicon Ultra 0.5 ml) to PBS. Protein concentration was determined by Bradford (Sigma-Aldrich) and aliquoted in 43% glycerol, followed by snap freezing. Until usage, the enzyme was stored at −80 °C. Final protein purity was confirmed by SDS-PAGE.

### isASO-ID

In a typical isASO-ID experiment, typically two wells on a six-well plate (250,000 cells per well) were used per replicate for each condition. Twenty-four hours after seeding, cells were transfected with the indicated amount of ASO as described for Drug-ID experiments. Only when indicated, cells were treated for the last hour before fixation with ActiD at a final concentration of 1.5 µg ml^−1^. Sixteen hours after transfection, cells were carefully washed with PBS, and then cells were fixed with 3.7% formaldehyde for 10 min at room temperature, washed with PBS and permeabilized gently with 0.1% Triton X-100 for 10 min at room temperature. After washing once with PBS, Halo-BASU-His or SNAP-BASU-His was diluted in 1 ml of conjugation buffer (2.5% glycerol, 50 mM NaCl, 10 mM Tris-HCl, 1 mM dithiothreitol) per well to a final concentration of 240 nM and incubated on the cells for 1 h at 37 °C. Unbound enzyme was washed away with PBS two times, and the biotinylation reaction was started by adding FISH biotinylation buffer (2 mM β-mercaptoethanol, 1.5 mM ATP, 1.5 mM MgCl_2_, 50 nM biotin in 20 mM sodium bicarbonate in PBS, pH 8.3). The reaction was stopped by rinsing the cells two times with PBS 24 h later. Cells were harvested in 225 µl of 1× Laemmli buffer per well. If not processed directly, lysates were snap frozen in liquid nitrogen and stored at −80 °C. Crosslinking was reversed by boiling the samples for 1 h at 98 °C. Before pulldown, samples were diluted in 1× PBS supplemented with one tablet of protease inhibitor per 25 ml. An overview of all SILAC–MS/MS experiments performed in this study can be found in Supplementary Table 1. All ASO sequences and their modifications can be found in Supplementary Table 3.

### Reporting summary

Further information on research design is available in the Nature Portfolio Reporting Summary linked to this article.

## Online content

Any methods, additional references, Nature Portfolio reporting summaries, source data, extended data, supplementary information, acknowledgements, peer review information; details of author contributions and competing interests; and statements of data and code availability are available at 10.1038/s41589-023-01530-z.

### Supplementary information

Supplementary Information Supplementary Figs. 1–15, Notes 1 and 2, Tables 1–3 and Appendix (uncropped western blot images).

Reporting Summary

### Source data

Source Data Fig. 1 Unprocessed western blot for Fig. 1e.

Source Data Fig. 2 Unprocessed western blot for Fig. 2b.

Source Data Fig. 3 Unprocessed western blot for Fig. 3c.

## Data Availability

The mass spectrometry proteomics data have been deposited to the ProteomeXchange Consortium via the PRIDE partner repository with the dataset identifier PXD045992. Further information on chemical synthesis, cloned constructs and additional data is available in the Supplementary Information. Source data are provided with this paper.
