# Supplementary material for: Profiling the interactome of oligonucleotide drugs by proximity biotinylation
Source: Nat Chem Biol. 2024 Jan 17;20(5):555–65. doi: 10.1038/s41589-023-01530-z (PMC11062921; doi:10.1038/s41589-023-01530-z)

nature chemical biology

Article

https://doi.org/10.1038/s41589-023-01530-z

Profiling the interactome of oligonucleotide drugs by proximity biotinylation

In the format provided by the authors and unedited

Content:

Supplementary Figures **2**

Supplementary tables
- **SILAC-MS/MS experiments** **18**
- **Antibodies used in this study** **22**
- **ASOs used in this study** **23**

Supplementary Note 1: Chemical synthesis
- **Synthesis of JQ-CA** **26**
- **Synthesis of Amide-CA** **28**
- **Synthesis of BG-linker and CA-linker** **30**

Supplementary Note 2: DNA sequences of inserts and plasmids **32**

Appendix
- **Uncropped images of Western blots and related** **42**

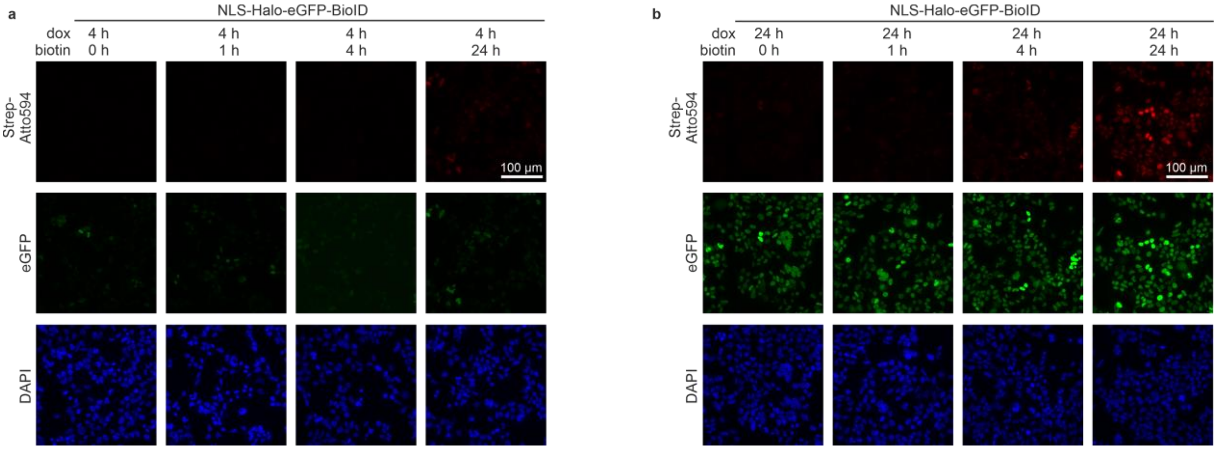

***Supplementary Figure 1**. **Characterization of HEK293T Flp-In T-REx NLS-Halo-eGFP-BioID cell line**. **a)** Expression and activity of the biotin ligase NLS-Halo-eGFP-BioID integrated into 293 Flp-In T-REx cells. **a)** Expression was induced with doxycycline for 4 h and followed by eGFP fluorescence (green channel). Cells were incubated with biotin (50 µM) for 0-24 h and biotin deposition was stained with Strep-Atto594 (red channel) after fixation and permeabilization of cells. Cell nuclei were stained with DAPI (blue channel). Acquisition and contrast settings are kept identical to Extended Data Fig. 1a+b for better comparison. **d)** Same as panel b but for HEK293T Flp-In T-REx NLS-Halo-eGFP-BioID (N=1 for panels a+b). For the experimental setup, see Extended Data Fig. 1c.*

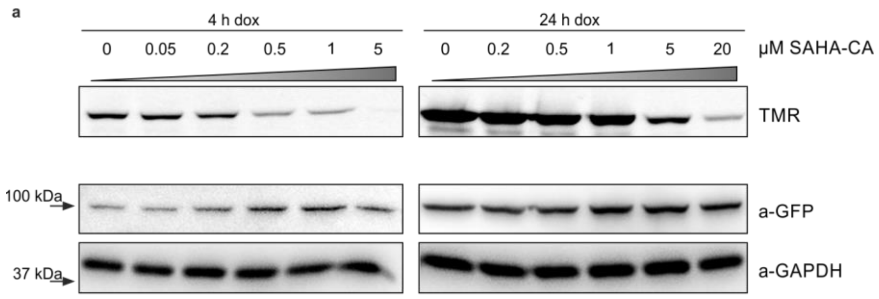

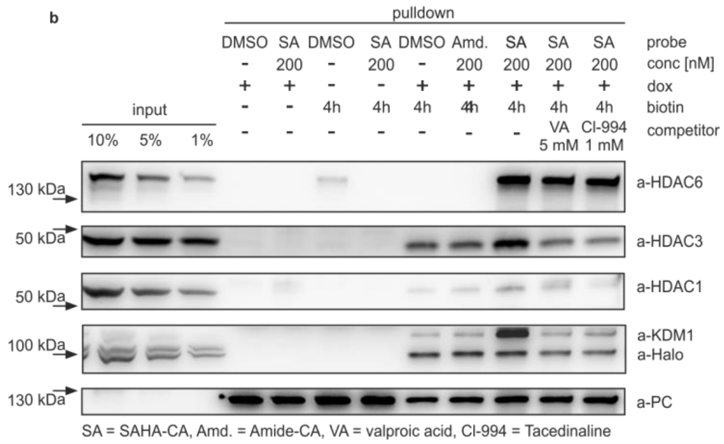

Supplementary Figure 2. Uptake of SAHA-CA probe and Western blot of selected HDACs and complex partners after enrichment with SAHA-CA probe and soft wash conditions in pulldown. a) *PAGE-analysis of SAHA-CA uptake into Flp-In T-REx 293 cells after transgene induction for 4 h or 24 h. Concentration-dependent conjugation of the SAHA-CA probe with the biotin ligase was visualized by progressive loss of conjugation of a competing fluorescent TMR-CA probe. Western blot against GAPDH and GFP served as loading controls. If NLS-Halo-eGFP-BASU is induced for 4 h, saturation of the Halo-tag can be observed for SAHA-CA concentrations >200 nM. After prolonged induction (24 h) saturation occurred at higher concentrations of SAHA-CA probe (>5 µM) (N=1).* ***b)*** *Biotinylation experiment with Western blot detection after streptavidin pulldown of 225 µg total protein lysate. Beads were washed only with lysis buffer (soft wash). Pyruvate carboxylase (PC) contains one biotin and served as loading/pulldown control. Halo-eGFP-BASU biotinylates itself and served as control for transgene expression and general biotinylation activity. HDAC6 biotinylation required the presence of the SAHA-CA probe, the transgene expression and is not sensitive to treatment with class-I HDAC-specific competitors (valproic acid 5 mM or Tacedinaline 2 µM). HDAC3 and the RCOR component KDM1 are enriched but also show slight enrichment in the absence of probe. The increased biotinylation of HDAC3 and KDM1 can be reversed by treatment with valproic acid or Tacedinaline. HDAC1 shows only a faint band in presence of transgene and biotin, and is slightly increased upon addition of SAHA-CA (N=1).*

a

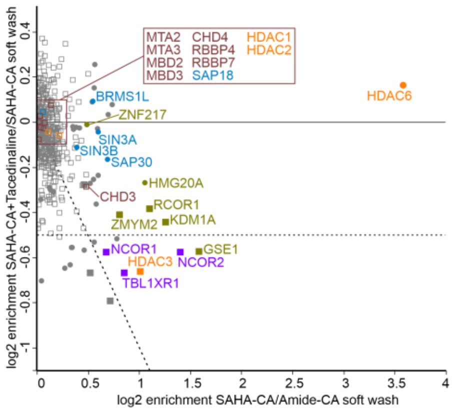

b

-2.5 log2 enrichment 2.5

id	Gene names	Complex	SAHA-CA/ Amide-CA	SAHA-CA+ Tacedinaline/ SAHA-CA
1260	HDAC1	CoREST,SIN,NuRD	0.22	-0.06
1599	HDAC2	CoREST,SIN,NuRD	0.10	-0.04
695	HDAC3	NCoR	**1.01**	**-0.66**
1895	HDAC6		**3.57**	0.17
1292	GSE1	CoREST	**1.58**	**-0.57**
1817	HMG20A	CoREST	**1.05**	-0.27
725	KDM1A	CoREST	**1.25**	**-0.44**
1926	RCOR1	CoREST	**1.10**	**-0.38**
1899	ZMYM2	CoREST	**0.80**	**-0.41**
742	ZNF217	CoREST	**0.48**	-0.01
745	NCOR1	NCoR	**0.67**	**-0.57**
278	NCOR2	NCoR	**1.40**	**-0.57**
1748	TBL1XR1	NCoR	**0.85**	**-0.66**
2057	SAP18	SIN	0.05	0.05
747	SAP30	SIN	**0.68**	-0.16
1691	SIN3A	SIN	**0.59**	-0.04
741	SIN3B	SIN	**0.38**	-0.11
673	CDK2AP1	NuRD	0.00	0.04
1228	CHD3	NuRD	0.48	-0.28
127	CHD4	NuRD	0.01	0.00
1500	GATAD2A	NuRD	-0.03	-0.02
1578	GATAD2B	NuRD	-0.14	0.07
1889	MBD2	NuRD	0.13	0.08
803	MBD3	NuRD	0.03	-0.02
1251	MTA1	NuRD	-0.09	0.05
774	MTA2	NuRD	0.01	-0.07
376	MTA3	NuRD	0.03	0.03
1217	RBBP4	NuRD, SIN	0.06	-0.03
1343	RBBP7	NuRD, SIN	0.07	0.00

Supplementary Figure 3*. *Competition experiment for the SAHA-CA interactome with Tacedinaline. a) *SILAC-MS/MS for the competition experiment 200 nM SAHA-CA against 200 nM SAHA-CA + 2 µM Tacedinaline (CI-994) under soft wash conditions. The enrichment with SAHA-CA over Amide-CA is displayed on the x-axis and the depletion upon addition of Tacedinaline is displayed on the y-axis (SAHA-CA+VA/ SAHA-CA).* ***b)*** *Enrichment of HDAC proteins and their previously established complex partners in SILAC-MS/MS experiment with 200 nM SAHA-CA under soft wash conditions and in competition with 2 µM Tacedinaline (CI-994). Only protein groups quantified in at least one replicate are shown. Statistically significant changes (p<0.01) in enrichment are highlighted in bold. Replicate 2 corresponds to the enrichment plot in panel a.*

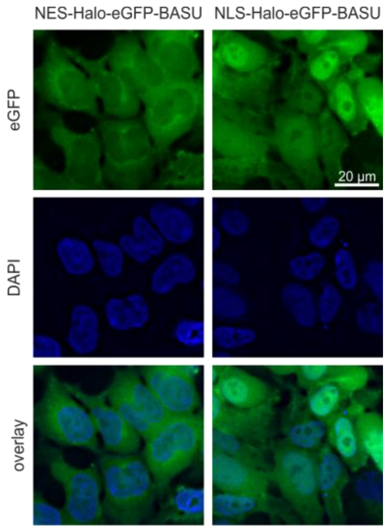

Supplementary Figure 4. Characterization of HEK293T Flp-In T-REx NES-Halo-eGFP-BASU and NLS-Halo-eGFP-BASU cell line*. Expression of the biotin ligase NES-Halo-eGFP-BASU and NLS-Halo-eGFP-BASU integrated into 293 Flp-In T-REx cells, respectively. Transgene expression was induced with doxycycline for 4 h and fixed 20 h later for visualization of the eGFP fluorescence (green channel). Cell nuclei were stained with DAPI (blue channel) (N=2).*

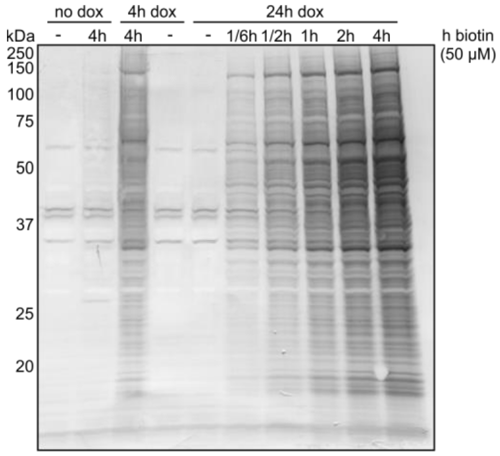

***Supplementary Figure 5*. Comparison of Halo-BASU activity in HEK293T Flp-In T-REx NLS-Halo-eGFP-BASU cells for 4h and 24 h doxycycline induction.** *NLS-Halo-eGFP-BASU expression was induced with 10 ng/ml doxycycline for 4 h or 24 h (4h dox, 24h dox) and biotin (50 µM) was added for the indicated amount of time (1/6 h to 4 h) 20 h after applying doxycycline to the cells. Cells were harvested in 1x Laemmli buffer followed by SDS-PAGE and Western Blot against biotinylated proteins (N=1).*

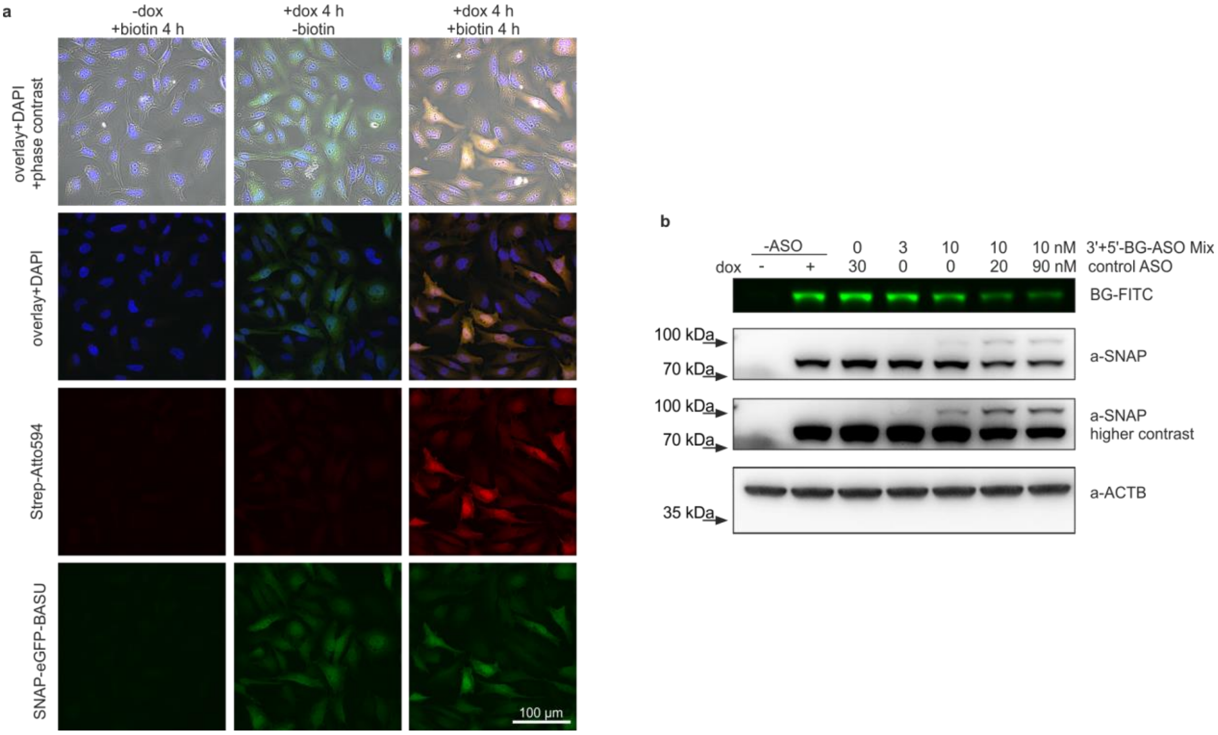

***Supplementary Figure 6**. **Drug-ID. Characterization of the HeLa XLone SNAP-eGFP-BASU cell line and uptake of BG-ASO. a)** Expression and activity of the biotin ligase SNAP-eGFP-BASU in HeLa cells. Transgene expression was induced for 4 h and visualized by eGFP fluorescence (green channel). Cells were incubated with biotin (50 µM) for 4 h and biotin deposition was stained with Strep-Atto594 (red channel) after fixation and permeabilization of cells. Cell nuclei were stained with DAPI (blue channel). All cells showed expression of SNAP-eGFP-BASU with an even distribution in cytoplasm and nucleus, although expression levels varied more than for HEK293T Flp-In T-REx NLS-Halo-eGFP-BASU 293 cells. Biotin deposition was dependent on doxycycline and biotin addition (N=1). **b)** Denaturing SDS-PAGE-analysis of BG-ASO (20mer MOE, 3´+5´mix) uptake (with Lipofectamine 3000) into transgenic HeLa cells. Concentration-dependent conjugation of the BG-ASO is visualized by progressive loss of conjugation of a competing fluorescent BG-FITC probe (green channel). Western blot against ACTB and SNAP-tag served as loading controls. Conjugation of the biotin ligase with the ASO led to a clear shift (ASO-conjugate). In contrary to main text Fig. 3c, the concentration of BG-ASO (3’+5’-mix) was kept at 10 nM and control ASO (lacking BG) was co-transfected to increase total ASO amount (N=1).*

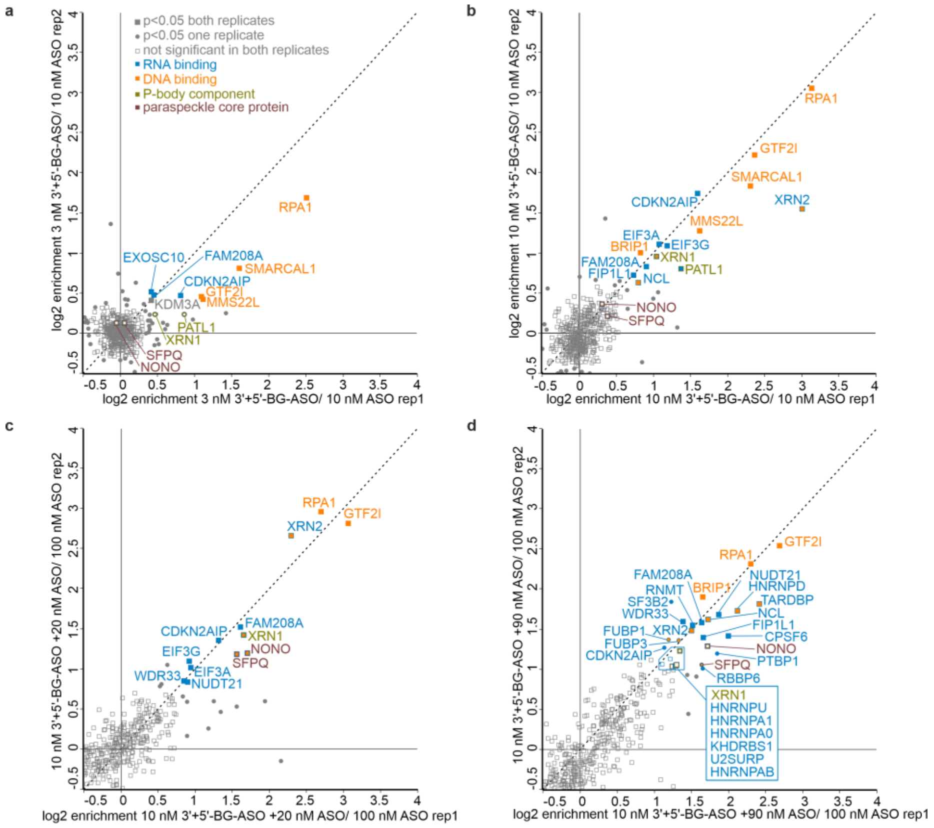

Supplementary Figure 7*. *Drug-ID. Dose-dependency of the ASO-protein interactome. a) *- **d)** SILAC-MS/MS for BG-ASO (20mer MOE, 3´+5´mix) against control ASO (lacking BG) at different ASO concentrations. Plotted was the enrichment (log2) of replicate 1 against replicate 2 with swapped SILAC labels. All proteins significantly (p<0.05) enriched in both replicates are highlighted and colorized according to their RNA- and/or DNA-binding function (blue = RNA binding, orange = DNA binding). Furthermore, selected paraspeckle core proteins (brown) and P-body components (gold) are highlighted. **a)** Enrichment with 3 nM BG-ASO (3´+5´mix) over 10 nM control ASO. **b)** Enrichment with 10 nM BG-ASO (3´+5´mix) over 10 nM control ASO. **c)** Enrichment with 10 nM BG-ASO (3´+5´mix) + 20 nM control ASO over 100 nM control ASO. **d)** Enrichment with 10 nM BG-ASO (3´+5´mix) + 90 nM control ASO over 100 nM control ASO.*

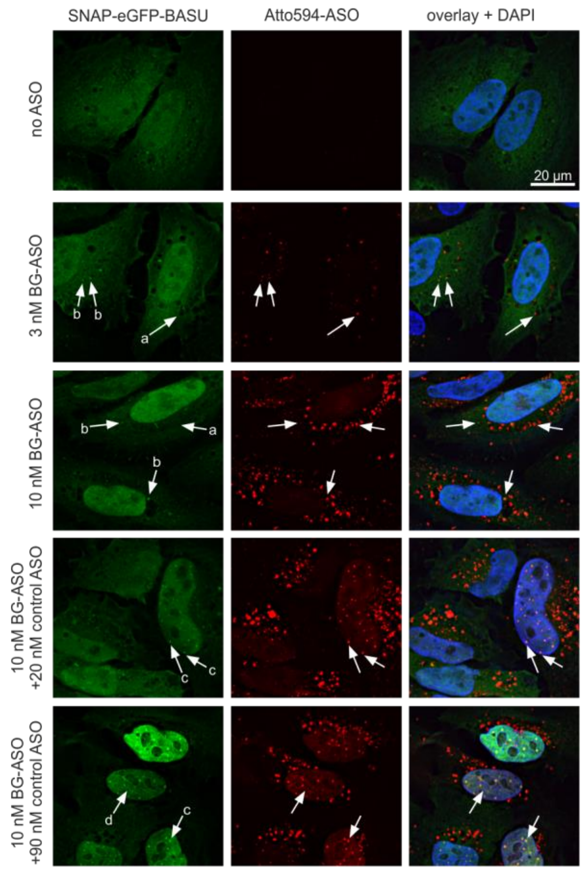

***Supplementary Figure 8**. **Drug-ID. ASO-SNAP-eGFP-BASU conjugate localization is dependent on total ASO concentration and not BG-ASO concentration.** Determining localization of ASO and biotin ligase via fluorescence microscopy. Analog to main text Fig. 3e, increasing amounts of BG-ASO (20mer MOE, 3´+5´-mix) were transfected with Lipofectamine 3000 into transgenic HeLa cells expressing SNAP-eGFP-BASU (green channel). To visualize ASO localization, 2 nM Atto594-labeled ASO (red channel) were spiked into transfections of BG- and control ASO. Nuclei were stained with DAPI (blue channel). In contrary to Fig. 3e, the concentration of BG-ASO (3'+5'-mix) was kept at 10 nM and control ASO (lacking BG) was co-transfected to increase total ASO amount. Just as observed for BG-ASO only (Fig. 3e), an increased nuclear sequestration and the formation of nuclear foci (arrow c) was observed for total ASO concentrations >10 nM. At very high ASO concentration (100 nM) nuclear filaments co-stained for biotin ligase and Atto594-ASO (arrow d) appear.*

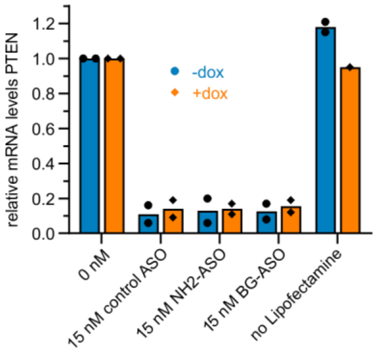

Supplementary Figure 9. Drug-ID. Conjugation of the biotin ligase to the ASO had no effect on the knockdown activity of the BG-ASO (20mer MOE). *Relative PTEN mRNA levels in transgenic HeLa cells were determined by quantitative RT-PCR 20 h after ASO treatment. Control ASO (lacking BG), NH2-ASO (3'+5' mix, carrying the terminal aminolinker, but no BG) or BG-ASO (3'+5' mix) were transfected (Lipofectamine 3000) at a concentration of 15 nM. The terminal aminolinker with or without BG moiety did not decrease the knockdown efficiency of the ASO. Also when SNAP-eGFP-BASU expression was induced with doxycycline prior to ASO transfection (4 h) the knockdown levels stayed identical for all ASOs (blue: no induction, orange: with induction).*

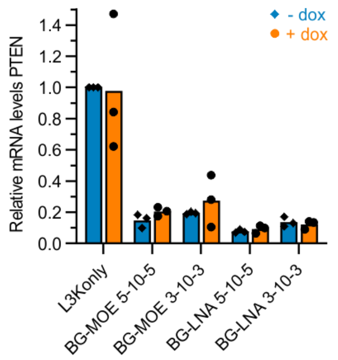

***Supplementary Figure 10*. Drug-ID. Knockdown activity of the ASOs with different lengths and containing either MOE or LNA wings.** *Relative PTEN mRNA levels in transgenic HeLa cells were determined by quantitative RT-PCR 20 h after ASO treatment. MOE- or LNA-modified ASOs of different length (16 or 20 nucleotides) were transfected (Lipofectamine 3000) at a concentration of 15 nM. For all four tested ASOs, knockdown efficiency was similar with the LNA performing slightly better. Induction of SNAP-eGFP-BASU with doxycycline (4 h) and conjugation to the ASOs did not affect knockdown efficiency (blue: no induction, orange: with induction).*

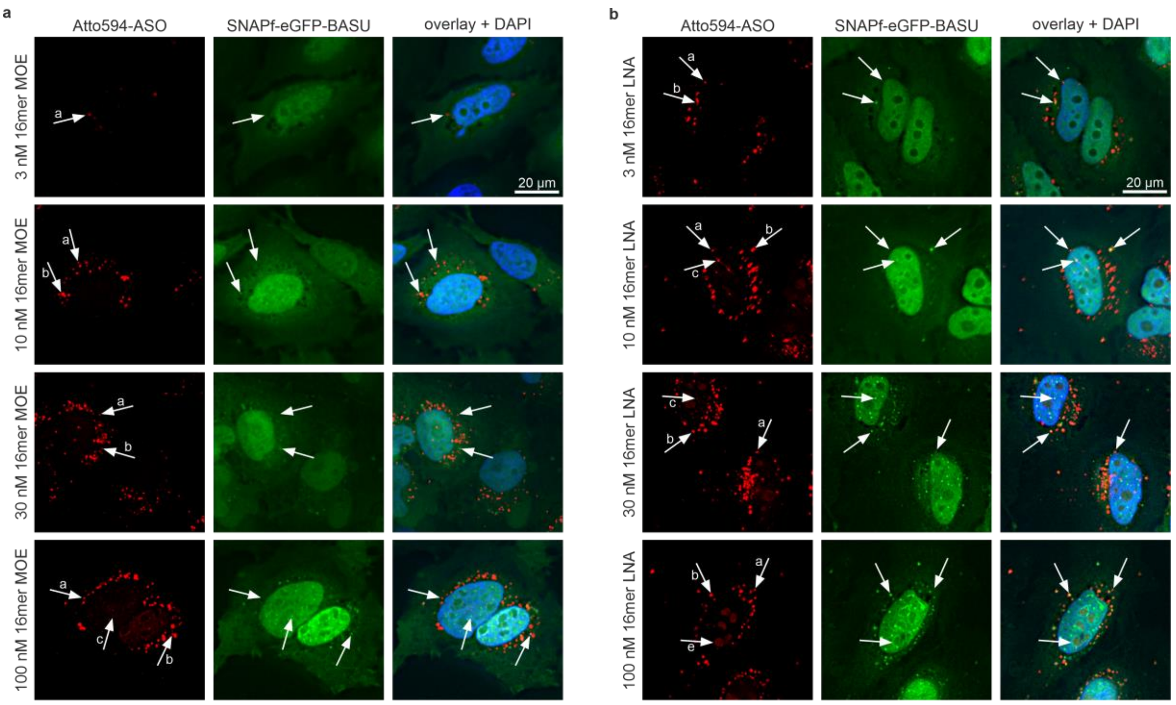

Supplementary Figure 11. Drug-ID. Localization of the ASO-SNAP-eGFP-BASU conjugate depends on ASO dose and ASO chemistry. *Determining localization of ASO and biotin ligase via fluorescence microscopy. Analog to Supplementary Fig. 5, increasing amounts of BG-ASO (16mer Gapmers containing MOE and LNA wings) were transfected with Lipofectamine 3000 into transgenic HeLa cells expressing SNAP-eGFP-BASU (green channel). To visualize ASO localization, 2 nM Atto594-labeled ASO (red channel) were spiked into transfections of BG-ASO. Nuclei were stained with DAPI (blue channel). As in Supplementary Fig. 5 the concentration of BG-ASO was kept at 10 nM and control ASO (lacking BG) was co-transfected to increase total ASO amount. ASOs not having accessed the nuclei (arrow a), cytoplasmic foci (arrow b), nuclear foci (arrow c), and nucleolar aggregates (arrow d) are indicated with white arrows in all panels.* ***a)*** *As observed for the 20mer MOE-modified ASO, nuclear sequestration and the formation of nuclear foci increased with higher concentrations. The shorter (16mer instead of a 20mer) MOE-modified ASO showed nuclear sequestration and formation of nuclear foci at higher concentrations compared to the longer ASO (Supplementary Fig. 5).* ***b)*** *The shorter (16mer) LNA ASO behaved similar as the 20mer (Extended Data Fig. 5), although to a weaker extent (N=1 for panels a+b). All microscopy and contrast settings are identical to Extended Data Fig. 5 for better comparison.*

		20mer MOE		20mer LNA		
id	Gene names	Rep1	Rep2	Rep1	Rep2	
log2 enrichment >0.6 and significant for at least one condition and replicate						
451	NCL	**1.60**	**1.64**	0.95	1.32	20mer MOE preferred
604	EIF3A	**1.72**	**1.33**	1.31	1.36	
556	EIF4G2	**1.53**	**1.50**	1.14	1.51	
817	FAM208A	**1.13**	**1.15**	0.36	0.59	
381	PRPF6	**1.62**	0.24	1.16	0.47	
689	RBBP6	**0.97**	0.85	0.72	1.01	
557	GTF2I	**2.17**	**2.23**	**3.69**	**4.00**	20mer LNA preferred
467	RPA1	**1.99**	**2.25**	**3.30**	**3.41**	
777	XRN2	**1.79**	**2.09**	**2.39**	**2.87**	
41	HNRNPUL1	0.66	0.41	**2.37**	**2.54**	
574	TGM3	**0.99**	0.60	1.10	**3.62**	
438	UNG; DKFZp781L1143	**0.96**	0.44	**2.37**	2.27	
799	CDKN2AIP	**1.55**	**1.61**	**2.11**	2.24	
562	HNRNPU	0.42	0.60	**2.08**	2.05	
723	ATXN2L	-0.26	0.30	**1.71**	2.14	
89	BCLAF1	**1.02**	**0.99**	**1.76**	1.94	
701	XRN1	**1.22**	0.61	**1.92**	1.67	
573	KHDRBS1	0.60	0.36	**1.64**	1.82	
803	SMARCAL1	**1.02**	**1.28**	**1.75**	1.57	
525	PLK1	0.42	-0.15	**1.62**	1.69	
754	ZFR	0.72	0.45	**1.71**	1.49	
382	PCF11	0.75	**0.86**	**1.60**	1.58	
218	FUBP1	**0.98**	0.30	1.56	1.48	
383	HEXIM1	0.01	0.29	**1.79**	1.05	
456	SFPQ	**0.88**	0.56	1.42	1.38	
670	FIP1L1	**1.27**	**1.21**	1.32	1.47	
775	WDR33	**1.23**	**1.30**	1.17	1.58	
190	HNRNPD	**1.01**	**0.95**	1.26	1.37	
598	SF3B2	0.74	**0.97**	1.01	1.51	
638	PRRC2B	**1.79**	0.11	1.01	1.43	
835	THRAP3	**1.11**	**0.97**	0.91	1.45	
250	HNRNPA1	**1.00**	**1.04**	0.89	1.17	
658	ADNP2	0.20	**1.13**	1.01	0.73	
818	CPSF3	0.57	**0.93**	0.40	1.23	

0 log2 enrichment 2.5

***Supplementary Figure 12**. **Drug-ID. Comparison of the MOE- and LNA-modified ASO interactome with NLS-SNAP-eGFP-BASU.** SILAC-MS/MS for BG-ASO (20mer containing either MOE or LNA wings) at 30 nM against Lipofectamine 3000 as in Extended Data Fig. 7. Extended table of enrichment profiles (log2, against Lipofectamine 3000) in both settings, differing in the chemical modifications of the applied ASOs. In all cases, 30 nM total ASO was transfected (10 nM BG-modified ASO and 20 nM NH_2-modified ASO). Shown are the values from two replicates with swapped SILAC labeling. Statistically significant changes (p<0.05) are highlighted in bold. Protein groups significantly enriched in at least one replicate (p<0.05) with a log2 enrichment of >0.6 in at least one replicate and condition are shown.*

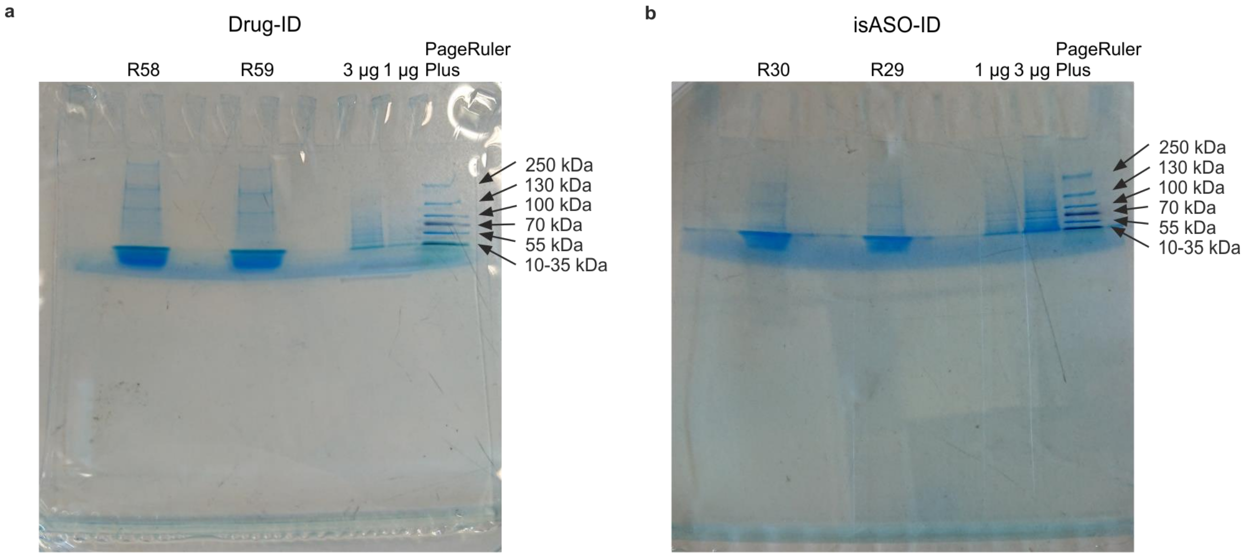

Supplementary Figure 13. SDS-PAGE after Streptavidin pulldown used for mass spectrometry analysis. *Shown are two representative gel images for Drug-ID (panel a, 20mer LNA versus MOE ASO) and isASO-ID (panel b, 20mer MOE ASO 50 nM versus 100 nM), respectively. As controls, 1 µg and 3 µg total protein lysate was loaded. In comparison to the loading controls, the amount of eluted protein after pulldown for the Drug-ID protocol is more than the 3 µg input lane, while for isASO-ID, the amount of eluted protein after pulldown was similar to the 1 µg input lane, highlighting the reduced background biotinylation with isASO-ID protocol. Note that the gel in panel b) was stronger stained compared to panel a) (N=1 for panels a and b).*

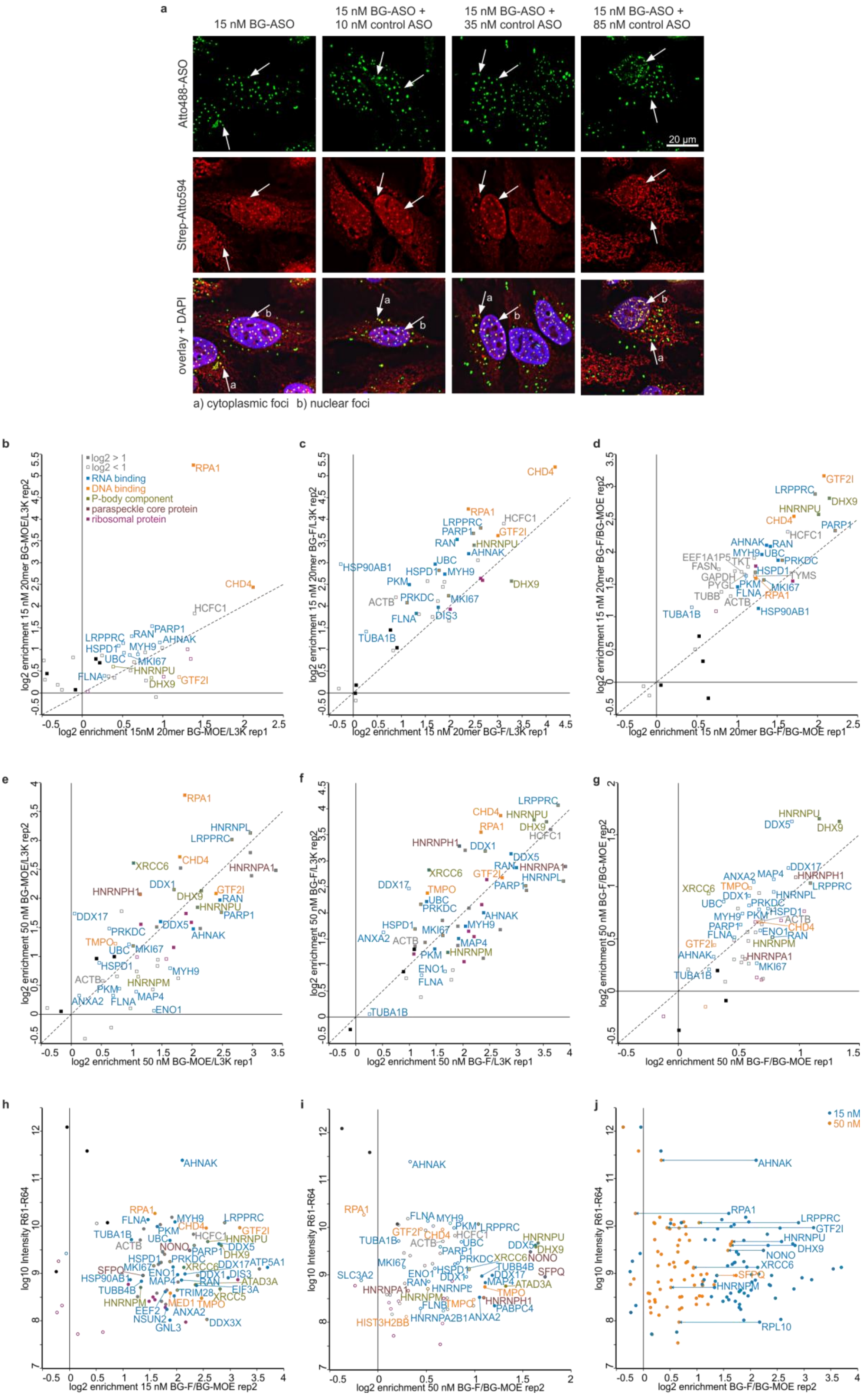
a
15 nM BG-ASO
15 nM BG-ASO + 10 nM control ASO
15 nM BG-ASO + 35 nM control ASO
15 nM BG-ASO + 85 nM control ASO
Atto488-ASO
Strep-Atto594
overlay + DAPI
20 µm
a) cytoplasmic foci b) nuclear foci
b
c
d
e
f
g
h
i
j
log2 > 1
log2 < 1
RNA binding
DNA binding
P-body component
paraspeckle core protein
ribosomal protein
log2 enrichment 15nM 20mer BG-MOE/L3K rep1
log2 enrichment 15 nM 20mer BG-MOE/L3K rep2
log2 enrichment 15 nM 20mer BG-F/L3K rep1
log2 enrichment 15 nM 20mer BG-F/L3K rep2
log2 enrichment 15 nM 20mer BG-F/BG-MOE rep1
log2 enrichment 15 nM 20mer BG-F/BG-MOE rep2
log2 enrichment 50 nM BG-MOE/L3K rep1
log2 enrichment 50 nM BG-MOE/L3K rep2
log2 enrichment 50 nM BG-F/L3K rep1
log2 enrichment 50 nM BG-F/L3K rep2
log2 enrichment 50 nM BG-F/BG-MOE rep1
log2 enrichment 50 nM BG-F/BG-MOE rep2
log2 enrichment 15 nM BG-F/BG-MOE rep2
log2 enrichment BG-F/BG-MOE rep2
log10 Intensity R61-R64
15 nM
50 nM

Supplementary Figure 14: Interactome of a 2'-Fluoro (F) versus 2´-MOE modified gapmer ASO. a) *Localization of ASOs and colocalization of biotin deposition was determined via fluorescence microscopy as described in Fig. 5a). 15 nM 2'-F-modified BG-ASO was transfected into HeLa cells using Lipofectamine 3000 for 24 h. To reach the indicated concentration (15, 25, 50, 100 nM), NH_2-ASO was added as indicated. To visualize ASO localization, 2 nM Atto488-labeled ASO (green channel) was spiked into transfections of BG-ASO. Biotin was stained with Atto594-Streptavidin (red channel) after the reaction. Cell nuclei were stained with DAPI (blue channel). White arrows indicate the appearance of structures with co-localization of Atto488-ASO and the biotin signal. Both ASOs show cytoplasmic foci (arrow a) and nuclear (arrow b) (N=1).* ***b)-g)*** *SILAC-MS/MS analog to Fig. 4b) and f) and Supplementary Fig. 16 for BG-ASO (20mer containing either MOE or F wings) at 15 nM and 50 nM against Lipofectamine 3000. Plotted was the enrichment (log2) of replicate 1 against replicate 2 with swapped SILAC labels. All proteins enriched in both replicates by more than one log2 unit are highlighted and colored according to their RNA- and/or DNA-binding function (blue = RNA binding, orange = DNA binding). Furthermore, selected paraspeckle core proteins (brown) and P-body components (gold) are highlighted.* ***b)*** *Enrichment with 15 nM BG-ASO (20mer MOE) over Lipofectamine 3000.* ***c)*** *Enrichment with 15 nM BG-ASO (20mer F) over Lipofectamine 3000.* ***d)*** *Enrichment with 15 nM BG-ASO (20mer F) over 15 nM BG-ASO (20mer MOE).* ***e)*** *Enrichment with 15 nM BG-ASO + 35 nM NH2-ASO (20mer MOE) over Lipofectamine 3000.* ***f)*** *Enrichment with 15 nM BG-ASO + 35 nM NH2-ASO (20mer F) over Lipofectamine 3000.* ***g)*** *Enrichment with 15 nM BG-ASO + 35 nM NH2-ASO (20mer F) over 15 nM BG-ASO + 35 nM NH2-ASO (20mer MOE).* ***h)*** *Enrichment with 15 nM BG-ASO (20mer F) over 15 nM BG-ASO (20mer MOE), the log2 enrichment of one replicate is plotted against the total intensity over all replicates.* ***i)*** *Enrichment with 15 nM BG-ASO + 35 nM NH2-ASO (20mer F) over 15 nM BG-ASO + 35 nM NHA-ASO (20mer MOE), the log2 enrichment of one replicate is plotted against the total intensity over all replicates.* ***j)*** *Two stacked plots. Enrichment with 15 nM BG-ASO (20mer F) over 15 nM BG-ASO (20mer MOE; blue) and 15 nM BG-ASO and 35 nM NH2-ASO (20mer F) over 15 nM BG-ASO + 35 nM NH2-ASO (20mer MOE; orange). Changes between the enrichments in both conditions are indicated with arrows (blue for reduction upon increased ASO dose, orange for increased enrichment upon increased ASO dose). Highlighted are selected known ASO interactors and proteins with notable changes. In general, almost all proteins showed stronger enrichment by 2'-F-modified ASO at 15 nM compared to the 2'MOE-modified ASO. Enrichment was lower at increased concentration (50 nM) when comparing 2'-F- and 2'-MOE-modified ASOs which is in concordance with the appearance of nuclear punctuate structures for the 2'-F-modified ASO at lower concentrations (15 nM) than for the 2'-MOE-modified ASO (compare panel a) and Fig. 5a). At 50 nM, both ASOs show similar nuclear accumulation, thus also showing a more comparable interactome.*

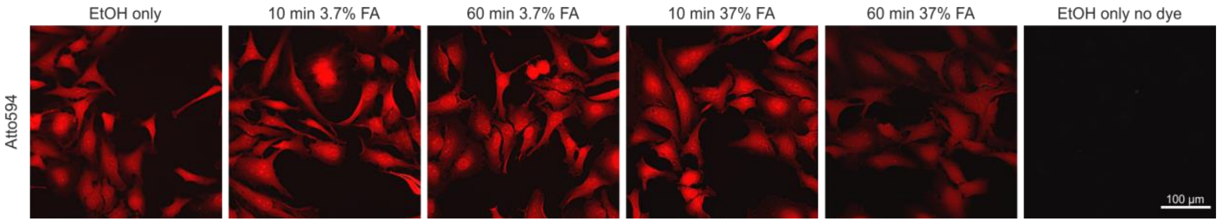

Supplementary Figure 15: Fluorescence microscopy after staining of free lysine residues in HeLa cells with NHS-Atto594 after fixation. *Cells were fixated 24 h after seeding with 70 % ethanol or formaldehyde (FA) as indicated above the images. To ensure similar permeabilization, all samples were treated subsequently with 70 % ethanol for 24 h. Cells were stained with 30 µM NHS-Atto594 for 1 h and subsequently washed three times with PBS followed by fluorescence microscopy. Cells fixated with the same settings as in our isASO-ID experiments (3.7% formaldehyde for 10 min) did not show a decrease in total fluorescence signal indicating high availability of lysine residues for biotinylation. Only cells fixated with 37 % formaldehyde (10-fold increase) for 1 h (6-fold increase) showed a visible decrease of total Atto594 signal compared to the sample treated only with ethanol (N=1).*

Supplementary tables

Supplementary Table 1. *Table of all SILAC-MS/MS experiments performed in this study. Primary data is available on the Pride proteomics database (PXD045992). The number assigned to each experiment (e.g. R16) allows to correlate the data presented in figures and tables with the primary data.*

**Exp. No.**	**Description**		**Comment**	**Figures/ Tables**
BETi				
R16	NLS-Halo-eGFP-BASU, HEK293T Flp-In T-Rex, 10 ng/ml doxycycline, 4 h	L: DMSO<br>M: 500 nM JQ1-CA<br>H: 500 nM JQ1-CA + 5 µM JQ1		1g+h, 3h
R19		L: DMSO<br>M: 500 nM JQ1-CA<br>H: 500 nM JQ1-CA + 5 µM JQ1	Soft wash	
R23		L: 500 nM JQ1-CA + 5 µM JQ1<br>M: DMSO<br>H: 500 nM JQ1-CA		1g+h, 3h
HDACi				
R26	NLS-Halo-eGFP-BASU, HEK293T Flp-In T-Rex, 10 ng/ml doxycycline, 4 h	L: 200 nM Amide-CA<br>M: 200 nM SAHA-CA<br>H: 200 nM SAHA-CA + 1 mM VA		2c, E2c
R27		L: 200 nM SAHA-CA + 1 mM VA<br>M: 200 nM Amide-CA<br>H: 200 nM SAHA-CA		2c+e, E2a+c
R28		L: 200 nM Amide-CA<br>M: 200 nM SAHA-CA<br>H: 200 nM SAHA-CA + 1 mM VA	Soft wash	2d, E2b+d
R29		L: 200 nM SAHA-CA + 1 mM VA<br>M: 200 nM Amide-CA<br>H: 200 nM SAHA-CA	Soft wash	2d, E2d
R30		L: 200 nM Amide-CA<br>M: 200 nM SAHA-CA<br>H: 200 nM SAHA-CA + 2 µM Tacedinaline	Soft wash	S3
HDACi NES				
R54	NES-Halo-eGFP-BASU, HEK293T Flp-In T-Rex, 10 ng/ml doxycycline, 4 h	L: 200 nM Amide-CA<br>M: 200 nM SAHA-CA<br>H: 200 nM SAHA-CA + 1 mM VA		2f
R55		L: 200 nM SAHA-CA + 1 mM VA<br>M: 200 nM Amide-CA		2f

		H: 200 nM SAHA-CA		
high Drug concentration				
R56	NLS-Halo-eGFP-BASU, HEK293T Flp-In T-Rex, 10 ng/ml doxycycline, 24 h	L: DMSO<br>M: 10 µM SAHA-CA<br>H: 10 µM JQ1-CA		2g
R57		L: 10 µM JQ1-CA<br>M: DMSO<br>H: 10 µM SAHA-CA		2g
ASO MOE gapmer 20mer concentration row				
R31	SNAP-eGFP-BASU, HeLa XLone-puro, 500 ng/ml doxycycline, 4 h	L: 10 nM ASO<br>M: 3 nM 3’+5’-BG-ASO mix<br>H: 10 nM 3’+5’-BG-ASO mix		3d, 3f, 3g, E4, S7a+b
R32		L: 100 nM ASO<br>M: 20 nM ASO, 10 nM 3’+5’-BG-ASO mix<br>H: 90 nM ASO, 10 nM 3’+5’-BG-ASO mix		3f, 3g, E4, S7c+d
R37		L: 90 nM ASO, 10 nM 3’+5’-BG-ASO mix<br>M: 100 nM ASO<br>H: 20 nM ASO, 10 nM 3’+5’-BG-ASO mix		3f, 3g, E4, S7c+d
R38		L: 10 nM 3’+5’-BG-ASO mix<br>M: 10 nM ASO<br>H: 3 nM 3’+5’-BG-ASO mix		3d, 3f, 3g, E4, S7a+b
ASO MOE gapmer 20mer 3’ vs 5’ BG comparison				
R21	SNAP-eGFP-BASU, HeLa XLone-puro, 500 ng/ml doxycycline, 4 h	L: 10 nM ASO<br>M: 10 nM 3’-BG-ASO<br>H: 10 nM 5’-BG-ASO		E3c-e
R22		L: 10 nM 5’-BG-ASO<br>M: 10 nM ASO<br>H: 10 nM 3’-BG-ASO		E3c-e
ASO comparison chemistries gapmer MOE vs. LNA				
R39	SNAP-eGFP-BASU, HeLa XLone-puro, 500 ng/ml doxycycline, 4 h	L: Lipofectamine 3000<br>M: 20 nM 16mer MOE-ASO, 10 nM 16mer BG-MOE-ASO<br>H: 20 nM 16mer LNA-ASO, 10 nM 16mer BG-LNA-ASO		E6a-c, E6e
R40		L: Lipofectamine 3000<br>M: 20 nM 20mer MOE-ASO, 10 nM 20mer BG-MOE-ASO<br>H: 20 nM 20mer LNA-ASO, 10 nM 20mer BG-LNA-ASO		4b+c, E6d+e
R42		L: 20 nM 16mer LNA-ASO, 10 nM 16mer BG-LNA-ASO<br>M: Lipofectamine 3000<br>H: 20 nM 16mer MOE-ASO, 10 nM 16mer BG-MOE-ASO		E6a-c, E6e
R43		L: 20 nM 20mer LNA-ASO, 10 nM 20mer BG-LNA-ASO<br>M: Lipofectamine 3000		4b+c E6d+e

		H: 20 nM 20mer MOE-ASO, 10 nM 20mer BG-MOE-ASO	
ASO NLS comparison chemistries gapmer 20mer MOE vs 20mer LNA			
R58	NLS-SNAP-eGFP-BASU, HeLa XLone-puro, 500 ng/ml doxycycline, 4 h	L: Lipofectamine 3000<br>M: 20 nM 20mer MOE-ASO, 10 nM 20mer BG-MOE-ASO<br>H: 20 nM 20mer LNA-ASO, 10 nM 20mer BG-LNA-ASO	E7c-e, S12
R59		L: 20 nM 20mer LNA-ASO, 10 nM 20mer BG-LNA-ASO<br>M: Lipofectamine 3000<br>H: 20 nM 20mer MOE-ASO, 10 nM 20mer BG-MOE-ASO	E7c-e, S12
in situ ASO comparison chemistries gapmer 20mer MOE vs 20mer LNA SNAP-tag			
0971_R09	SNAP-BASU-His	L: Lipofectamine 3000<br>M: 30 nM 20mer BG-MOE-ASO<br>H: 30 nM 20mer BG-LNA-ASO	4h+i, E8a-c
0971_R10		L: 30 nM 20mer BG-LNA-ASO<br>M: Lipofectamine 3000<br>H: 30 nM 20mer BG-MOE-ASO	4h+i, E8a-c
in situ ASO comparison chemistries gapmer 20mer MOE vs 20mer LNA Halo-tag			
0971_R11	Halo-BASU-His	L: Lipofectamine 3000<br>M: 30 nM 20mer CA-MOE-ASO<br>H: 30 nM 20mer CA-LNA-ASO	4f, E8d-f
0971_R12		L: 30 nM 20mer CA-LNA-ASO<br>M: Lipofectamine 3000<br>H: 30 nM 20mer CA-MOE-ASO	4f, E8d-f
in situ MOE gapmer 20mer concentration row			
0971_R27	SNAP-BASU-His	L: 25 nM ASO<br>M: 15 nM 3'+5' BG-ASO mix<br>H: 10 nM ASO, 15 nM 3'+5' BG-ASO mix	5b, E9a-c, E9g
0971_R28		L: 10 nM ASO, 15 nM 3'+5' BG-ASO mix<br>M: 25 nM ASO<br>H: 15 nM 3'+5' BG-ASO mix	5b, E9a-c, E9g
0971_R29		L: 100 nM ASO<br>M: 35 nM ASO, 15 nM 3'+5' BG-ASO mix<br>H: 85 nM ASO, 15 nM 3'+5' BG-ASO mix	5b, E9d-g
0971_R30		L: 85 nM ASO, 15 nM 3'+5' BG-ASO mix<br>M: 100 nM ASO<br>H: 35 nM ASO, 15 nM 3'+5' BG-ASO mix	5b, E9d-g
in situ MOE gapmer 20mer Actinomycin D treatment			
0971_R31	SNAP-BASU-His	L: 15 nM ASO, 1.5 µg/ml ActD, 1 h<br>M: 15 nM 3'+5' BG-ASO mix<br>H: 15 nM 3'+5' BG-ASO mix, 1.5 µg/ml ActD, 1 h	5d+e, S13
0971_R32		L: 15 nM 3'+5' BG-ASO, 1.5 µg/ml ActD, 1 h<br>M: 15 nM ASO, 1.5 µg/ml ActD, 1 h	5d+e, S13

		H: 15 nM 3'+5' BG-ASO mix	
in situ ASO comparison chemistries gapmer 20mer MOE vs 20mer Fluoro SNAP-tag			
0971_R59	SNAP-BASU-His	L: Lipofectamine 3000<br>M: 15 nM 20mer BG-MOE-ASO<br>H: 15 nM 20mer BG-F-ASO	S14b-j
0971_R60		L: 15 nM 20mer BG-F-ASO<br>M: Lipofectamine 3000<br>H: 15 nM 20mer BG-MOE-ASO	S14b-j
0971_R61		L: Lipofectamine 3000<br>M: 35 nM ASO + 15 nM 20mer BG-MOE-ASO<br>H: 35 nM ASO + 15 nM 20mer BG-F-ASO	S14b-j
0971_R62		L: 35 nM ASO + 15 nM 20mer BG-F-ASO<br>M: Lipofectamine 3000<br>H: 35 nM ASO + 15 nM 20mer BG-MOE-ASO	S14b-j

Supplementary Table 2. *Antibodies used in this study*

Antibody, Target Protein	Produced in/ Immunoglo-bulin class	Dilution	Supplier, Order Nr.	Epitope	Validation
α-SNAP	rabbit, polyclonal, affinity purified	1:2000	New England Bioloabs, P9310S	Immunization with purified recombinant SNAP-tag protein	Validated in our laboratory via overexpression of SNAP-tag fusion proteins
α-Halo	rabbit, polyclonal	1:1000	Promega, G9281	n.a.	Validated in our laboratory via overexpression of Halo-tag fusion proteins
α-eGFP, green fluorescent protein	rabbit, polyclonal, affinity purified	1:2000	Sigma Aldrich, g1544	Synthetic peptide corresponding amino acids 3-17 of GFP	Validated in our laboratory via overexpression of GFP-tag fusion proteins
α-PC, pyruvate carboxylase	rabbit, polyclonal, affinity purified	1:1000	Sigma Aldrich, HPA043922	GADVVDVAADSMSGMTSQP SMGALVACTRGTPLDTEVPM ERVFDYSEYWEGARGLYAAF DCTATMKSGNSDVYENEIPG GQYTNLHFQ	PMID: 26302408
α-ACTB, ß-actin	mouse, monoclonal IgG Clone AC-15	1:3000	Sigma Aldrich, A5441	Actin N-terminal peptide, Ac-DDDIAALVIDANGSGK	PMID: 30814728
α-GAPDH, glyceraldehyde 3-phosphate dehydrogenase	mouse, monoclonal IgG	1:2000-10,000	Thermo Scientific, GA1R	Recombinant GAPDH	PMID: 31519936
α-HDAC6, histone deacetylase 1	rabbit, polyclonal IgG	1:500	Santa Cruz Biotechnology, H-300, sc-11420	Amino acids 916-1215 of HDAC6 of human HDAC6	PMID: 31270913
α-HDAC1, histone deacetylase 1	mouse, monoclonal IgG	1:500	Santa Cruz Biotechnology, sc-81698	C-terminus (amino acids 432-482) of human HDAC1	PMID: 31270913
α-HDAC3, histone deacetylase 1	rabbit, polyclonal IgG	1:500	Santa Cruz Biotechnology H-99, sc-11417,	Amino acids 330-428 of human HDAC3	PMID: 31270913
α-KDM1, lysine-specific demethylase 1	rabbit, serum	1:1000	Wolfram Antonin laboratory, FMI, Tuebingen, Germany	Full length Xenopus KDM1(LSD1) expressed from pET28a	PMID: 26224877
α-NONO, p54/nrb	mouse, monoclonal IgG	1:200	Santa Cruz Biotechnology sc-376865	Amino acids 381-465 of human p54/nrb	PMID: 29165591
α-mouse	goat, polyclonal, IgG, HRP conjugate	1:5000	Jackson Immuno Research, 115-035-003	Whole mouse IgG	
α-rabbit	goat, polyclonal, IgG, HRP conjugate	1:5000	Jackson Immuno Research, 111-035-003	Whole rabbit IgG	
α-mouse-AlexaFluor594	goat, polyclonal, IgG, Alexa Fluor 594 conjugate	1:2000	ThermoFisher Scientific, A-11005	Whole mouse IgG	

Supplementary Table 3. *Sequences and ε260 nm of used ASOs. 2'-MOE modified nucleotides are shown in italic, LNA modified nucleotides in bold, 2'-Fluoro modified nucleotides are indicated by a prefixed f, and the DNA gap is underlined. Phosphorothioate linkages have been marked with a star (*). Control ASO does not contain any terminal modifications. 3'-modifications have been attached via a 7 carbon atom linker at the 3'-phosphate and 5'-modifications have been attached via a 6 carbon atom linker at the 5'-phosphate.*

Oligo	Sequence	$\varepsilon_{260\ nm}$ ($\varepsilon_{603\ nm}$ or $\varepsilon_{500\ nm}$) $mm^{-1}cm^{-1}$	Used in figure
Control ASO 20mer MOE	5'- mC*T*G*mC*T*A*G*C*C*T*C*T*G*G*A*T*T*T*G*A -3'	187.8	3c-g, 3i, 4a-c, 5, E3a-d, E4, E9a+b, E9d+e, E9g, E10, S6b, S7, S8, S9
3'NH2-ASO 20mer MOE	5'-mC*T*G*mC*T*A*G*C*C*T*C*T*G*G*A*T*T*T*G*A- C7-NH2-3'	187.8	E3b, S9
5'NH2-ASO 20mer MOE	5'-NH2-C6- mC*T*G*mC*T*A*G*C*C*T*C*T*G*G*A*T*T*T*G*A -3'	187.8	E3b, E5b, E6d-e, E7a-e, E8a-f, S9, S12, S13a
3'BG-ASO 20mer MOE	5'- mC*T*G*mC*T*A*G*C*C*T*C*T*G*G*A*T*T*T*G*A -C7-BG-3'	190.3	3c-g, 5, E3a+b, E3d+e, E4, E9, E10, S6b, S7, S8, S9, S13b
5'BG-ASO 20mer MOE	5'-BG-C6-mC*T*G*mC*T*A*G*C*C*T*C*T*G*G*A*T*T*T*G*A-3'	190.3	3c-g, 4a-c, 4g-i, 5, E3a-c, E3e, E4, E5b, E6d+e, E7a-c, E7e, E8a+c, E9, E10, S6b, S7, S8, S9, S10, S12, S13
5'CA-ASO 20mer MOE	5'-CA-C6-mC*T*G*mC*T*A*G*C*C*T*C*T*G*G*A*T*T*T*G*A-3'	190.3	4d-f, E8d+f
3'Atto594-ASO 20mer MOE	5'- mC*T*G*mC*T*A*G*C*C*T*C*T*G*G*A*T*T*T*G*A - C7-Atto594-3'	214.2 (120)	3e, 3a, S8
5'Atto594-ASO 20mer MOE	5'-Atto594-C6-mC*T*G*mC*T*A*G*C*C*T*C*T*G*G*A*T*T*T*G*A -3'	214.2 (120)	3e, 3a, E5b, E7a, S8
5'Atto488-ASO 20mer MOE	5'-Atto488-C6-mC*T*G*mC*T*A*G*C*C*T*C*T*G*G*A*T*T*T*G*A -3'	207.6 (90)	4e, 4g, 5a, 5c
5'NH2-ASO 16mer MOE	5'-NH2-C6- G*mC*T*A*G*C*C*T*C*T*G*G*A*T*T*T -3'	155.0	E6a, E6c, E6e, S9a
5'BG-ASO 16mer MOE	5'-BG-C6- G*mC*T*A*G*C*C*T*C*T*G*G*A*T*T*T -3'	157.5	E6a, E6c, E6e, S9a, S10
5'Atto594-ASO 16mer MOE	5'-Atto594-C6- G*mC*T*A*G*C*C*T*C*T*G*G*A*T*T*T -3'	181.4 (120)	S11a
5'NH2-ASO 20mer LNA	5'-NH2-C6- mC*T*G*mC*T*A*G*C*C*T*C*T*G*G*A*T*T*T*G*A -3'	185.0	E5a, E6e, S8a
5'BG-ASO 20mer LNA	5'-BG-C6- mC*T*G*mC*T*A*G*C*C*T*C*T*G*G*A*T*T*T*G*A -3'	187.5	4g-i, E5a, E6e, S10, S13a
5'CA-ASO 20mer LNA	5'-CA-C6- mC*T*G*mC*T*A*G*C*C*T*C*T*G*G*A*T*T*T*G*A -3'	187.5	4e+f, E8e+f
5'Atto594-ASO 20mer LNA	5'-Atto594-C6-mC*T*G*mC*T*A*G*C*C*T*C*T*G*G*A*T*T*T*G*A -3'	211.4 (120)	4a, E5a

5'Atto488-ASO 20mer LNA	5’-Atto488-C6- **mC*T*G*mC*T***A*G*C*C*T*C*T*G*G*A***T*T*T*G*A** -3’	204.8 (90)	4e, 4g
5'NH2-ASO 16mer LNA	5’-NH2-C6- **G*mC*T***A*G*C*C*T*C*T*G*G*A***T*T*T** -3’	148.0	E6b+c, E6e, S9b
5'BG-ASO 16mer LNA	5’-BG-C6- **G*mC*T***A*G*C*C*T*C*T*G*G*A***T*T*T** -3’	150.5	E6b+c, E6e, S10, S11b
5'Atto594-ASO 16mer LNA	5’-Atto594-C6- **G*mC*T***A*G*C*C*T*C*T*G*G*A***T*T*T** -3’	174.4 (120)	S11b
5'NH2-ASO 20mer F	5’-NH2-C6- f**C*fU*fG*fC*fU***A*G*C*C*T*C*T*G*G*A***fU*fU*fU*fG*fA** -3’	195.0	S14
5'BG-ASO 20mer F	5’-BG-C6- f**C*fU*fG*fC*fU***A*G*C*C*T*C*T*G*G*A***fU*fU*fU*fG*fA** -3’	197.5	S14
5'Atto488-ASO 20mer F	5’-Atto488-C6- f**C*fU*fG*fC*fU***A*G*C*C*T*C*T*G*G*A***fU*fU*fU*fG*fA** -3’	213.0 (90)	S14a

Supplementary Note 1: Chemical synthesis

Chemicals

If not stated otherwise, all substrates and reagents required for synthesis and biochemical studies were purchased from commercial providers and used without further purification. JQ1 (CAS 1268524-70-4, BLDpharm) and Tacedinaline (CI-994, CAS 112522-64-2, Sigma Aldrich) were obtained commercially and used without further purification.

General Methods

All column chromatographic purifications were carried out on self-packed columns of silica gel (0.04-0.063 mm/230-240 mesh). Thin-layer chromatography (TLC) was performed on silica gel sheets (60 F254, 0.2 mm, 5 x 10 cm, Merck) and visualized under UV light (254 nm). All analytical and preparative HPLC runs were performed on a Shimadzu system (SCL-10A VP, SPD-20AV, LC-20AT) running with 0.1% TFA in water (Eluent A) and 0.1% TFA in acetonitrile/water (9:1, Eluent B). Analytical HPLC was performed using an EC 125/4 Nucleodur C18 column by Machery + Nagel and preparative HPLC was performed using a VP 250/10 Nucleodur C18 column by Machery + Nagel. High resolution mass spectrometry was performed on a maXis4G ESI-TOF-MS by Bruker Daltonics.

Synthesis of JQ-CA

2-(2-((6-Chlorohexyl)oxy)ethoxy)ethanamine (**4**, NH2-CA) was synthesized from commercially available 2-(2-aminoethoxy)ethanol according to literature (Singh, V., Wang, S. & Kool, E.T. Genetically Encoded Multispectral Labeling of Proteins with Polyfluorophores on a DNA Backbone. JACS 135 (16), 6184-6191, 2013).

Synthesis Scheme for JQ-CA

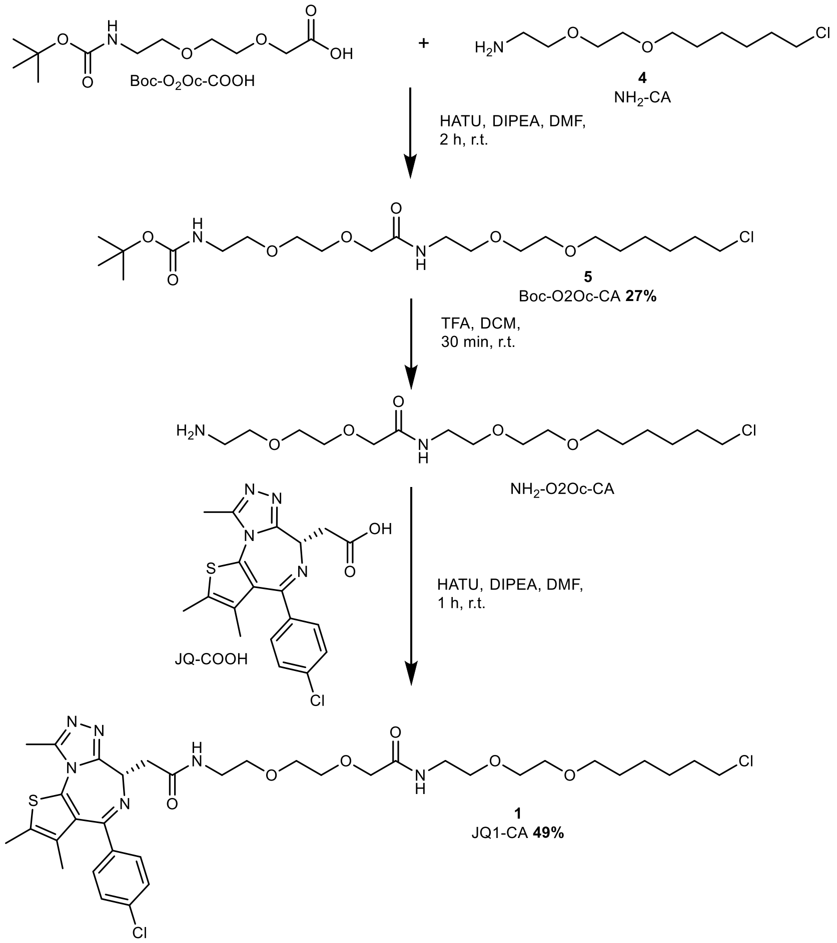

Synthesis of tert-butyl (21-chloro-8-oxo-3,6,12,15-tetraoxa-9-azahenicosyl) carbamate (Boc-O2Oc-CA, 2)

8-(t-Butyloxycarbonyl-amino)-3,6-dioxaoctanoic acid dicyclohexylammonium salt (Boc-O2Oc-COOH*DCHA, Iris Biotech, 1 g) was solved in DCM and washed three times with 1 M aqueous HCl. The solution was dried over sodium sulfate and then filtered. The solvent was removed under vacuum resulting in a colorless oil which was used for the synthesis.

Boc-O2Oc-COOH (263.29 g/mol, 39 mg, 0.145 mmol, 2.25 eq) and DIPEA (129.25 g/mol, 0.742 g/ml, 27 µl, 39.4 mg, 0.157 mmol, 5.0 eq) were dissolved in DMF (1 ml) and cooled to 0 °C. HATU (379.25 g/mol, 48.5 mg, 0.128 mmol, 2.0 eq) was dissolved in 1 ml DMF, added dropwise and the reaction was allowed to warm to room temperature.

NH2-CA (**4**, 223.74 g/mol, 15 mg, 0.064 mmol, 1.0 eq) and DIPEA (129.25 g/mol, 0.742 g/ml, 27 µl, 39.4 mg, 0.157 mmol, 5.0 eq) were dissolved in DMF (0.5 ml) and added dropwise. The reaction was stirred for 2 h before quenching with 200 µl water and DMF was removed on the rotary evaporator. Water (10 ml) was added and the solution was extracted with dichloromethane (3x10 ml). The organic phase was washed two times with brine (5 ml), dried over sodium sulfate, and evaporated under reduced pressure. The crude product was purified using preparative HPLC yielding in 8.6 mg (27%) of a colorless oil. m/z calculated for $[C_{21}H_{41}ClN_2O_7+Na]^+$: **491.24945**, found: **491.25029**

Synthesis of (S)-N-(21-chloro-8-oxo-3,6,12,15-tetraoxa-9-azahenicosyl)-2-(4-(4-chlorophenyl)-2,3,9-trimethyl-6H-thieno[3,2-f][1,2,4]triazolo[4,3-a][1,4]diazepin-6-yl)acetamide (JQ-CA, 1)

Boc-O2Oc-CA (**5**, 469 g/mol, 6.5 mg, 0.014 mmol, 1.1 eq) was dissolved in DCM (2 ml) and cooled to 0 °C, followed by the addition of TFA (114.02 g/mol, 22.3 mg, 15 µl, 0.196 mmol, 15.4 eq). The reaction was allowed to warm to room temperature and was stirred for 30 min. The solvent was removed on a rotary evaporator. The deprotected NH2-O2OC-CA was dried under high vacuum and used for the reaction without further purification.

JQ1-carboxylic acid (CAS 202592-23-2, BLDpharm, 400.88 g/mol, 5 mg, 0.013 mmol, 1.0 eq) and DIPEA (129.25 g/mol, 0.742 g/ml, 11 µl, 8.4 mg, 0.65 mmol, 5.0 eq) were dissolved in DMF (0.5 ml) and cooled to 0 °C, followed by the dropwise addition of HATU (379.25 g/mol, 5.5 mg, 0.014 mmol, 1.1 eq, in 0.25 ml DMF). The reaction was allowed to warm to room temperature. NH2-O2Oc-CA (368.9g/mol, 5.2 mg, 0.014 mmol, 1.1 eq, generated *in situ*) and DIPEA (129.25 g/mol, 0.742 g/ml, 11 µl, 8.4 mg, 0.65 mmol, 5.0 eq) were dissolved in DMF (0.5 ml) and added dropwise. The reaction was stirred for 1 h, quenched with 200 µl water and the solvent was removed under vacuum. The crude product was directly applied to preparative HPLC obtaining 4.6 mg (49%) white powder. m/z calculated for $[C_{35}H_{48}Cl_2N_6O_6S+Na]^+$: **773.26253**, found: **773.26241**

Synthesis of SAHA-CA and Amide-CA

Synthesis of 1-(4-(8-(hydroxyamino)-8-oxooctanamido)phenyl)-3-oxo-4,7,10-trioxa-2-azadodecan-12-yl (2-(2-((6-chlorohexyl)oxy)ethoxy)ethyl)carbamate (**2**, SAHA-CA) was carried out as described by Friedmann Ohana et al. (Deciphering the Cellular Targets of Bioactive Compounds Using a Chloroalkane Capture Tag. ACS Chem. Biol. 10, 2316–2324, 2015). The molecular formula was confirmed by HR-MS (m/z calculated for $[C_{33}H_{55}ClN_4O_{11}+Na]^+$: **741.34481**, found: **741.34535**).

Synthesis of 1-(4-(8-amino-8-oxooctanamido)phenyl)-3-oxo-4,7,10-trioxa-2-azado-decan-12-yl (2-(2-((6-chlorohexyl)oxy)ethoxy)ethyl)carbamate **(3, Amide-CA)** was carried out following a similar synthesis strategy starting from methyl 8-[(4-{[(tert-butoxycarbonyl)amino]methyl}phenyl)amino]-8-oxooctanoate **(6)** and 2-(2-(2-(((4-nitrophenoxy)carbonyl)oxy)ethoxy)ethoxy)ethyl(2-(2-((6-chlorohexyl)oxy)ethoxy) ethyl) carbamate **(7). 6** and **7** were synthesized from commercially available chemicals according to literature (Los, G.V. et al. HaloTag: A Novel Protein Labeling Technology for Cell Imaging and Protein Analysis. ACS Chem. Biol. 3, 373–382, 2008 and Kral, A.M. et al. Divergent Kinetics Differentiate the Mechanism of Action of Two HDAC Inhibitors. Biochemistry 53 (4), 725-734, 2014).

Synthesis scheme for Amide-CA

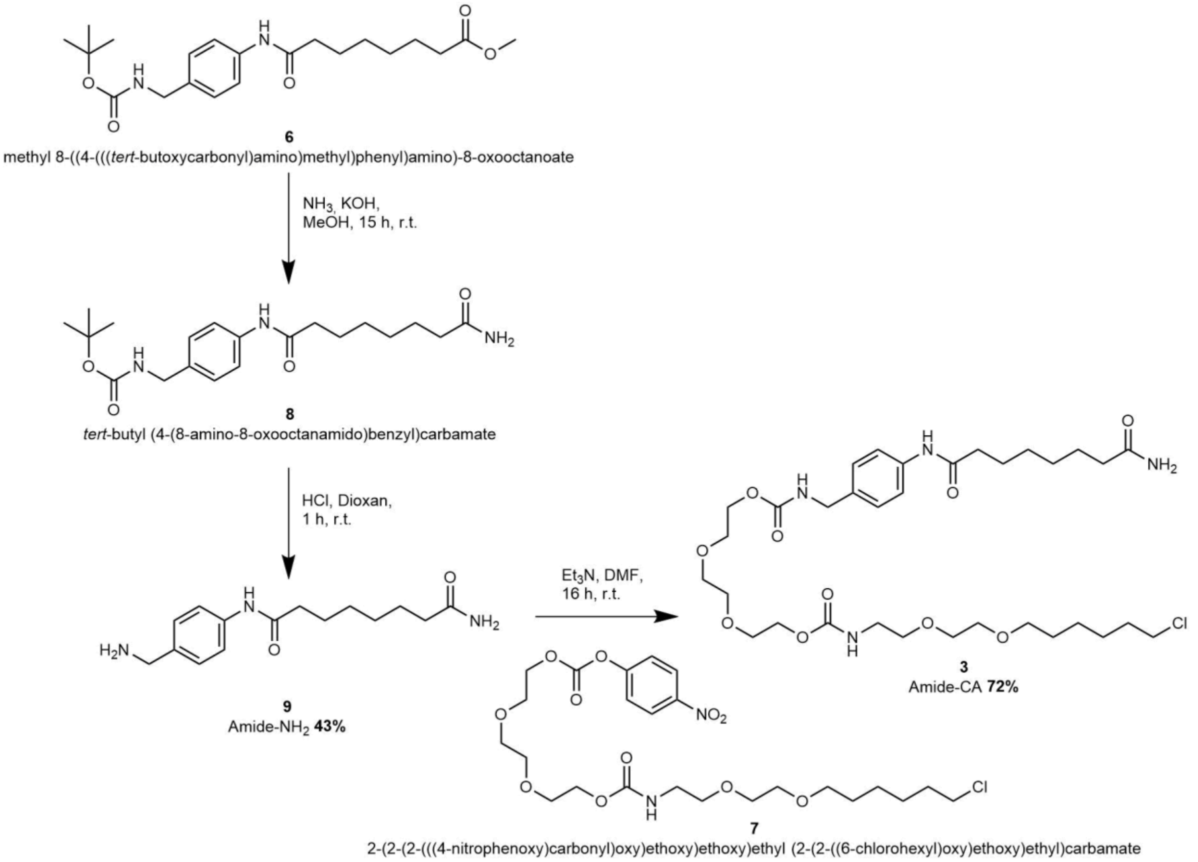

Synthesis of tert-butyl (4-(8-amino-8-oxooctanamido)benzyl)carbamate (8)
6 (100 mg, 0.25 mmol, 1 eq) was solved in ammonia/methanol (6.7 ml, 7 N, 46.9 mmol, 188 eq) and aq 5 M potassium hydroxide (0.36 ml, 1.81 mmol, 7.1 eq). The resulting solution was stirred for 15 h. The reaction mixture was quenched in 45 ml water and extracted twice with DCM. The organic layer was concentrated by rotary evaporation and used without further purification.

Synthesis of N1-(4-(aminomethyl)phenyl)octanediamide (Amide-NH2 , 9)

Tert-butyl (4-(8-amino-8-oxooctanamido)benzyl)carbamate (**8**, 52 mg, 0.14 mmol, 1 eq) was stirred in 2 ml 1,4-dioxane. 4M HCl/1,4-dioxane (1 ml, 4.14 mmol, 30 eq) was added dropwise and the reaction mixture was stirred for 1 h, concentrated *in vacuo* and purified by preparative HPLC. Product fractions were identified by LC-MS, combined and lyophilized to yield 16.5 mg (59.5 mol, 43.2%) of product. m/z calculated for $[C_{15}H_{23}N_3O_2+Na]^+$: **300.16825**, found: **300.16870**

Synthesis of 1-(4-(8-amino-8-oxooctanamido)phenyl)-3-oxo-4,7,10-trioxa-2-azadodecan-12-yl (2-(2-((6-chlorohexyl)oxy)ethoxy)ethyl)carbamate (Amide-CA, 3)
To **9** (14.7 mg, 53 µmol, 1.2 eq) a solution of **7** (25.0 mg, 44 µmol, 1 eq) in 1 ml anhydrous DMF was added. Upon addition of triethylamine (25 µl, 180 µmol, 4 eq) the mixture turned instantly yellow and the reaction was stirred overnight. DMF and triethylamine were removed under vacuum and the crude product was directly applied to preparative HPLC yielding 22.5 mg (32 µmol, 72 %) pure product. m/z calculated for $[C_{33}H_{55}ClN_4O_{10}+Na]^+$: **725.34989**, found: **725.35104**

Synthesis of BG- and CA-linker-OH

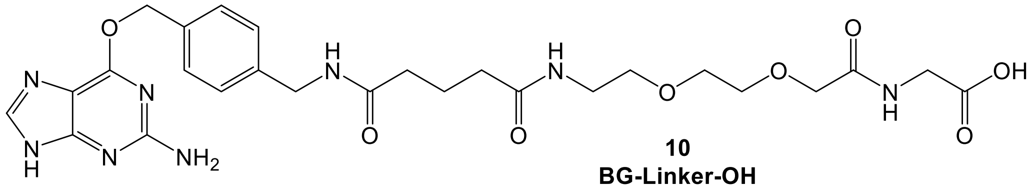

10
BG-Linker-OH
Molecular Weight: 586,61

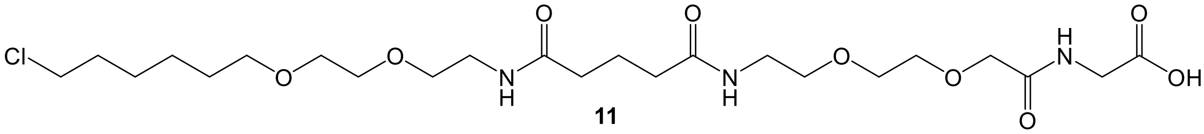

11
CA-Linker-OH
Molecular Weight: 540,05

Synthesis of BG-linker-OH

Preloaded H-Gly-Chlorotrityl resin (178 mg, 0.63 mmol), 111 µmol, 1 eq) is swelled twice for 10 min in 5 ml NMP. Fmoc-PEG (170.9 mg, 444 µmol) 8 eq), HBTU (151.4 mg, 400 µmol, 7.2 eq) and HOBT (59.9 mg, 444 µmol, 8 eq) are dissolved in 2 ml NMP. DIPEA (543 µl, 3.11 mmol, 56 eq) is added and shook for 15 min before loading on the resin. The reaction is incubated for 1 h on a peptide shaker and washed 3x each with DCM/NMP (1:1), DCM and NMP. Free NH2-groups are captured by incubating two times with 6 ml DIPEA + acetic anhydride in NMP (1:1:10) and washing of the resin 4x each with DCM/NMP (1:1), DCM and NMP. Fmoc-PEG is deprotected by adding 3x piperidine (20% in NMP) for 10 min each while shaking. Resin is washed 3x each with DCM/NMP (1:1), DCM and NMP. Glutaric anhydride is dissolved in 1.2 ml NMP + 200 µl DIPEA, loaded onto resin and incubated for 30 min while shaking. Resin is washed again 3x each with DCM/NMP (1:1), DCM and NMP and additionally an aquatic wash with Dioxan/water (1:1) in 1% NaOH is performed for 1 min. Resin is washed another 3x each with DCM/NMP (1:1), DCM and NMP. Preactivation is performed with PFP-TFA (200 µl, 1.17 mmol, 21 eq) in 5ml pyridine/DCM (1:1) by loading it onto resin for 30 min followed by washing 4x with NMP. BG-NH2 is dissolved in 3 ml pyridine/DMSO (1:20) and incubated over night while shaking. Resin is washed 4x each with DCM/NMP (1:1), DCM, NMP and diethylether. For cleaving, resin is incubated 2x for 5 min each with NMP, washed 5x with DCM and 5x 5 ml 20% HFIP/DCM are pushed though the syringe to release the linker. The linker can be further purified by preparative HPLC if necessary.

Synthesis of CA-linker-OH

Synthesis of CA-linker-OH is carried out analogously to BG-linker-OH. Instead of BG-NH2, commercially available 2-(2-((6-chlorohexyl)oxy)ethoxy)ethan-1-amine is used

in the last coupling step. For synthesis of 2-(2-((6-chlorohexyl)oxy)ethoxy)ethan-1-amine, please refer to ref 53.

Supplementary Note 2: DNA sequences of inserts and plasmids

NLS-Halo-eGFP-BASU

ATGGGACCAAAAAAGAAAAGAAAAGTTGCTCCAGGCTCAGGTGCAGAAATCGGTACTGGCTTTCCATT
CGACCCCCATTATGTGGAAGTCCTGGGCGAGCGCATGCACTACGTCGATGTTGGTCCGCGCGATGGCA
CCCCTGTGCTGTTCCTGCACGGTAACCCGACCTCCTCCTACGTGTGGCGCAACATCATCCCGCATGTT
GCACCGACCCATCGCTGCATTGCTCCAGACCTGATCGGTATGGGCAAATCCGACAAACCAGACCTGGG
TTATTTCTTCGACGACCACGTCCGCTTCATGGATGCCTTCATCGAAGCCCTGGGTCTGGAAGAGGTCG
TCCTGGTCATTCACGACTGGGGCTCCGCTCTGGGTTTCCACTGGGCCAAGCGCAATCCAGAGCGCGTC
AAAGGTATTGCATTTATGGAGTTCATCCGCCCTATCCCGACCTGGGACGAATGGCCAGAATTTGCCCG
CGAGACCTTCCAGGCCTTCCGCACCACCGACGTCGGCCGCAAGCTGATCATCGATCAGAACGTTTTTA
TCGAGGGTACGCTGCCGATGGGTGTCGTCCGCCCGCTGACTGAAGTCGAGATGGACCATTACCGCGAG
CCGTTCCTGAATCCTGTTGACCGCGAGCCACTGTGGCGCTTCCCAAACGAGCTGCCAATCGCCGGTGA
GCCAGCGAACATCGTCGCGCTGGTCGAAGAATACATGGACTGGCTGCACCAGTCCCCTGTCCCGAAGC
TGCTGTTCTGGGGCACCCCAGGCGTTCTGATCCCACCGGCCGAAGCCGCTCGCCTGGCCAAAAGCCTG
CCTAACTGCAAGGCTGTGGACATCGGCCCGGGTCTGAATCTGCTGCAAGAAGACAACCCGGACCTGAT
CGGCAGCGAGATCGCGCGCTGGCTGTCGACGCTCGAGAAGCCAACCCCAGCAGGCGGAGGCGCGCCAG
GGTCTGGCGTGAGCAAGGGCGAGGAGCTGTTCACCGGGGTGGTGCCCATCCTGGTCGAGCTGGACGGC
GACGTAAACGGCCACAAGTTCAGCGTGTCCGGCGAGGGCGAGGGCGATGCCACCTACGGCAAGCTGAC
CCTGAAGTTCATCTGCACCACCGGCAAGCTGCCCGTGCCCTGGCCCACCCTCGTGACCACCCTGACCT
ACGGCGTGCAGTGCTTCAGCCGCTACCCCGACCACATGAAGCAGCACGACTTCTTCAAGTCCGCCATG
CCCGAAGGCTACGTCCAGGAGCGCACCATCTTCTTCAAGGACGACGGCAACTACAAGACCCGCGCCGA
GGTGAAGTTCGAGGGCGACACCCTGGTGAACCGCATCGAGCTGAAGGGCATCGACTTCAAGGAGGACG
GCAACATCCTGGGGCACAAGCTGGAGTACAACTACAACAGCCACAACGTCTATATCATGGCCGACAAG
CAGAAGAACGGCATCAAGGTGAACTTCAAGATCCGCCACAACATCGAGGACGGCAGCGTGCAGCTCGC
CGACCACTACCAGCAGAACACCCCCATCGGCGACGGCCCCGTGCTGCTGCCCGACAACCACTACCTGA
GCACCCAGTCCGCCCTGAGCAAAGACCCCAACGAGAAGCGCGATCACATGGTCCTGCTGGAGTTCGTG
ACCGCCGCCGGGATCACTCTCGGCATGGACGAGCTGTACAAGCCGGTCACCGGCGGAAAACTTAGCGA
GAGCGAGATCCGCTTCGGCCTGAAAACCGAAGTGATGGGCCAGCACCTGATCTACCACGACGTGCTGA
GCAGCACCCAGAAAACAGCCCACGAGCTGGCCAACAACAATGCCCCTGAGGGAACACTGGTGGTGGCC
GATAAGCAGACAGCTGGCCGCGGCGGAATGAGCCGCGTGTGGCACAGCCAAGAAGGCAACGGCGTCTG
GATGAGCCTGATCCTGCGCCCTGACATCCCTCTCCAGAAAACCCCTCAGCTGACACTGCTGGCTGCTG
TGGCTGTGGTGCAGGGAATTGAAGAAGCCGCCGGAATCCAGACCGACATCAAGTGGCCCAACGACATC
CTGATCAACGGCAAGAAAACCGTGGGCATCCTGACCGAGATGCAGGCCGAAGAGGACAGAGTCCGCAG
CGTGATCATCGGCATCGGGATCAATGTGAACCAGCAGCCTAACGACTTCCCCGACGAGCTGAAGGATA
TCGCCACAAGCCTGTCTCAGGCCGCTGGCGAGAAGATTGATCGCGCTGGCGTGATCCAGCACATCCTG
CTGTGCTTCGAGAAGCGGTACCGCGACTACATGACCCACGGCTTCACCCCTATCAAACTGCTGTGGGA
GAGCTACGCCCTCGGAATCGGCACCAACATGCGCGCCCGCACACTGAACGGCACCTTCTACGGCAAGG
CCCTGGGAATTGATGACGAGGGCGTTCTGCTGCTGGAAACAAACGAGGGCATCAAGAAAATCTACAGC
GCCGACATCAGCCTGCGCTAA

NES-Halo-eGFP-BASU

CTGCAGAAAAAGCTGGAAGAGTTGGAACTGGCTCCAGGCTCAGGTGCAGAAATCGGTACTGGCTTTCC
ATTCGACCCCCATTATGTGGAAGTCCTGGGCGAGCGCATGCACTACGTCGATGTTGGTCCGCGCGATG
GCACCCCTGTGCTGTTCCTGCACGGTAACCCGACCTCCTCCTACGTGTGGCGCAACATCATCCCGCAT
GTTGCACCGACCCATCGCTGCATTGCTCCAGACCTGATCGGTATGGGCAAATCCGACAAACCAGACCT
GGGTTATTTCTTCGACGACCACGTCCGCTTCATGGATGCCTTCATCGAAGCCCTGGGTCTGGAAGAGG
TCGTCCTGGTCATTCACGACTGGGGCTCCGCTCTGGGTTTCCACTGGGCCAAGCGCAATCCAGAGCGC
GTCAAAGGTATTGCATTTATGGAGTTCATCCGCCCTATCCCGACCTGGGACGAATGGCCAGAATTTGC
CCGCGAGACCTTCCAGGCCTTCCGCACCACCGACGTCGGCCGCAAGCTGATCATCGATCAGAACGTTT
TTATCGAGGGTACGCTGCCGATGGGTGTCGTCCGCCCGCTGACTGAAGTCGAGATGGACCATTACCGC
GAGCCGTTCCTGAATCCTGTTGACCGCGAGCCACTGTGGCGCTTCCCAAACGAGCTGCCAATCGCCGG
TGAGCCAGCGAACATCGTCGCGCTGGTCGAAGAATACATGGACTGGCTGCACCAGTCCCCTGTCCCGA
AGCTGCTGTTCTGGGGCACCCCAGGCGTTCTGATCCCACCGGCCGAAGCCGCTCGCCTGGCCAAAAGC

CTGCCTAACTGCAAGGCTGTGGACATCGGCCCGGGTCTGAATCTGCTGCAAGAAGACAACCCGGACCT
GATCGGCAGCGAGATCGCGCGCTGGCTGTCGACGCTCGAGAAGCCAACCCCAGCAGGCGGAGGCGCGC
CAGGGTCTGGCGTGAGCAAGGGCGAGGAGCTGTTCACCGGGGTGGTGCCCATCCTGGTCGAGCTGGAC
GGCGACGTAAACGGCCACAAGTTCAGCGTGTCCGGCGAGGGCGAGGGCGATGCCACCTACGGCAAGCT
GACCCTGAAGTTCATCTGCACCACCGGCAAGCTGCCCGTGCCCTGGCCCACCCTCGTGACCACCCTGA
CCTACGGCGTGCAGTGCTTCAGCCGCTACCCCGACCACATGAAGCAGCACGACTTCTTCAAGTCCGCC
ATGCCCGAAGGCTACGTCCAGGAGCGCACCATCTTCTTCAAGGACGACGGCAACTACAAGACCCGCGC
CGAGGTGAAGTTCGAGGGCGACACCCTGGTGAACCGCATCGAGCTGAAGGGCATCGACTTCAAGGAGG
ACGGCAACATCCTGGGGCACAAGCTGGAGTACAACTACAACAGCCACAACGTCTATATCATGGCCGAC
AAGCAGAAGAACGGCATCAAGGTGAACTTCAAGATCCGCCACAACATCGAGGACGGCAGCGTGCAGCT
CGCCGACCACTACCAGCAGAACACCCCCATCGGCGACGGCCCCGTGCTGCTGCCCGACAACCACTACC
TGAGCACCCAGTCCGCCCTGAGCAAAGACCCCAACGAGAAGCGCGATCACATGGTCCTGCTGGAGTTC
GTGACCGCCGCCGGGATCACTCTCGGCATGGACGAGCTGTACAAGCCGGTCACCGGCGGAAAACTTAG
CGAGAGCGAGATCCGCTTCGGCCTGAAAACCGAAGTGATGGGCCAGCACCTGATCTACCACGACGTGC
TGAGCAGCACCCAGAAAACAGCCCACGAGCTGGCCAACAACAATGCCCCTGAGGGAACACTGGTGGTG
GCCGATAAGCAGACAGCTGGCCGCGGCGGAATGAGCCGCGTGTGGCACAGCCAAGAAGGCAACGGCGT
CTGGATGAGCCTGATCCTGCGCCCTGACATCCCTCTCCAGAAAACCCCTCAGCTGACACTGCTGGCTG
CTGTGGCTGTGGTGCAGGGAATTGAAGAAGCCGCCGGAATCCAGACCGACATCAAGTGGCCCAACGAC
ATCCTGATCAACGGCAAGAAAACCGTGGGCATCCTGACCGAGATGCAGGCCGAAGAGGACAGAGTCCG
CAGCGTGATCATCGGCATCGGGATCAATGTGAACCAGCAGCCTAACGACTTCCCCGACGAGCTGAAGG
ATATCGCCACAAGCCTGTCTCAGGCCGCTGGCGAGAAGATTGATCGCGCTGGCGTGATCCAGCACATC
CTGCTGTGCTTCGAGAAGCGGTACCGCGACTACATGACCCACGGCTTCACCCCTATCAAACTGCTGTG
GGAGAGCTACGCCCTCGGAATCGGCACCAACATGCGCGCCCGCACACTGAACGGCACCTTCTACGGCA
AGGCCCTGGGAATTGATGACGAGGGCGTTCTGCTGCTGGAAACAAACGAGGGCATCAAGAAAATCTAC
AGCGCCGACATCAGCCTGCGCTAA

NLS-Halo-eGFP-BioID

ATGGGACCAAAAAAGAAAAGAAAAGTTGCTCCAGGCTCAGGTGCAGAAATCGGTACTGGCTTTCCATT
CGACCCCCATTATGTGGAAGTCCTGGGCGAGCGCATGCACTACGTCGATGTTGGTCCGCGCGATGGCA
CCCCTGTGCTGTTCCTGCACGGTAACCCGACCTCCTCCTACGTGTGGCGCAACATCATCCCGCATGTT
GCACCGACCCATCGCTGCATTGCTCCAGACCTGATCGGTATGGGCAAATCCGACAAACCAGACCTGGG
TTATTTCTTCGACGACCACGTCCGCTTCATGGATGCCTTCATCGAAGCCCTGGGTCTGGAAGAGGTCG
TCCTGGTCATTCACGACTGGGGCTCCGCTCTGGGTTTCCACTGGGCCAAGCGCAATCCAGAGCGCGTC
AAAGGTATTGCATTTATGGAGTTCATCCGCCCTATCCCGACCTGGGACGAATGGCCAGAATTTGCCCG
CGAGACCTTCCAGGCCTTCCGCACCACCGACGTCGGCCGCAAGCTGATCATCGATCAGAACGTTTTTA
TCGAGGGTACGCTGCCGATGGGTGTCGTCCGCCCGCTGACTGAAGTCGAGATGGACCATTACCGCGAG
CCGTTCCTGAATCCTGTTGACCGCGAGCCACTGTGGCGCTTCCCAAACGAGCTGCCAATCGCCGGTGA
GCCAGCGAACATCGTCGCGCTGGTCGAAGAATACATGGACTGGCTGCACCAGTCCCCTGTCCCGAAGC
TGCTGTTCTGGGGCACCCCAGGCGTTCTGATCCCACCGGCCGAAGCCGCTCGCCTGGCCAAAAGCCTG
CCTAACTGCAAGGCTGTGGACATCGGCCCGGGTCTGAATCTGCTGCAAGAAGACAACCCGGACCTGAT
CGGCAGCGAGATCGCGCGCTGGCTGTCGACGCTCGAGAAGCCAACCGGCGCGCCAGGGTCTGGCGTGA
GCAAGGGCGAGGAGCTGTTCACCGGGGTGGTGCCCATCCTGGTCGAGCTGGACGGCGACGTAAACGGC
CACAAGTTCAGCGTGTCCGGCGAGGGCGAGGGCGATGCCACCTACGGCAAGCTGACCCTGAAGTTCAT
CTGCACCACCGGCAAGCTGCCCGTGCCCTGGCCCACCCTCGTGACCACCCTGACCTACGGCGTGCAGT
GCTTCAGCCGCTACCCCGACCACATGAAGCAGCACGACTTCTTCAAGTCCGCCATGCCCGAAGGCTAC
GTCCAGGAGCGCACCATCTTCTTCAAGGACGACGGCAACTACAAGACCCGCGCCGAGGTGAAGTTCGA
GGGCGACACCCTGGTGAACCGCATCGAGCTGAAGGGCATCGACTTCAAGGAGGACGGCAACATCCTGG
GGCACAAGCTGGAGTACAACTACAACAGCCACAACGTCTATATCATGGCCGACAAGCAGAAGAACGGC
ATCAAGGTGAACTTCAAGATCCGCCACAACATCGAGGACGGCAGCGTGCAGCTCGCCGACCACTACCA
GCAGAACACCCCCATCGGCGACGGCCCCGTGCTGCTGCCCGACAACCACTACCTGAGCACCCAGTCCG
CCCTGAGCAAAGACCCCAACGAGAAGCGCGATCACATGGTCCTGCTGGAGTTCGTGACCGCCGCCGGG
ATCACTCTCGGCATGGACGAGCTGTACAAGCTCGAGGACAAGGACAACACCGTGCCCCTGAAGCTGAT
CGCCCTGCTGGCCAACGGCGAGTTCCACTCTGGCGAGCAGCTGGGAGAGACCCTGGGAATGAGCAGAG
CCGCCATCAACAAGCACATCCAGACACTGAGAGACTGGGGAGTGGACGTGTTCACCGTGCCTGGCAAG
GGCTACAGCCTGCCTGAGCCTATCCAGCTGCTGAACGCCAAGCAGATCCTGGGACAGCTGGATGGCGG

AAGCGTGGCCGTGCTGCCTGTGATCGACTCCACCAATCAGTACCTGCTGGACAGAATCGGAGAGCTGA
AGTCCGGCGACGCCTGCATCGCCGAGTACCAGCAGGCTGGCAGAGGAGGCAGAGGACGGAAGTGGTTC
AGCCCATTCGGAGCCAACCTGTACCTGTCCATGTTCTGGAGACTGGAGCAGGGACCTGCTGCTGCCAT
CGGACTGAGTCTGGTGATCGGAATCGTGATGGCCGAGGTGCTGAGAAAGCTGGGAGCCGACAAGGTGA
GAGTGAAGTGGCCTAATGACCTGTACCTCCAGGACCGCAAGCTGGCTGGCATCCTGGTGGAGCTGACA
GGCAAGACAGGCGATGCCGCTCAGATCGTGATCGGAGCCGGAATCAACATGGCCATGAGAAGAGTGGA
GGAGAGCGTGGTGAACCAGGGCTGGATCACCCTGCAGGAGGCTGGCATCAACCTGGACCGGAACACCC
TGGCCGCCATGCTGATCAGAGAGCTGAGAGCCGCTCTGGAGCTGTTCGAGCAGGAGGGACTGGCTCCT
TACCTGAGCAGATGGGAGAAGCTGGACAACTTCATCAACAGACCTGTGAAGCTGATCATCGGCGACAA
GGAAATCTTCGGCATCTCCAGAGGAATCGACAAGCAGGGAGCTCTGCTGCTGGAGCAGGACGGAATCA
TCAAGCCCTGGATGGGCGGAGAAATCTCCCTGAGAAGCGCAGAGAAGTAA

XLone-puro SNAP-eGFP-BASU

blue = CDS SNAP-eGFP-BASU

gold = CDS Tet-On®3G-P2A-PuroR

ATCACCTCGAGTTTACTCCCTATCAGTGATAGAGAACGTATGAAGAGTTTACTCCCTATCAGTGATAG
AGAACGTATGCAGACTTTACTCCCTATCAGTGATAGAGAACGTATAAGGAGTTTACTCCCTATCAGTG
ATAGAGAACGTATGACCAGTTTACTCCCTATCAGTGATAGAGAACGTATCTACAGTTTACTCCCTATC
AGTGATAGAGAACGTATATCCAGTTTACTCCCTATCAGTGATAGAGAACGTATAAGCTTTGCTTATGT
AAACCAGGGCGCCTATAAAAGAGTGCTGATTTTTTGAGTAAACTTCAATTCCACAACACTTTTGTCTT
ATACCAACTTTCCGTACCACTTCCTACCCTCGTAAAGGTACCGCGGCCGCATGGCCGGCGACAAAGAC
TGCGAAATGAAGCGCACCACCCTGGATAGCCCTCTGGGCAAGCTGGAACTGTCTGGGTGCGAACAGGG
CCTGCACCGTATCATCTTCCTGGGCAAAGGAACATCTGCCGCCGACGCCGTGGAAGTGCCTGCCCCAG
CCGCCGTGCTGGGCGGACCAGAGCCACTGATGCAGGCCACCGCCTGGCTCAACGCCTACTTTCACCAG
CCTGAGGCCATCGAGGAGTTCCCTGTGCCAGCCCTGCACCACCCAGTGTTCCAGCAGGAGAGCTTTAC
CCGCCAGGTGCTGTGGAAACTGCTGAAAGTGGTGAAGTTCGGAGAGGTCATCAGCTACAGCCACCTGG
CCGCCCTGGCCGGCAATCCCGCCGCCACCGCCGCCGTGAAAACCGCCCTGAGCGGAAATCCCGTGCCC
ATTCTGATCCCCTGCCACCGGGTGGTGCAGGGCGACCTGGACGTGGGGGGCTACGAGGGCGGGCTCGC
CGTGAAAGAGTGGCTGCTGGCCCACGAGGGCCACAGACTGGGCAAGCCTGGGCTGGGTCCTGCAGGCG
GAGGCGCGCCAGGGTCTGGCGTGAGCAAGGGCGAGGAGCTGTTCACCGGGGTGGTGCCCATCCTGGTC
GAGCTGGACGGCGACGTAAACGGCCACAAGTTCAGCGTGTCCGGCGAGGGCGAGGGCGATGCCACCTA
CGGCAAGCTGACCCTGAAGTTCATCTGCACCACCGGCAAGCTGCCCGTGCCCTGGCCCACCCTCGTGA
CCACCCTGACCTACGGCGTGCAGTGCTTCAGCCGCTACCCCGACCACATGAAGCAGCACGACTTCTTC
AAGTCCGCCATGCCCGAAGGCTACGTCCAGGAGCGCACCATCTTCTTCAAGGACGACGGCAACTACAA
GACCCGCGCCGAGGTGAAGTTCGAGGGCGACACCCTGGTGAACCGCATCGAGCTGAAGGGCATCGACT
TCAAGGAGGACGGCAACATCCTGGGGCACAAGCTGGAGTACAACTACAACAGCCACAACGTCTATATC
ATGGCCGACAAGCAGAAGAACGGCATCAAGGTGAACTTCAAGATCCGCCACAACATCGAGGACGGCAG
CGTGCAGCTCGCCGACCACTACCAGCAGAACACCCCCATCGGCGACGGCCCCGTGCTGCTGCCCGACA
ACCACTACCTGAGCACCCAGTCCGCCCTGAGCAAAGACCCCAACGAGAAGCGCGATCACATGGTCCTG
CTGGAGTTCGTGACCGCCGCCGGGATCACTCTCGGCATGGACGAGCTGTACAAGCCGGTCACCGGCGG
AAAACTTAGCGAGAGCGAGATCCGCTTCGGCCTGAAAACCGAAGTGATGGGCCAGCACCTGATCTACC
ACGACGTGCTGAGCAGCACCCAGAAAACAGCCCACGAGCTGGCCAACAACAATGCCCCTGAGGGAACA
CTGGTGGTGGCCGATAAGCAGACAGCTGGCCGCGGCGGAATGAGCCGCGTGTGGCACAGCCAAGAAGG
CAACGGCGTCTGGATGAGCCTGATCCTGCGCCCTGACATCCCTCTCCAGAAAACCCCTCAGCTGACAC
TGCTGGCTGCTGTGGCTGTGGTGCAGGGAATTGAAGAAGCCGCCGGAATCCAGACCGACATCAAGTGG
CCCAACGACATCCTGATCAACGGCAAGAAAACCGTGGGCATCCTGACCGAGATGCAGGCCGAAGAGGA
CAGAGTCCGCAGCGTGATCATCGGCATCGGGATCAATGTGAACCAGCAGCCTAACGACTTCCCCGACG
AGCTGAAGGATATCGCCACAAGCCTGTCTCAGGCCGCTGGCGAGAAGATTGATCGCGCTGGCGTGATC
CAGCACATCCTGCTGTGCTTCGAGAAGCGGTACCGCGACTACATGACCCACGGCTTCACCCCTATCAA
ACTGCTGTGGGAGAGCTACGCCCTCGGAATCGGCACCAACATGCGCGCCCGCACACTGAACGGCACCT
TCTACGGCAAGGCCCTGGGAATTGATGACGAGGGCGTTCTGCTGCTGGAAACAAACGAGGGCATCAAG
AAAATCTACAGCGCCGACATCAGCCTGCGCTAATTCTAGTAGACCACCTCCCCTGCGAGCTAAGCTGG
ACAGCCAATGACGGGTAAGAGAGTGACATTTTTCACTAACCTAAGACAGGAGGGCCGTCAGAGCTACT
GCCTAATCCAAAGACGGGTAAAAGTGATAAAAATGTATCACTCCAACCTAAGACAGGCGCAGCTTCCG
AGGGATTTGAGATCCAGACATGATAAGATACATTGATGAGTTTGGACAAACCAAAACTAGAATGCAGT

GAAAAAAATGCCTTATTTGTGAAATTTGTGATGCTATTGCCTTATTTGTAACCATTATAAGCTGCAAT
AAACAAGTTTGATATCTATAACAAGAAAATATATATATAATAAGTTATCACGTAAGTAGAACATGAAA
TAACAATATAATTATCGTATGAGTTAAATCTTAAAAGTCACGTAAAAGATAATCATGCGTCATTTTGA
CTCACGCGGTCGTTATAGTTCAAAATCAGTGACACTTACCGCATTGACAAGCACGCCTCACGGGAGCT
CCAAGCGGCGACTGAGATGTCCTAAATGCACAGCGACGGATTCGCGCTATTTAGAAAGAGAGAGCAAT
ATTTCAAGAATGCATGCGTCAATTTTACGCAGACTATCTTTCTAGGGTTAAGAATTCACTGGCCGTCG
TTTTACAACGTCGTGACTGGGAAAACCCTGGCGTTACCCAACTTAATCGCCTTGCAGCACATCCCCCT
TTCGCCAGCTGGCGTAATAGCGAAGAGGCCCGCACCGATCGCCCTTCCCAACAGTTGCGCAGCCTGAA
TGGCGAATGGCGCCTGATGCGGTATTTTCTCCTTACGCATCTGTGCGGTATTTCACACCGCATATGGT
GCACTCTCAGTACAATCTGCTCTGATGCCGCATAGTTAAGCCAGCCCCGACACCCGCCAACACCCGCT
GACGCGCCCTGACGGGCTTGTCTGCTCCCGGCATCCGCTTACAGACAAGCTGTGACCGTCTCCGGGAG
CTGCATGTGTCAGAGGTTTTCACCGTCATCACCGAAACGCGCGAGACGAAAGGGCCTCGTGATACGCC
TATTTTTATAGGTTAATGTCATGATAATAATGGTTTCTTAGACGTCAGGTGGCACTTTTCGGGGAAAT
GTGCGCGGAACCCCTATTTGTTTATTTTTCTAAATACATTCAAATATGTATCCGCTCATGAGACAATA
ACCCTGATAAATGCTTCAATAATATTGAAAAAGGAAGAGTATGAGTATTCAACATTTCCGTGTCGCCC
TTATTCCCTTTTTTGCGGCATTTTGCCTTCCTGTTTTTGCTCACCCAGAAACGCTGGTGAAAGTAAAA
GATGCTGAAGATCAGTTGGGTGCACGAGTGGGTTACATCGAACTGGATCTCAACAGCGGTAAGATCCT
TGAGAGTTTTCGCCCCGAAGAACGTTTTCCAATGATGAGCACTTTTAAAGTTCTGCTATGTGGCGCGG
TATTATCCCGTATTGACGCCGGGCAAGAGCAACTCGGTCGCCGCATACACTATTCTCAGAATGACTTG
GTTGAGTACTCACCAGTCACAGAAAAGCATCTTACGGATGGCATGACAGTAAGAGAATTATGCAGTGC
TGCCATAACCATGAGTGATAACACTGCGGCCAACTTACTTCTGACAACGATCGGAGGACCGAAGGAGC
TAACCGCTTTTTTGCACAACATGGGGGATCATGTAACTCGCCTTGATCGTTGGGAACCGGAGCTGAAT
GAAGCCATACCAAACGACGAGCGTGACACCACGATGCCTGTAGCAATGGCAACAACGTTGCGCAAACT
ATTAACTGGCGAACTACTTACTCTAGCTTCCCGGCAACAATTAATAGACTGGATGGAGGCGGATAAAG
TTGCAGGACCACTTCTGCGCTCGGCCCTTCCGGCTGGCTGGTTTATTGCTGATAAATCTGGAGCCGGT
GAGCGTGGGTCTCGCGGTATCATTGCAGCACTGGGGCCAGATGGTAAGCCCTCCCGTATCGTAGTTAT
CTACACGACGGGGAGTCAGGCAACTATGGATGAACGAAATAGACAGATCGCTGAGATAGGTGCCTCAC
TGATTAAGCATTGGTAACTGTCAGACCAAGTTTACTCATATATACTTTAGATTGATTTAAAACTTCAT
TTTTAATTTAAAAGGATCTAGGTGAAGATCCTTTTTGATAATCTCATGACCAAAATCCCTTAACGTGA
GTTTTCGTTCCACTGAGCGTCAGACCCCGTAGAAAAGATCAAAGGATCTTCTTGAGATCCTTTTTTTC
TGCGCGTAATCTGCTGCTTGCAAACAAAAAAACCACCGCTACCAGCGGTGGTTTGTTTGCCGGATCAA
GAGCTACCAACTCTTTTTCCGAAGGTAACTGGCTTCAGCAGAGCGCAGATACCAAATACTGTTCTTCT
AGTGTAGCCGTAGTTAGGCCACCACTTCAAGAACTCTGTAGCACCGCCTACATACCTCGCTCTGCTAA
TCCTGTTACCAGTGGCTGCTGCCAGTGGCGATAAGTCGTGTCTTACCGGGTTGGACTCAAGACGATAG
TTACCGGATAAGGCGCAGCGGTCGGGCTGAACGGGGGGTTCGTGCACACAGCCCAGCTTGGAGCGAAC
GACCTACACCGAACTGAGATACCTACAGCGTGAGCTATGAGAAAGCGCCACGCTTCCCGAAGGGAGAA
AGGCGGACAGGTATCCGGTAAGCGGCAGGGTCGGAACAGGAGAGCGCACGAGGGAGCTTCCAGGGGGA
AACGCCTGGTATCTTTATAGTCCTGTCGGGTTTCGCCACCTCTGACTTGAGCGTCGATTTTTGTGATG
CTCGTCAGGGGGGCGGAGCCTATGGAAAAACGCCAGCAACGCGGCCTTTTTACGGTTCCTGGCCTTTT
GCTGGCCTTTTGCTCACATGTTCTTTCCTGCGTTATCCCCTGATTCTGTGGATAACCGTATTACCGCC
TTTGAGTGAGCTGATACCGCTCGCCGCAGCCGAACGACCGAGCGCAGCGAGTCAGTGAGCGAGGAAGC
GGAAGAGCGCCCAATACGCAAACCGCCTCTCCCCGCGCGTTGGCCGATTCATTAATGCAGCTGGCACG
ACAGGTTTCCCGACTGGAAAGCGGGCAGTGAGCGCAACGCAATTAATGTGAGTTAGCTCACTCATTAG
GCACCCCAGGCTTTACACTTTATGCTTCCGGCTCGTATGTTGTGTGGAATTGTGAGCGGATAACAATT
TCACACAGGAAACAGCTATGACCATGATTACGCCAAGGTCGACTTAACCCTAGAAAGATAATCATATT
GTGACGTACGTTAAAGATAATCATGCGTAAAATTGACGCATGTGTTTTATCGGTCTGTATATCGAGGT
TTATTTATTAATTTGAATAGATATTAAGTTTTATTATATTTACACTTACATACTAATAATAAATTCAA
CAAACAATTTATTTATGTTTATTTATTTATTAAAAAAAAACAAAAACTCAAAATTTCTTCTATAAAGT
AACAAAACTTTTAGCAGTGAAAAAAATGCTTTATTTGTGAAATTTGTGATGCTATTGCTTTATTTGTA
ACCATTATAAGCTGCAATAAACAAGTTAACAACAACAATTGCATTCATTTTATGTTTCAGGTTCAGGG
GGAGGTGTGGGAGGTTTTTTAAAGCAAGTAAAACCTCTACAAATGTGGTATGGCTGATTATGATCCTC
TGGAGATCCTAGGCTAGGCACCGGGCTTGCGGGTCATGCACCAGGTGCGCGGTCCTTCGGGCACCTCG
ACGTCGGCGGTGACGGTGAAGCCGAGCCGCTCGTAGAAGGGGAGGTTGCGGGGCGCGGAGGTCTCCAG
GAAGGCGGGCACCCCGGCGCGCTCGGCCGCCTCCACTCCGGGGAGCACGACGGCGCTGCCCAGACCCT
TGCCCTGGTGGTCGGGCGAGACGCCGACGGTGGCCAGGAACCACGCGGGCTCCTTGGGCCGGTGCGGC
GCCAGGAGGCCTTCCATCTGTTGCTGCGCGGCCAGCCGGGAACCGCTCAACTCGGCCATGCGCGGGCC

GATCTCGGCGAACACCGCCCCCGCTTCGACGCTCTCCGGCGTGGTCCAGACCGCCACCGCGGCGCCGT
CGTCCGCGACCCACACCTTGCCGATGTCGAGCCCGACGCGCGTGAGGAAGAGTTCTTGCAGCTCGGTG
ACCCGCTCGATGTGGCGGTCCGGATCGACGGTGTGGCGCGTGGCGGGGTAGTCGGCGAACGCGGCGGC
GAGGGTGCGTACGGCCCTGGGGACGTCGTCGCGGGTGGCGAGGCGCACCGTGGGCTTGTACTCGGTCA
TGGGGCCGGGGTTCTCCTCCACGTCGCCGGCCTGCTTCAGCAGGCTGAAGTTGGTGGCGCCGCTGCCC
CCGGGGAGCATGTCAAGGTCAAAATCGTCAAGAGCGTCAGCAGGCAGCATATCAAGGTCAAAGTCGTC
AAGGGCATCGGCTGGGAGCATGTCTAAGTCAAAATCGTCAAGGGCGTCGGTCGGCCCGCCGCTTTCGC
ACTTTAGCTGTTTCTCCAGGCCACATATGATTAGTTCCAGGCCGAAAAGGAAGGCAGGTTCGGCTCCC
TGCCGGTCGAACAGCTCAATTGCTTGTTTCAGAAGTGGGGGCATAGAATCGGTGGTAGGTGTCTCTCT
TTCCTCTTTTGCTACTTGATGCTCCTGTTCCTCCAATACGCAGCCCAGTGTAAAGTGGCCCACGGCGG
ACAGAGCGTACAGTGCGTTCTCCAGGGAGAAGCCTTGCTGACACAGGAACGCGAGCTGATTTTCCAGG
GTTTCGTACTGTTTCTCTGTTGGGCGGGTGCCGAGATGCACTTTAGCCCCGTCGCGATGTGAGAGGAG
AGCACAGCGGTATGACTTGGCGTTGTTCCGCAGAAAGTCTTGCCATGACTCGCCTTCCAGGGGGCAGG
AGTGGGTATGATGCCTGTCCAGCATCTCGATTGGCAGGGCATCGAGCAGGGCCCGCTTGTTCTTCACG
TGCCAGTACAGGGTAGGCTGCTCAACTCCCAGCTTTTGAGCGAGTTTCCTTGTCGTCAGGCCTTCGAT
ACCGACTCCATTGAGTAATTCCAGAGCAGAGTTTATGACTTTGCTCTTGTCCAGTCTAGACATCTTAT
CGTCATCGTCTTTGTAATCCATGGTGGCGGATCCCGCGTCACGACACCTGTGTTCTGGCGGCAAACCC
GTTGCGAAAAAGAACGTTCACGGCGACTACTGCACTTATATACGGTTCTCCCCCACCCTCGGGAAAAA
GGCGGAGCCAGTACACGACATCACTTTCCCAGTTTACCCCGCGCCACCTTCTCTAGGCACCGGTTCAA
TTGCCGACCCCTCCCCCCAACTTCTCGGGGACTGTGGGCGATGTGCGCTCTGCCCACTGACGGGCACC
GGAGCCACTCGAGTGGAATT

XLone-puro NLS-SNAP-eGFP-BASU

blue = CDS NLS-SNAP-eGFP-BASU

gold = CDS Tet-On®3G-P2A-PuroR

ATCACCTCGAGTTTACTCCCTATCAGTGATAGAGAACGTATGAAGAGTTTACTCCCTATCAGTGATAG
AGAACGTATGCAGACTTTACTCCCTATCAGTGATAGAGAACGTATAAGGAGTTTACTCCCTATCAGTG
ATAGAGAACGTATGACCAGTTTACTCCCTATCAGTGATAGAGAACGTATCTACAGTTTACTCCCTATC
AGTGATAGAGAACGTATATCCAGTTTACTCCCTATCAGTGATAGAGAACGTATAAGCTTTGCTTATGT
AAACCAGGGCGCCTATAAAAGAGTGCTGATTTTTTGAGTAAACTTCAATTCCACAACACTTTTGTCTT
ATACCAACTTTCCGTACCACTTCCTACCCTCGTAAAGGTACCGCGGCCGCCCAAAAAAGAAAAGAAAA
GTTGCTCCAGGCTCAGGTGACAAAGACTGCGAAATGAAGCGCACCACCCTGGATAGCCCTCTGGGCAA
GCTGGAACTGTCTGGGTGCGAACAGGGCCTGCACCGTATCATCTTCCTGGGCAAAGGAACATCTGCCG
CCGACGCCGTGGAAGTGCCTGCCCCAGCCGCCGTGCTGGGCGGACCAGAGCCACTGATGCAGGCCACC
GCCTGGCTCAACGCCTACTTTCACCAGCCTGAGGCCATCGAGGAGTTCCCTGTGCCAGCCCTGCACCA
CCCAGTGTTCCAGCAGGAGAGCTTTACCCGCCAGGTGCTGTGGAAACTGCTGAAAGTGGTGAAGTTCG
GAGAGGTCATCAGCTACAGCCACCTGGCCGCCCTGGCCGGCAATCCCGCCGCCACCGCCGCCGTGAAA
ACCGCCCTGAGCGGAAATCCCGTGCCCATTCTGATCCCCTGCCACCGGGTGGTGCAGGGCGACCTGGA
CGTGGGGGGCTACGAGGGCGGGCTCGCCGTGAAAGAGTGGCTGCTGGCCCACGAGGGCCACAGACTGG
GCAAGCCTGGGCTGGGTCCTGCAGGCGGAGGCGCGCCAGGGTCTGGCGTGAGCAAGGGCGAGGAGCTG
TTCACCGGGGTGGTGCCCATCCTGGTCGAGCTGGACGGCGACGTAAACGGCCACAAGTTCAGCGTGTC
CGGCGAGGGCGAGGGCGATGCCACCTACGGCAAGCTGACCCTGAAGTTCATCTGCACCACCGGCAAGC
TGCCCGTGCCCTGGCCCACCCTCGTGACCACCCTGACCTACGGCGTGCAGTGCTTCAGCCGCTACCCC
GACCACATGAAGCAGCACGACTTCTTCAAGTCCGCCATGCCCGAAGGCTACGTCCAGGAGCGCACCAT
CTTCTTCAAGGACGACGGCAACTACAAGACCCGCGCCGAGGTGAAGTTCGAGGGCGACACCCTGGTGA
ACCGCATCGAGCTGAAGGGCATCGACTTCAAGGAGGACGGCAACATCCTGGGGCACAAGCTGGAGTAC
AACTACAACAGCCACAACGTCTATATCATGGCCGACAAGCAGAAGAACGGCATCAAGGTGAACTTCAA
GATCCGCCACAACATCGAGGACGGCAGCGTGCAGCTCGCCGACCACTACCAGCAGAACACCCCCATCG
GCGACGGCCCCGTGCTGCTGCCCGACAACCACTACCTGAGCACCCAGTCCGCCCTGAGCAAAGACCCC
AACGAGAAGCGCGATCACATGGTCCTGCTGGAGTTCGTGACCGCCGCCGGGATCACTCTCGGCATGGA
CGAGCTGTACAAGCCGGTCACCGGCGGAAAACTTAGCGAGAGCGAGATCCGCTTCGGCCTGAAAACCG
AAGTGATGGGCCAGCACCTGATCTACCACGACGTGCTGAGCAGCACCCAGAAAACAGCCCACGAGCTG
GCCAACAACAATGCCCCTGAGGGAACACTGGTGGTGGCCGATAAGCAGACAGCTGGCCGCGGCGGAAT
GAGCCGCGTGTGGCACAGCCAAGAAGGCAACGGCGTCTGGATGAGCCTGATCCTGCGCCCTGACATCC

CTCTCCAGAAAACCCCTCAGCTGACACTGCTGGCTGCTGTGGCTGTGGTGCAGGGAATTGAAGAAGCC
GCCGGAATCCAGACCGACATCAAGTGGCCCAACGACATCCTGATCAACGGCAAGAAAACCGTGGGCAT
CCTGACCGAGATGCAGGCCGAAGAGGACAGAGTCCGCAGCGTGATCATCGGCATCGGGATCAATGTGA
ACCAGCAGCCTAACGACTTCCCCGACGAGCTGAAGGATATCGCCACAAGCCTGTCTCAGGCCGCTGGC
GAGAAGATTGATCGCGCTGGCGTGATCCAGCACATCCTGCTGTGCTTCGAGAAGCGGTACCGCGACTA
CATGACCCACGGCTTCACCCCTATCAAACTGCTGTGGGAGAGCTACGCCCTCGGAATCGGCACCAACA
TGCGCGCCCGCACACTGAACGGCACCTTCTACGGCAAGGCCCTGGGAATTGATGACGAGGGCGTTCTG
CTGCTGGAAACAAACGAGGGCATCAAGAAAATCTACAGCGCCGACATCAGCCTGCGCTAATTCTAGTA
GACCACCTCCCCTGCGAGCTAAGCTGGACAGCCAATGACGGGTAAGAGAGTGACATTTTTCACTAACC
TAAGACAGGAGGGCCGTCAGAGCTACTGCCTAATCCAAAGACGGGTAAAAGTGATAAAAATGTATCAC
TCCAACCTAAGACAGGCGCAGCTTCCGAGGGATTTGAGATCCAGACATGATAAGATACATTGATGAGT
TTGGACAAACCAAAACTAGAATGCAGTGAAAAAAATGCCTTATTTGTGAAATTTGTGATGCTATTGCC
TTATTTGTAACCATTATAAGCTGCAATAAACAAGTTTGATATCTATAACAAGAAAATATATATATAAT
AAGTTATCACGTAAGTAGAACATGAAATAACAATATAATTATCGTATGAGTTAAATCTTAAAAGTCAC
GTAAAAGATAATCATGCGTCATTTTGACTCACGCGGTCGTTATAGTTCAAAATCAGTGACACTTACCG
CATTGACAAGCACGCCTCACGGGAGCTCCAAGCGGCGACTGAGATGTCCTAAATGCACAGCGACGGAT
TCGCGCTATTTAGAAAGAGAGAGCAATATTTCAAGAATGCATGCGTCAATTTTACGCAGACTATCTTT
CTAGGGTTAAGAATTCACTGGCCGTCGTTTTACAACGTCGTGACTGGGAAAACCCTGGCGTTACCCAA
CTTAATCGCCTTGCAGCACATCCCCCTTTCGCCAGCTGGCGTAATAGCGAAGAGGCCCGCACCGATCG
CCCTTCCCAACAGTTGCGCAGCCTGAATGGCGAATGGCGCCTGATGCGGTATTTTCTCCTTACGCATC
TGTGCGGTATTTCACACCGCATATGGTGCACTCTCAGTACAATCTGCTCTGATGCCGCATAGTTAAGC
CAGCCCCGACACCCGCCAACACCCGCTGACGCGCCCTGACGGGCTTGTCTGCTCCCGGCATCCGCTTA
CAGACAAGCTGTGACCGTCTCCGGGAGCTGCATGTGTCAGAGGTTTTCACCGTCATCACCGAAACGCG
CGAGACGAAAGGGCCTCGTGATACGCCTATTTTTATAGGTTAATGTCATGATAATAATGGTTTCTTAG
ACGTCAGGTGGCACTTTTCGGGGAAATGTGCGCGGAACCCCTATTTGTTTATTTTTCTAAATACATTC
AAATATGTATCCGCTCATGAGACAATAACCCTGATAAATGCTTCAATAATATTGAAAAAGGAAGAGTA
TGAGTATTCAACATTTCCGTGTCGCCCTTATTCCCTTTTTTGCGGCATTTTGCCTTCCTGTTTTTGCT
CACCCAGAAACGCTGGTGAAAGTAAAAGATGCTGAAGATCAGTTGGGTGCACGAGTGGGTTACATCGA
ACTGGATCTCAACAGCGGTAAGATCCTTGAGAGTTTTCGCCCCGAAGAACGTTTTCCAATGATGAGCA
CTTTTAAAGTTCTGCTATGTGGCGCGGTATTATCCCGTATTGACGCCGGGCAAGAGCAACTCGGTCGC
CGCATACACTATTCTCAGAATGACTTGGTTGAGTACTCACCAGTCACAGAAAAGCATCTTACGGATGG
CATGACAGTAAGAGAATTATGCAGTGCTGCCATAACCATGAGTGATAACACTGCGGCCAACTTACTTC
TGACAACGATCGGAGGACCGAAGGAGCTAACCGCTTTTTTGCACAACATGGGGGATCATGTAACTCGC
CTTGATCGTTGGGAACCGGAGCTGAATGAAGCCATACCAAACGACGAGCGTGACACCACGATGCCTGT
AGCAATGGCAACAACGTTGCGCAAACTATTAACTGGCGAACTACTTACTCTAGCTTCCCGGCAACAAT
TAATAGACTGGATGGAGGCGGATAAAGTTGCAGGACCACTTCTGCGCTCGGCCCTTCCGGCTGGCTGG
TTTATTGCTGATAAATCTGGAGCCGGTGAGCGTGGGTCTCGCGGTATCATTGCAGCACTGGGGCCAGA
TGGTAAGCCCTCCCGTATCGTAGTTATCTACACGACGGGGAGTCAGGCAACTATGGATGAACGAAATA
GACAGATCGCTGAGATAGGTGCCTCACTGATTAAGCATTGGTAACTGTCAGACCAAGTTTACTCATAT
ATACTTTAGATTGATTTAAAACTTCATTTTTAATTTAAAAGGATCTAGGTGAAGATCCTTTTTGATAA
TCTCATGACCAAAATCCCTTAACGTGAGTTTTCGTTCCACTGAGCGTCAGACCCCGTAGAAAAGATCA
AAGGATCTTCTTGAGATCCTTTTTTTCTGCGCGTAATCTGCTGCTTGCAAACAAAAAAACCACCGCTA
CCAGCGGTGGTTTGTTTGCCGGATCAAGAGCTACCAACTCTTTTTCCGAAGGTAACTGGCTTCAGCAG
AGCGCAGATACCAAATACTGTTCTTCTAGTGTAGCCGTAGTTAGGCCACCACTTCAAGAACTCTGTAG
CACCGCCTACATACCTCGCTCTGCTAATCCTGTTACCAGTGGCTGCTGCCAGTGGCGATAAGTCGTGT
CTTACCGGGTTGGACTCAAGACGATAGTTACCGGATAAGGCGCAGCGGTCGGGCTGAACGGGGGGTTC
GTGCACACAGCCCAGCTTGGAGCGAACGACCTACACCGAACTGAGATACCTACAGCGTGAGCTATGAG
AAAGCGCCACGCTTCCCGAAGGGAGAAAGGCGGACAGGTATCCGGTAAGCGGCAGGGTCGGAACAGGA
GAGCGCACGAGGGAGCTTCCAGGGGGAAACGCCTGGTATCTTTATAGTCCTGTCGGGTTTCGCCACCT
CTGACTTGAGCGTCGATTTTTGTGATGCTCGTCAGGGGGGCGGAGCCTATGGAAAAACGCCAGCAACG
CGGCCTTTTTACGGTTCCTGGCCTTTTGCTGGCCTTTTGCTCACATGTTCTTTCCTGCGTTATCCCCT
GATTCTGTGGATAACCGTATTACCGCCTTTGAGTGAGCTGATACCGCTCGCCGCAGCCGAACGACCGA
GCGCAGCGAGTCAGTGAGCGAGGAAGCGGAAGAGCGCCCAATACGCAAACCGCCTCTCCCCGCGCGTT
GGCCGATTCATTAATGCAGCTGGCACGACAGGTTTCCCGACTGGAAAGCGGGCAGTGAGCGCAACGCA
ATTAATGTGAGTTAGCTCACTCATTAGGCACCCCAGGCTTTACACTTTATGCTTCCGGCTCGTATGTT
GTGTGGAATTGTGAGCGGATAACAATTTCACACAGGAAACAGCTATGACCATGATTACGCCAAGGTCG

ACTTAACCCTAGAAAGATAATCATATTGTGACGTACGTTAAAGATAATCATGCGTAAAATTGACGCAT
GTGTTTTATCGGTCTGTATATCGAGGTTTATTTATTAATTTGAATAGATATTAAGTTTTATTATATTT
ACACTTACATACTAATAATAAATTCAACAAACAATTTATTTATGTTTATTTATTTATTAAAAAAAAAC
AAAAACTCAAAATTTCTTCTATAAAGTAACAAAACTTTTAGCAGTGAAAAAAATGCTTTATTTGTGAA
ATTTGTGATGCTATTGCTTTATTTGTAACCATTATAAGCTGCAATAAACAAGTTAACAACAACAATTG
CATTCATTTTATGTTTCAGGTTCAGGGGGAGGTGTGGGAGGTTTTTTAAAGCAAGTAAAACCTCTACA
AATGTGGTATGGCTGATTATGATCCTCTGGAGATCCTAGGCTAGGCACCGGGCTTGCGGGTCATGCAC
CAGGTGCGCGGTCCTTCGGGCACCTCGACGTCGGCGGTGACGGTGAAGCCGAGCCGCTCGTAGAAGGG
GAGGTTGCGGGGCGCGGAGGTCTCCAGGAAGGCGGGCACCCCGGCGCGCTCGGCCGCCTCCACTCCGG
GGAGCACGACGGCGCTGCCCAGACCCTTGCCCTGGTGGTCGGGCGAGACGCCGACGGTGGCCAGGAAC
CACGCGGGCTCCTTGGGCCGGTGCGGCGCCAGGAGGCCTTCCATCTGTTGCTGCGCGGCCAGCCGGGA
ACCGCTCAACTCGGCCATGCGCGGGCCGATCTCGGCGAACACCGCCCCCGCTTCGACGCTCTCCGGCG
TGGTCCAGACCGCCACCGCGGCGCCGTCGTCCGCGACCCACACCTTGCCGATGTCGAGCCCGACGCGC
GTGAGGAAGAGTTCTTGCAGCTCGGTGACCCGCTCGATGTGGCGGTCCGGATCGACGGTGTGGCGCGT
GGCGGGGTAGTCGGCGAACGCGGCGGCGAGGGTGCGTACGGCCCTGGGGACGTCGTCGCGGGTGGCGA
GGCGCACCGTGGGCTTGTACTCGGTCATGGGGCCGGGGTTCTCCTCCACGTCGCCGGCCTGCTTCAGC
AGGCTGAAGTTGGTGGCGCCGCTGCCCCCGGGGAGCATGTCAAGGTCAAAATCGTCAAGAGCGTCAGC
AGGCAGCATATCAAGGTCAAAGTCGTCAAGGGCATCGGCTGGGAGCATGTCTAAGTCAAAATCGTCAA
GGGCGTCGGTCGGCCCGCCGCTTTCGCACTTTAGCTGTTTCTCCAGGCCACATATGATTAGTTCCAGG
CCGAAAAGGAAGGCAGGTTCGGCTCCCTGCCGGTCGAACAGCTCAATTGCTTGTTTCAGAAGTGGGGG
CATAGAATCGGTGGTAGGTGTCTCTCTTTCCTCTTTTGCTACTTGATGCTCCTGTTCCTCCAATACGC
AGCCCAGTGTAAAGTGGCCCACGGCGGACAGAGCGTACAGTGCGTTCTCCAGGGAGAAGCCTTGCTGA
CACAGGAACGCGAGCTGATTTTCCAGGGTTTCGTACTGTTTCTCTGTTGGGCGGGTGCCGAGATGCAC
TTTAGCCCCGTCGCGATGTGAGAGGAGAGCACAGCGGTATGACTTGGCGTTGTTCCGCAGAAAGTCTT
GCCATGACTCGCCTTCCAGGGGGCAGGAGTGGGTATGATGCCTGTCCAGCATCTCGATTGGCAGGGCA
TCGAGCAGGGCCCGCTTGTTCTTCACGTGCCAGTACAGGGTAGGCTGCTCAACTCCCAGCTTTTGAGC
GAGTTTCCTTGTCGTCAGGCCTTCGATACCGACTCCATTGAGTAATTCCAGAGCAGAGTTTATGACTT
TGCTCTTGTCCAGTCTAGACATCTTATCGTCATCGTCTTTGTAATCCATGGTGGCGGATCCCGCGTCA
CGACACCTGTGTTCTGGCGGCAAACCCGTTGCGAAAAAGAACGTTCACGGCGACTACTGCACTTATAT
ACGGTTCTCCCCCACCCTCGGGAAAAAGGCGGAGCCAGTACACGACATCACTTTCCCAGTTTACCCCG
CGCCACCTTCTCTAGGCACCGGTTCAATTGCCGACCCCTCCCCCCAACTTCTCGGGGACTGTGGGCGA
TGTGCGCTCTGCCCACTGACGGGCACCGGAGCCACTCGAGTGGAATT

pcDNA3.1_mPB

GACGGATCGGGAGATCTCCCGATCCCCTATGGTGCACTCTCAGTACAATCTGCTCTGATGCCGCATAG
TTAAGCCAGTATCTGCTCCCTGCTTGTGTGTTGGAGGTCGCTGAGTAGTGCGCGAGCAAAATTTAAGC
TACAACAAGGCAAGGCTTGACCGACAATTGCATGAAGAATCTGCTTAGGGTTAGGCGTTTTGCGCTGC
TTCGCGATGTACGGGCCAGATATACGCGTTGACATTGATTATTGACTAGTGATTATTGAGCCATAGAA
TTCGAGCTTGCATGCCTGCAGGTCGTTACATAACTTACGGTAAATGGCCCGCCTGGCTGACCGCCCAA
CGACCCCCGCCCATTGACGTCAATAATGACGTATGTTCCCATAGTAACGCCAATAGGGACTTTCCATT
GACGTCAATGGGTGGAGTATTTACGGTAAACTGCCCACTTGGCAGTACATCAAGTGTATCATATGCCA
AGTACGCCCCCTATTGACGTCAATGACGGTAAATGGCCCGCCTGGCATTATGCCCAGTACATGACCTT
ATGGGACTTTCCTACTTGGCAGTACATCTACGTATTAGTCATCGCTATTACCATGGTGATGCGGTTTT
GGCAGTACATCAATGGGCGTGGATAGCGGTTTGACTCACGGGGATTTCCAAGTCTCCACCCCATTGAC
GTCAATGGGAGTTTGTTTTGGCACCAAAATCAACGGGACTTTCCAAAATGTCGTAACAACTCCGCCCC
ATTGACGCAAATGGGCGGTAGGCGTGTACGGTGGGAGGTCTATATAAGCAGAGCTCGTTTAGTGAACC
GTCAGATCGCCTGGAGACGCCATCCACGCTGTTTTGACCTCCATAGAAGACACCGGGACCGATCCAGC
CTCCGGACTCTAGAGGATCCGGTACTCGAGGAACTGAAAAACCAGAAAGTTAACTGGTAAGTTTAGTC
TTTTTGTCTTTTATTTCAGGTCCCGGATCCGGTGGTGGTGCAAATCAAAGAACTGCTCCTCAGTGGAT
GTTGCCTTTACTTCTAGGCCTGTACGGAAGTGTTACTTCTGCTCTAAAAGCTGCGGAATTGTACCCAA
TTCGTTAAGGCCAAATTGGCCACCATGGGCTCTAGCCTGGACGACGAGCACATCCTGAGCGCCCTGCT
GCAGAGCGACGACGAACTGGTGGGCGAGGACAGCGACAGCGAGGTCAGCGACCACGTGTCCGAGGACG
ACGTGCAGTCCGACACCGAGGAAGCCTTCATCGACGAGGTGCACGAAGTGCAGCCTACCAGCAGCGGC
TCCGAGATCCTGGACGAGCAGAACGTGATCGAGCAGCCTGGCAGCTCCCTGGCCAGCAACAGAATCCT
GACCCTGCCCCAGAGAACCATCAGAGGCAAGAACAAGCACTGCTGGTCCACCTCCAAGAGCACCAGGC

GGAGCAGAGTGTCCGCCCTGAACATCGTGCGGAGCCAGAGGGGCCCCACCAGAATGTGCAGAAACATC
TACGACCCCCTGCTGTGCTTCAAGCTGTTCTTCACCGACGAGATCATCAGCGAGATCGTGAAGTGGAC
CAACGCCGAGATCAGCCTGAAGAGGCGGGAGAGCATGACCAGCGCCACCTTCAGAGACACCAACGAGG
ACGAGATCTACGCCTTCTTCGGCATCCTGGTGATGACCGCCGTGAGAAAGGACAACCACATGAGCACC
GACGACCTGTTCGACAGATCCCTGAGCATGGTGTACGTGTCCGTGATGAGCAGAGACAGATTCGACTT
CCTGATCAGATGCCTGAGAATGGACGACAAGAGCATCAGACCCACCCTGCGGGAGAACGACGTGTTCA
CCCCCGTGCGGAAGATCTGGGACCTGTTCATCCACCAGTGCATCCAGAACTACACCCCTGGCGCCCAC
CTGACCATCGATGAGCAGCTGCTGGGCTTCAGAGGCAGATGCCCCTTCAGAGTGTACATCCCCAACAA
GCCCAGCAAGTACGGCATCAAGATCCTGATGATGTGCGACAGCGGCACCAAGTACATGATCAACGGCA
TGCCCTACCTGGGCAGAGACACCCAGACAAACGGCGTGCCCCTGGGCGAGTACTACGTGAAAGAACTG
AGCAAGCCTGTGCATGGCAGCTGCAGGAACATCACCTGCGACAACTGGTTCACCAGCATCCCCCTGGC
CAAGAACCTGCTGCAGGAACCCTACAAGCTGACCATCGTGGGCACCGTGCGGAGCAACAAGCGGGAGA
TCCCAGAGGTGCTGAAGAACAGCAGATCCAGACCTGTGGGAACAAGCATGTTCTGCTTCGACGGCCCC
CTGACCCTGGTGTCCTACAAGCCCAAGCCCGCCAAGATGGTGTACCTGCTGTCCAGCTGCGACGAGGA
CGCCAGCATCAACGAGAGCACCGGCAAGCCCCAGATGGTGATGTACTACAACCAGACCAAGGGCGGCG
TGGACACCCTGGACCAGATGTGCAGCGTGATGACCTGCAGCAGAAAGACCAACAGATGGCCCATGGCC
CTGCTGTACGGCATGATCAATATCGCCTGCATCAACAGCTTCATCATCTACAGCCACAACGTGTCCAG
CAAGGGCGAGAAGGTGCAGAGCCGGAAGAAATTCATGCGGAACCTGTACATGAGCCTGACCTCCAGCT
TCATGAGAAAGAGACTGGAAGCCCCCACCCTGAAGAGATACCTGCGGGACAACATCAGCAACATCCTG
CCCAAGGAAGTGCCAGGAACAAGCGACGACAGCACCGAGGAACCCGTGATGAAGAAGAGGACCTACTG
CACCTACTGTCCCAGCAAGATCAGAAGAAAGGCCAACGCCAGCTGCAAGAAATGCAAAAAAGTGATCT
GCCGGGAGCACAACATCGACATGTGCCAGAGCTGTTTCTGAGGCCGTAACGGCCGCCAGAATTGGGGA
TCCAGACATGATAAGATACATTGATGAGTTTGGACAAACCACAACTAGAATGCAGTGAAAAAAATGCT
TTATTTGTGAAATTTGTGATGCTATTGCTTTATTTGTAACCATTATAAGCTGCAATAAACAAGTTAAC
GCGGAAGCTTGGTACCGAGCTCGGATCCACTAGCGGCCGCTCGAGTCTAGAGGGCCCTTCGAACAAAA
ACTCATCTCAGAAGAGGATCTGAATATGCATACCGGTCATCATCACCATCACCATTGAGTTTAAACCC
GCTGATCAGCCTCGACTGTGCCTTCTAGTTGCCAGCCATCTGTTGTTTGCCCCTCCCCCGTGCCTTCC
TTGACCCTGGAAGGTGCCACTCCCACTGTCCTTTCCTAATAAAATGAGGAAATTGCATCGCATTGTCT
GAGTAGGTGTCATTCTATTCTGGGGGGTGGGGTGGGGCAGGACAGCAAGGGGGAGGATTGGGAAGACA
ATAGCAGGCATGCTGGGGATGCGGTGGGCTCTATGGCTTCTGAGGCGGAAAGAACCAGCTGGGGCTCT
AGGGGGTATCCCCACGCGCCCTGTAGCGGCGCATTAAGCGCGGCGGGTGTGGTGGTTACGCGCAGCGT
GACCGCTACACTTGCCAGCGCCCTAGCGCCCGCTCCTTTCGCTTTCTTCCCTTCCTTTCTCGCCACGT
TCGCCGGCTTTCCCCGTCAAGCTCTAAATCGGGGGCTCCCTTTAGGGTTCCGATTTAGTGCTTTACGG
CACCTCGACCCCAAAAAACTTGATTAGGGTGATGGTTCACGTAGTGGGCCATCGCCCTGATAGACGGT
TTTTCGCCCTTTGACGTTGGAGTCCACGTTCTTTAATAGTGGACTCTTGTTCCAAACTGGAACAACAC
TCAACCCTATCTCGGTCTATTCTTTTGATTTATAAGGGATTTTGCCGATTTCGGCCTATTGGTTAAAA
AATGAGCTGATTTAACAAAAATTTAACGCGAATTAATTCTGTGGAATGTGTGTCAGTTAGGGTGTGGA
AAGTCCCCAGGCTCCCCAGCAGGCAGAAGTATGCAAAGCATGCATCTCAATTAGTCAGCAACCAGGTG
TGGAAAGTCCCCAGGCTCCCCAGCAGGCAGAAGTATGCAAAGCATGCATCTCAATTAGTCAGCAACCA
TAGTCCCGCCCCTAACTCCGCCCATCCCGCCCCTAACTCCGCCCAGTTCCGCCCATTCTCCGCCCCAT
GGCTGACTAATTTTTTTTATTTATGCAGAGGCCGAGGCCGCCTCTGCCTCTGAGCTATTCCAGAAGTA
GTGAGGAGGCTTTTTTGGAGGCCTAGGCTTTTGCAAAAAGCTCCCGGGAGCTTGTATATCCATTTTCG
GATCTGATCAAGAGACAGGATGAGGATCGTTTCGCATGATTGAACAAGATGGATTGCACGCAGGTTCT
CCGGCCGCTTGGGTGGAGAGGCTATTCGGCTATGACTGGGCACAACAGACAATCGGCTGCTCTGATGC
CGCCGTGTTCCGGCTGTCAGCGCAGGGGCGCCCGGTTCTTTTTGTCAAGACCGACCTGTCCGGTGCCC
TGAATGAACTGCAGGACGAGGCAGCGCGGCTATCGTGGCTGGCCACGACGGGCGTTCCTTGCGCAGCT
GTGCTCGACGTTGTCACTGAAGCGGGAAGGGACTGGCTGCTATTGGGCGAAGTGCCGGGGCAGGATCT
CCTGTCATCTCACCTTGCTCCTGCCGAGAAAGTATCCATCATGGCTGATGCAATGCGGCGGCTGCATA
CGCTTGATCCGGCTACCTGCCCATTCGACCACCAAGCGAAACATCGCATCGAGCGAGCACGTACTCGG
ATGGAAGCCGGTCTTGTCGATCAGGATGATCTGGACGAAGAGCATCAGGGGCTCGCGCCAGCCGAACT
GTTCGCCAGGCTCAAGGCGCGCATGCCCGACGGCGAGGATCTCGTCGTGACCCATGGCGATGCCTGCT
TGCCGAATATCATGGTGGAAAATGGCCGCTTTTCTGGATTCATCGACTGTGGCCGGCTGGGTGTGGCG
GACCGCTATCAGGACATAGCGTTGGCTACCCGTGATATTGCTGAAGAGCTTGGCGGCGAATGGGCTGA
CCGCTTCCTCGTGCTTTACGGTATCGCCGCTCCCGATTCGCAGCGCATCGCCTTCTATCGCCTTCTTG
ACGAGTTCTTCTGAGCGGGACTCTGGGGTTCGAAATGACCGACCAAGCGACGCCCAACCTGCCATCAC
GAGATTTCGATTCCACCGCCGCCTTCTATGAAAGGTTGGGCTTCGGAATCGTTTTCCGGGACGCCGGC

TGGATGATCCTCCAGCGCGGGGATCTCATGCTGGAGTTCTTCGCCCACCCCAACTTGTTTATTGCAGC
TTATAATGGTTACAAATAAAGCAATAGCATCACAAATTTCACAAATAAAGCATTTTTTTCACTGCATT
CTAGTTGTGGTTTGTCCAAACTCATCAATGTATCTTATCATGTCTGTATACCGTCGACCTCTAGCTAG
AGCTTGGCGTAATCATGGTCATAGCTGTTTCCTGTGTGAAATTGTTATCCGCTCACAATTCCACACAA
CATACGAGCCGGAAGCATAAAGTGTAAAGCCTGGGGTGCCTAATGAGTGAGCTAACTCACATTAATTG
CGTTGCGCTCACTGCCCGCTTTCCAGTCGGGAAACCTGTCGTGCCAGCTGCATTAATGAATCGGCCAA
CGCGCGGGGAGAGGCGGTTTGCGTATTGGGCGCTCTTCCGCTTCCTCGCTCACTGACTCGCTGCGCTC
GGTCGTTCGGCTGCGGCGAGCGGTATCAGCTCACTCAAAGGCGGTAATACGGTTATCCACAGAATCAG
GGGATAACGCAGGAAAGAACATGTGAGCAAAAGGCCAGCAAAAGGCCAGGAACCGTAAAAAGGCCGCG
TTGCTGGCGTTTTTCCATAGGCTCCGCCCCCCTGACGAGCATCACAAAAATCGACGCTCAAGTCAGAG
GTGGCGAAACCCGACAGGACTATAAAGATACCAGGCGTTTCCCCCTGGAAGCTCCCTCGTGCGCTCTC
CTGTTCCGACCCTGCCGCTTACCGGATACCTGTCCGCCTTTCTCCCTTCGGGAAGCGTGGCGCTTTCT
CATAGCTCACGCTGTAGGTATCTCAGTTCGGTGTAGGTCGTTCGCTCCAAGCTGGGCTGTGTGCACGA
ACCCCCCGTTCAGCCCGACCGCTGCGCCTTATCCGGTAACTATCGTCTTGAGTCCAACCCGGTAAGAC
ACGACTTATCGCCACTGGCAGCAGCCACTGGTAACAGGATTAGCAGAGCGAGGTATGTAGGCGGTGCT
ACAGAGTTCTTGAAGTGGTGGCCTAACTACGGCTACACTAGAAGAACAGTATTTGGTATCTGCGCTCT
GCTGAAGCCAGTTACCTTCGGAAAAAGAGTTGGTAGCTCTTGATCCGGCAAACAAACCACCGCTGGTA
GCGGTTTTTTTGTTTGCAAGCAGCAGATTACGCGCAGAAAAAAAGGATCTCAAGAAGATCCTTTGATC
TTTTCTACGGGGTCTGACGCTCAGTGGAACGAAAACTCACGTTAAGGGATTTTGGTCATGAGATTATC
AAAAAGGATCTTCACCTAGATCCTTTTAAATTAAAAATGAAGTTTTAAATCAATCTAAAGTATATATG
AGTAAACTTGGTCTGACAGTTACCAATGCTTAATCAGTGAGGCACCTATCTCAGCGATCTGTCTATTT
CGTTCATCCATAGTTGCCTGACTCCCCGTCGTGTAGATAACTACGATACGGGAGGGCTTACCATCTGG
CCCCAGTGCTGCAATGATACCGCGAGACCCACGCTCACCGGCTCCAGATTTATCAGCAATAAACCAGC
CAGCCGGAAGGGCCGAGCGCAGAAGTGGTCCTGCAACTTTATCCGCCTCCATCCAGTCTATTAATTGT
TGCCGGGAAGCTAGAGTAAGTAGTTCGCCAGTTAATAGTTTGCGCAACGTTGTTGCCATTGCTACAGG
CATCGTGGTGTCACGCTCGTCGTTTGGTATGGCTTCATTCAGCTCCGGTTCCCAACGATCAAGGCGAG
TTACATGATCCCCCATGTTGTGCAAAAAAGCGGTTAGCTCCTTCGGTCCTCCGATCGTTGTCAGAAGT
AAGTTGGCCGCAGTGTTATCACTCATGGTTATGGCAGCACTGCATAATTCTCTTACTGTCATGCCATC
CGTAAGATGCTTTTCTGTGACTGGTGAGTACTCAACCAAGTCATTCTGAGAATAGTGTATGCGGCGAC
CGAGTTGCTCTTGCCCGGCGTCAATACGGGATAATACCGCGCCACATAGCAGAACTTTAAAAGTGCTC
ATCATTGGAAAACGTTCTTCGGGGCGAAAACTCTCAAGGATCTTACCGCTGTTGAGATCCAGTTCGAT
GTAACCCACTCGTGCACCCAACTGATCTTCAGCATCTTTTACTTTCACCAGCGTTTCTGGGTGAGCAA
AAACAGGAAGGCAAAATGCCGCAAAAAAGGGAATAAGGGCGACACGGAAATGTTGAATACTCATACTC
TTCCTTTTTCAATATTATTGAAGCATTTATCAGGGTTATTGTCTCATGAGCGGATACATATTTGAATG
TATTTAGAAAAATAAACAAATAGGGGTTCCGCGCACATTTCCCCGAAAAGTGCCACCTGACGTC

Halo-BASU-His

GCAGAAATCGGTACTGGCTTTCCATTCGACCCCCATTATGTGGAAGTCCTGGGCGAGCGCATGCACTA
CGTCGATGTTGGTCCGCGCGATGGCACCCCTGTGCTGTTCCTGCACGGTAACCCGACCTCCTCCTACG
TGTGGCGCAACATCATCCCGCATGTTGCACCGACCCATCGCTGCATTGCTCCAGACCTGATCGGTATG
GGCAAATCCGACAAACCAGACCTGGGTTATTTCTTCGACGACCACGTCCGCTTCATGGATGCCTTCAT
CGAAGCCCTGGGTCTGGAAGAGGTCGTCCTGGTCATTCACGACTGGGGCTCCGCTCTGGGTTTCCACT
GGGCCAAGCGCAATCCAGAGCGCGTCAAAGGTATTGCATTTATGGAGTTCATCCGCCCTATCCCGACC
TGGGACGAATGGCCAGAATTTGCCCGCGAGACCTTCCAGGCCTTCCGCACCACCGACGTCGGCCGCAA
GCTGATCATCGATCAGAACGTTTTTATCGAGGGTACGCTGCCGATGGGTGTCGTCCGCCCGCTGACTG
AAGTCGAGATGGACCATTACCGCGAGCCGTTCCTGAATCCTGTTGACCGCGAGCCACTGTGGCGCTTC
CCAAACGAGCTGCCAATCGCCGGTGAGCCAGCGAACATCGTCGCGCTGGTCGAAGAATACATGGACTG
GCTGCACCAGTCCCCTGTCCCGAAGCTGCTGTTCTGGGGCACCCCAGGCGTTCTGATCCCACCGGCCG
AAGCCGCTCGCCTGGCCAAAAGCCTGCCTAACTGCAAGGCTGTGGACATCGGCCCGGGTCTGAATCTG
CTGCAAGAAGACAACCCGGACCTGATCGGCAGCGAGATCGCGCGCTGGCTGTCGACGCTCGAGATTTC
CGGCCCTGCAGGCGGAGGCGCGCCAGGGTCTGGCGGCGGAAAACTTAGCGAGAGCGAGATCCGCTTCG
GCCTGAAAACCGAAGTGATGGGCCAGCACCTGATCTACCACGACGTGCTGAGCAGCACCCAGAAAACA
GCCCACGAGCTGGCCAACAACAATGCCCCTGAGGGAACACTGGTGGTGGCCGATAAGCAGACAGCTGG

CCGCGGCGGAATGAGCCGCGTGTGGCACAGCCAAGAAGGCAACGGCGTCTGGATGAGCCTGATCCTGC
GCCCTGACATCCCTCTCCAGAAAACCCCTCAGCTGACACTGCTGGCTGCTGTGGCTGTGGTGCAGGGA
ATTGAAGAAGCCGCCGGAATCCAGACCGACATCAAGTGGCCCAACGACATCCTGATCAACGGCAAGAA
AACCGTGGGCATCCTGACCGAGATGCAGGCCGAAGAGGACAGAGTCCGCAGCGTGATCATCGGCATCG
GGATCAATGTGAACCAGCAGCCTAACGACTTCCCCGACGAGCTGAAGGATATCGCCACAAGCCTGTCT
CAGGCCGCTGGCGAGAAGATTGATCGCGCTGGCGTGATCCAGCACATCCTGCTGTGCTTCGAGAAGCG
GTACCGCGACTACATGACCCACGGCTTCACCCCTATCAAACTGCTGTGGGAGAGCTACGCCCTCGGAA
TCGGCACCAACATGCGCGCCCGCACACTGAACGGCACCTTCTACGGCAAGGCCCTGGGAATTGATGAC
GAGGGCGTTCTGCTGCTGGAAACAAACGAGGGCATCAAGAAAATCTACAGCGCCGACATCAGCCTGCG
CCTCGAGCACCACCACCACCACCAC

SNAP-BASU-His

GACAAAGACTGCGAAATGAAGCGCACCACCCTGGATAGCCCTCTGGGCAAGCTGGAACTGTCTGGGTG
CGAACAGGGCCTGCACCGTATCATCTTCCTGGGCAAAGGAACATCTGCCGCCGACGCCGTGGAAGTGC
CTGCCCCAGCCGCCGTGCTGGGCGGACCAGAGCCACTGATGCAGGCCACCGCCTGGCTCAACGCCTAC
TTTCACCAGCCTGAGGCCATCGAGGAGTTCCCTGTGCCAGCCCTGCACCACCCAGTGTTCCAGCAGGA
GAGCTTTACCCGCCAGGTGCTGTGGAAACTGCTGAAAGTGGTGAAGTTCGGAGAGGTCATCAGCTACA
GCCACCTGGCCGCCCTGGCAGGCAATCCCGCCGCCACCGCCGCCGTGAAAACCGCCCTGAGCGGAAAT
CCCGTGCCCATTCTGATCCCCTGCCACCGGGTGGTGCAGGGCGACCTGGACGTGGGGGGCTACGAGGG
CGGGCTCGCCGTGAAAGAGTGGCTGCTGGCCCACGAGGGCCACAGACTGGGCAAGCCTGGGCTGGGTC
CAGCAGGCGGAGGCGCGCCAGGGTCTGGCGGCGGAAAACTTAGCGAGAGCGAGATCCGCTTCGGCCTG
AAAACCGAAGTGATGGGCCAGCACCTGATCTACCACGACGTGCTGAGCAGCACCCAGAAAACAGCCCA
CGAGCTGGCCAACAACAATGCCCCTGAGGGAACACTGGTGGTGGCCGATAAGCAGACAGCTGGCCGCG
GCGGAATGAGCCGCGTGTGGCACAGCCAAGAAGGCAACGGCGTCTGGATGAGCCTGATCCTGCGCCCT
GACATCCCTCTCCAGAAAACCCCTCAGCTGACACTGCTGGCTGCTGTGGCTGTGGTGCAGGGAATTGA
AGAAGCCGCCGGAATCCAGACCGACATCAAGTGGCCCAACGACATCCTGATCAACGGCAAGAAAACCG
TGGGCATCCTGACCGAGATGCAGGCCGAAGAGGACAGAGTCCGCAGCGTGATCATCGGCATCGGGATC
AATGTGAACCAGCAGCCTAACGACTTCCCCGACGAGCTGAAGGATATCGCCACAAGCCTGTCTCAGGC
CGCTGGCGAGAAGATTGATCGCGCTGGCGTGATCCAGCACATCCTGCTGTGCTTCGAGAAGCGGTACC
GCGACTACATGACCCACGGCTTCACCCCTATCAAACTGCTGTGGGAGAGCTACGCCCTCGGAATCGGC
ACCAACATGCGCGCCCGCACACTGAACGGCACCTTCTACGGCAAGGCCCTGGGAATTGATGACGAGGG
CGTTCTGCTGCTGGAAACAAACGAGGGCATCAAGAAAATCTACAGCGCCGACATCAGCCTGCGCCTCG
AGCACCACCACCACCACCAC

Appendix

Full images of Western blots, BG-FITC and CA-TMR stains. Orange boxes indicate the bands and lanes that are shown in the corresponding main or supporting figure. For BG-FITC stains lookup tables (LUTs) have been inverted and changed to linear green LUTs for the main and supporting figures. For CA-TMR stains LUTs have been inverted and changed to linear red LUTs for main figure 1e.
All antibodies have been tested individually before using them in combination.

Fig.1e

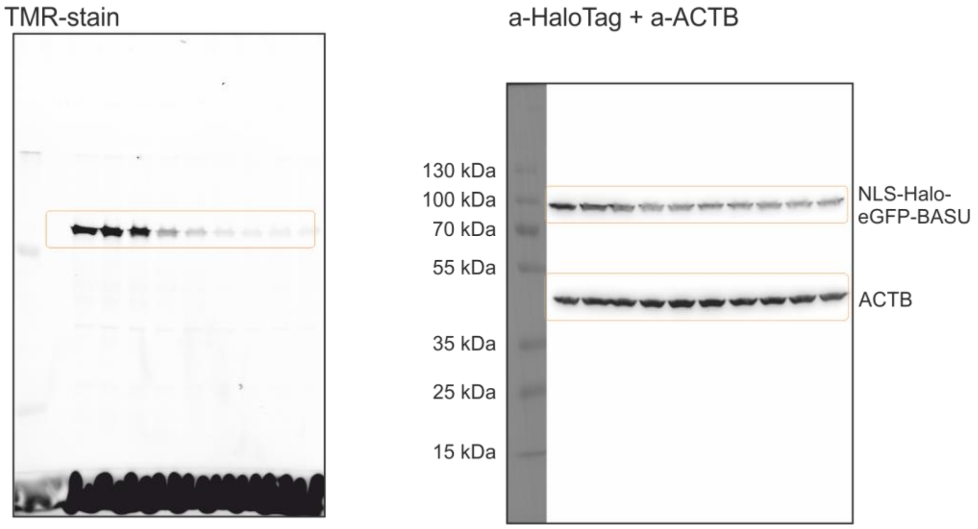

Fig. 2b

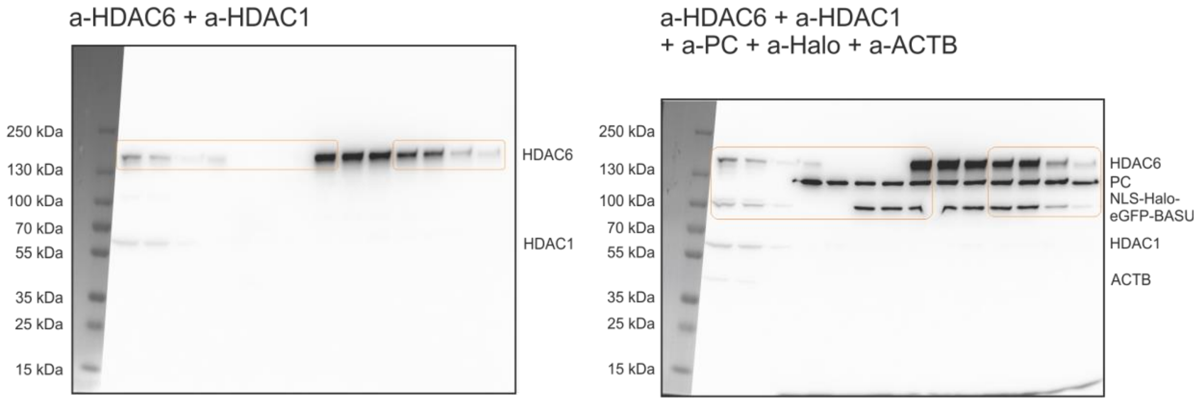

Fig. 3c

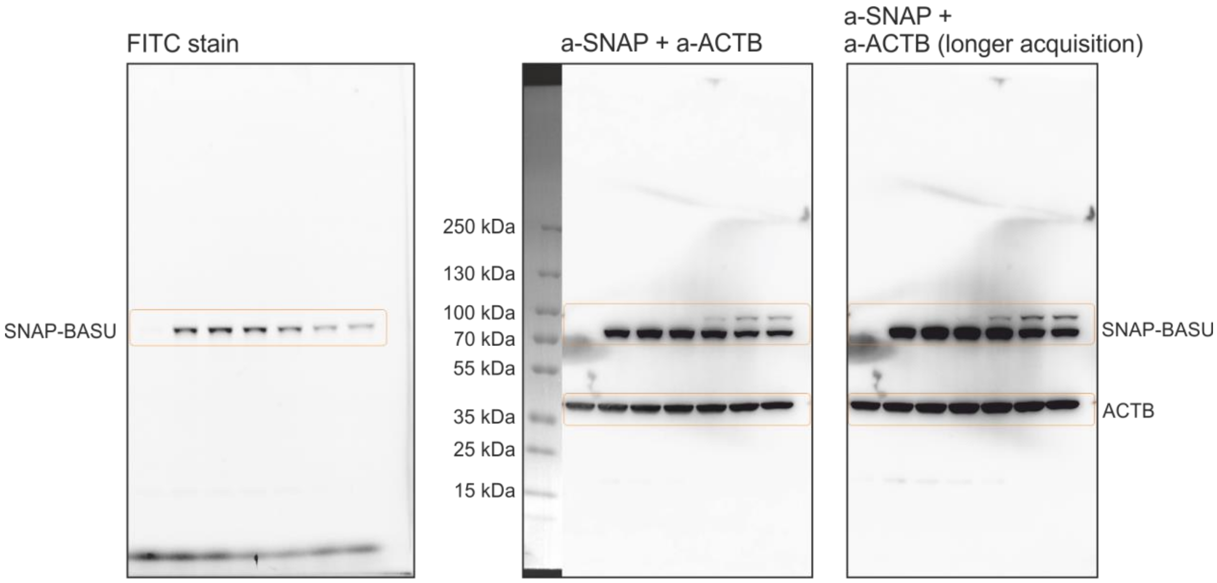

Extended Data Fig. 1i

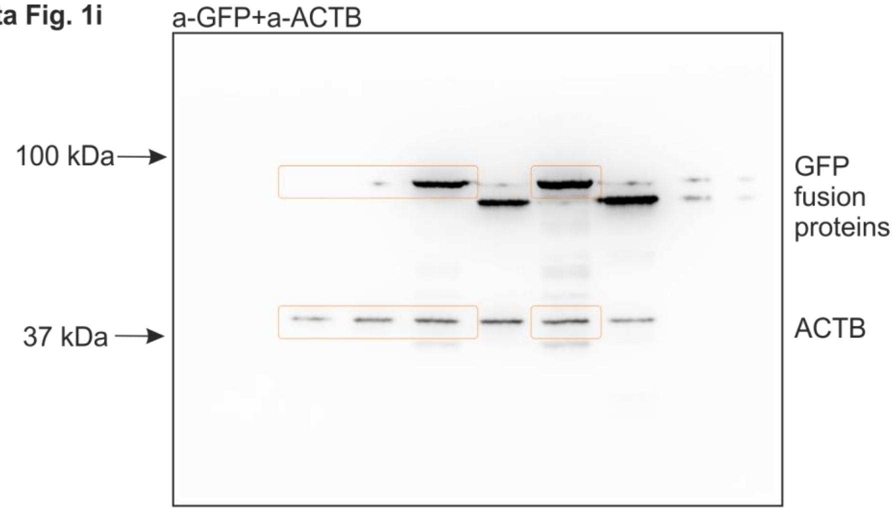

Extended Data Fig. 3a

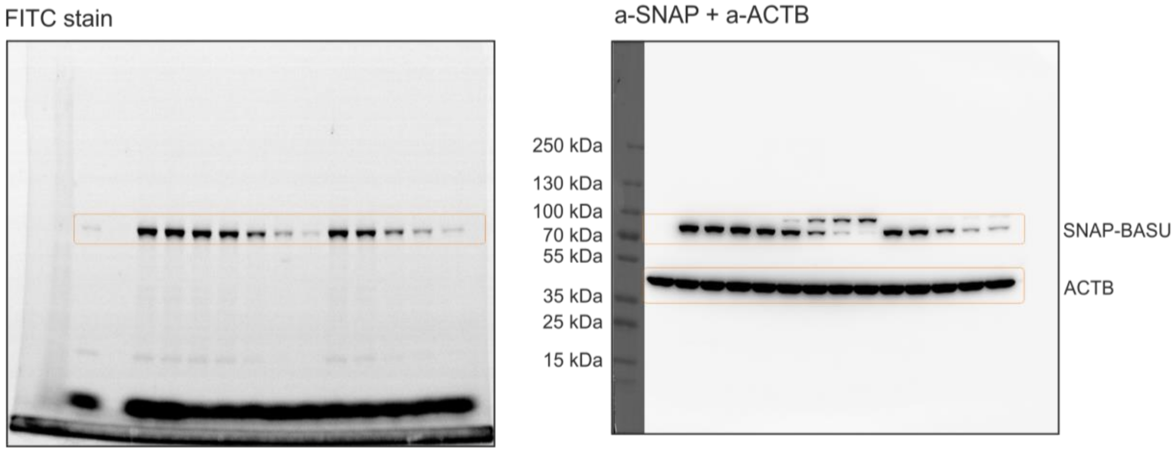

Supplementary Fig. 1a

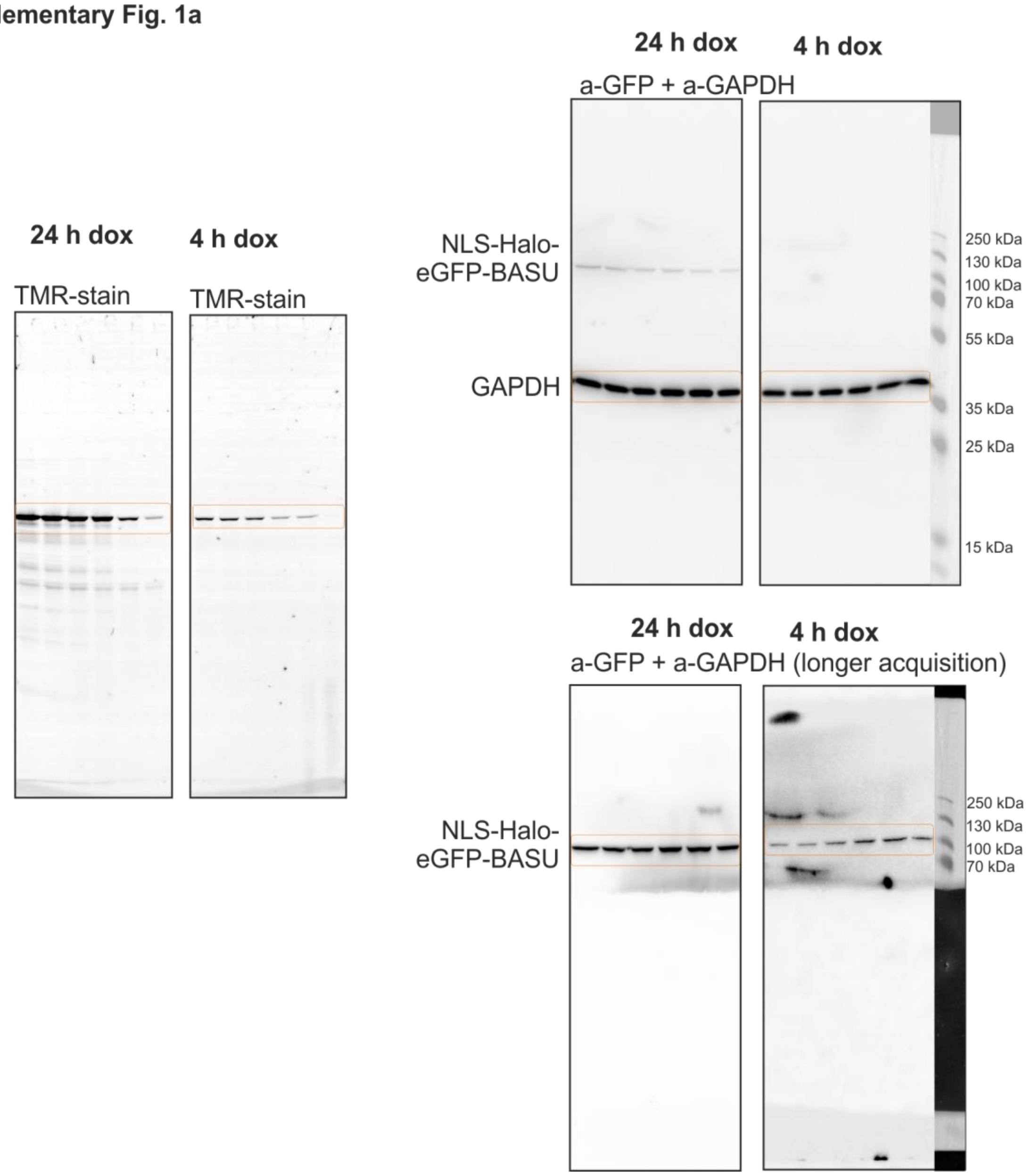

Supplementary Fig. 1b

a-HDAC6 + a-HDAC1

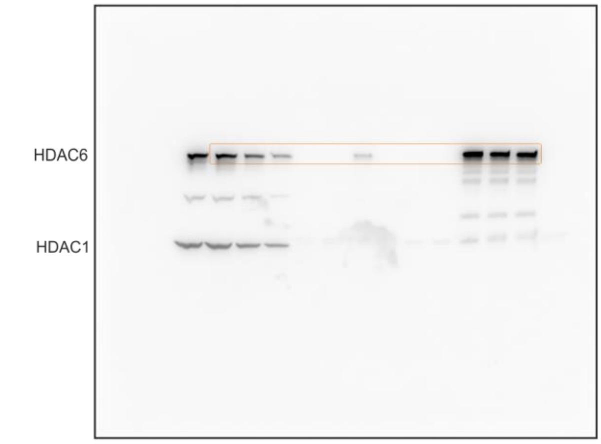

a-HDAC6 + a-HDAC1
+ a-PC + a-Halo + a-ACTB

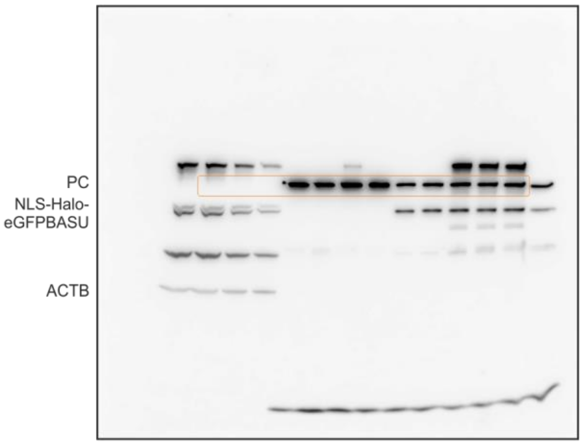

a-HDAC6 + a-HDAC1
+ a-PC + a-Halo + a-ACTB
+ a-KDM1

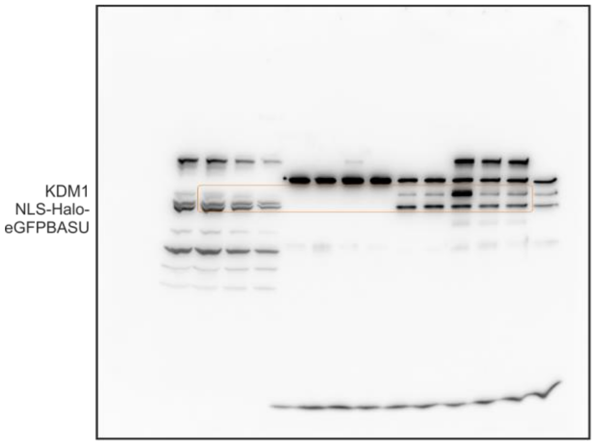

a-HDAC6 + a-HDAC1
+ a-PC + a-Halo + a-ACTB
+ a-KDM1 + a-HDAC3

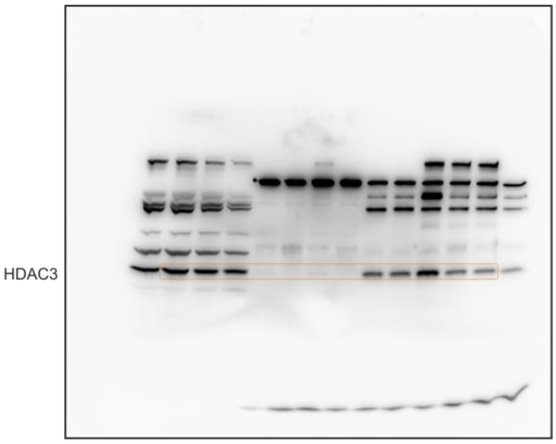

a-HDAC6 + a-HDAC1
+ a-PC + a-Halo + a-ACTB
+ a-KDM1 + a-HDAC3 + a-HDAC1 (prolonged incubation)

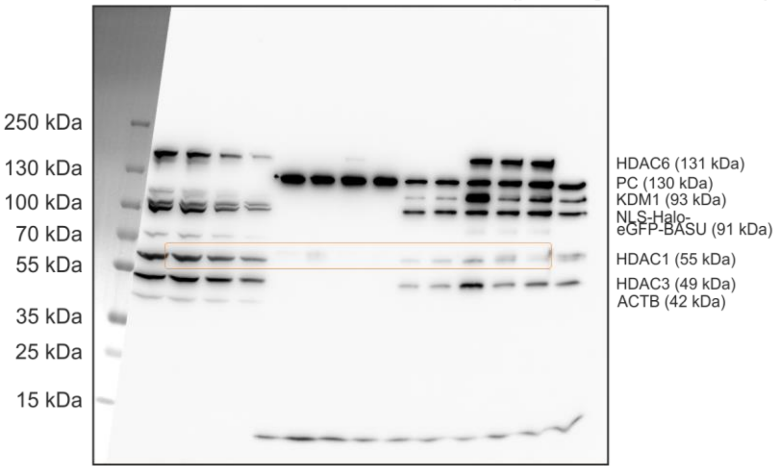

Supplementary Fig. 4b

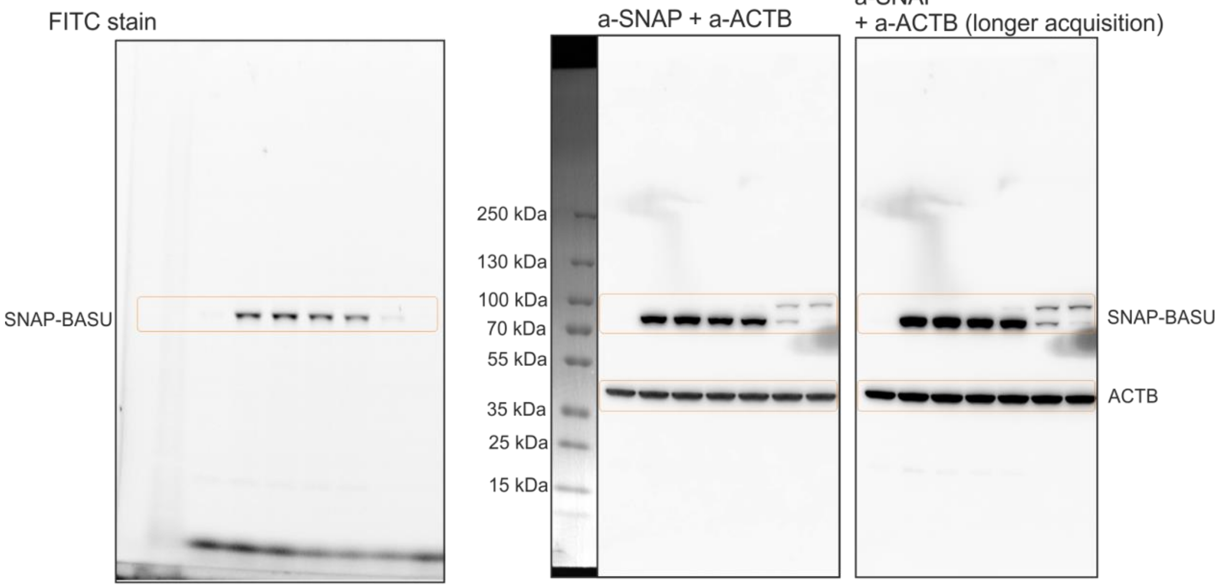

Supplement: Supplementary file 1 — Supplementary Figs. 1–15, Notes 1 and 2, Tables 1–3 and Appendix (uncropped western blot images). [file 41589_2023_1530_MOESM1_ESM.pdf]
