# Supplementary material for: Profiling the interactome of oligonucleotide drugs by proximity biotinylation
Source: Nat Chem Biol. 2024 Jan 17;20(5):555–65. doi: 10.1038/s41589-023-01530-z (PMC11062921; doi:10.1038/s41589-023-01530-z)
Supplement: Supplementary file 2 — Reporting Summary [file 41589_2023_1530_MOESM2_ESM.pdf]

nature portfolio

Corresponding author(s): Thorsten Stafforst

Last updated by author(s): 2023/10/13

# Reporting Summary

Nature Portfolio wishes to improve the reproducibility of the work that we publish. This form provides structure for consistency and transparency in reporting. For further information on Nature Portfolio policies, see our Editorial Policies and the Editorial Policy Checklist.

## Statistics

For all statistical analyses, confirm that the following items are present in the figure legend, table legend, main text, or Methods section.

| n/a | Confirmed | |
|---|---|---|
| ☐ | ☒ | The exact sample size (*n*) for each experimental group/condition, given as a discrete number and unit of measurement |
| ☐ | ☒ | A statement on whether measurements were taken from distinct samples or whether the same sample was measured repeatedly |
| ☐ | ☒ | The statistical test(s) used AND whether they are one- or two-sided<br>*Only common tests should be described solely by name; describe more complex techniques in the Methods section.* |
| ☒ | ☐ | A description of all covariates tested |
| ☒ | ☐ | A description of any assumptions or corrections, such as tests of normality and adjustment for multiple comparisons |
| ☐ | ☒ | A full description of the statistical parameters including central tendency (e.g. means) or other basic estimates (e.g. regression coefficient) AND variation (e.g. standard deviation) or associated estimates of uncertainty (e.g. confidence intervals) |
| ☐ | ☒ | For null hypothesis testing, the test statistic (e.g. *F*, *t*, *r*) with confidence intervals, effect sizes, degrees of freedom and *P* value noted<br>*Give P values as exact values whenever suitable.* |
| ☒ | ☐ | For Bayesian analysis, information on the choice of priors and Markov chain Monte Carlo settings |
| ☒ | ☐ | For hierarchical and complex designs, identification of the appropriate level for tests and full reporting of outcomes |
| ☒ | ☐ | Estimates of effect sizes (e.g. Cohen's *d*, Pearson's *r*), indicating how they were calculated |

*Our web collection on statistics for biologists contains articles on many of the points above.*

## Software and code

Policy information about availability of computer code

| | |
|---|---|
| Data collection | The following software was used for data collection:<br>Vilber FusionCapt Advance Solo 4 (Western blot detection with Fusion FX by Vilber)<br>Fuji Image Reader FLA-5000 Series V1.0 (TMR/FITC staining detection with FLA 5100 by Fujifilm)<br>Nikon NIS-Elements AR v5.02 (acquisition of microscopy images)<br>Life Technologies 7500 Software v2.3 (qPCR)<br>Tecan SparkControl v2.1 (CellTiter-Glo2.0 assay) |
| Data analysis | Nikon NIS Offfline Deconvolution 4.51 (deconvolution of microscopy images)<br>Image J win64 (adjustment of brightness and contrast of Western blots, cropping of Western blots, performing maximum projection of deconvoluted microscopy images and assigning LUTs)<br>MaxQuant software package version 1.5.2.8 (processing of SILAC-MS/MS data for Drug-ID experiments)<br>MaxQuant software package version 1.6.14.0 (processing of SILAC-MS/MS data for isASO-ID experiments)<br>Perseus software v1.6.1.3 (statistical analysis of SILAC-MS/MS data, preparation of scatterplots for Drug-ID experiments)<br>Perseus software v1.6.15.0 (preparation of scatterplots for isASO-ID experiments)<br>Life Technologies 7500 Software v2.3 (determining Ct values, qPCR)<br>Microsoft Excel 2016 (processing of qPCR and cell viablity data, enrichment tables and conditional formatting)<br>GraphPad Prism 8 (bar graphs)<br>CorelDraw 2017 (preparation of figures, coloring of scatterplots) |

For manuscripts utilizing custom algorithms or software that are central to the research but not yet described in published literature, software must be made available to editors and reviewers. We strongly encourage code deposition in a community repository (e.g. GitHub). See the Nature Portfolio guidelines for submitting code & software for further information.

# Data

Policy information about availability of data

All manuscripts must include a data availability statement. This statement should provide the following information, where applicable:

- Accession codes, unique identifiers, or web links for publicly available datasets
- A description of any restrictions on data availability
- For clinical datasets or third party data, please ensure that the statement adheres to our policy

The mass spectrometry proteomics data have been deposited to the ProteomeXchange Con-sortium via the PRIDE partner repository with the dataset identifier PXD045992. Further Supplementary Information on chemical synthesis, cloned constructs, and additional data is available in the online version of the paper.

# Human research participants

Policy information about studies involving human research participants and Sex and Gender in Research.

| | |
|---|---|
| Reporting on sex and gender | not applicable |
| Population characteristics | not applicable |
| Recruitment | not applicable |
| Ethics oversight | not applicable |

Note that full information on the approval of the study protocol must also be provided in the manuscript.

# Field-specific reporting

Please select the one below that is the best fit for your research. If you are not sure, read the appropriate sections before making your selection.

☒ Life sciences ☐ Behavioural & social sciences ☐ Ecological, evolutionary & environmental sciences

For a reference copy of the document with all sections, see nature.com/documents/nr-reporting-summary-flat.pdf

# Life sciences study design

All studies must disclose on these points even when the disclosure is negative.

| | |
|---|---|
| Sample size | SILAC-MS/MS experiments were performed in duplicates with swapped SILAC labels. All protein groups identified and enrichment in both replicates can be found in the provided primary data.<br>Microscopy images show a field of view that is representative for several biological replicates. For every condition several fields of views were acquired.<br>qPCR and cell viability measurements were performed in biological duplicates or triplicates. For each experiment technical triplicates were measured. Data points of biological replicates are always displayed individually. |
| Data exclusions | no data was excluded. |
| Replication | all experiments could reliably be reproduced and/or were in strong accordance with literature. As usual in the field of proteomics and due to complexity of SILAC experiments, two replicates were performed and correlation was checked. |
| Randomization | no randomization was performed, samples were treated identically side-by-side with the respective controls. |
| Blinding | Blinding was performed during the downstream analysis of SILAC-MS/MS experiments (sample preparation and measurement). At the other steps, no blinding was performed since samples that are directly compared with each other were typically prepared side-by-side using the same reagents. For samples that require different treatment (e.g. time course experiments), blinding cannot be performed at these stages. |

# Reporting for specific materials, systems and methods

We require information from authors about some types of materials, experimental systems and methods used in many studies. Here, indicate whether each material, system or method listed is relevant to your study. If you are not sure if a list item applies to your research, read the appropriate section before selecting a response.

## Materials & experimental systems

| n/a | Involved in the study | |
|---|---|---|
| ☐ | ☒ | Antibodies |
| ☐ | ☒ | Eukaryotic cell lines |
| ☒ | ☐ | Palaeontology and archaeology |
| ☒ | ☐ | Animals and other organisms |
| ☒ | ☐ | Clinical data |
| ☒ | ☐ | Dual use research of concern |

## Methods

| n/a | Involved in the study | |
|---|---|---|
| ☒ | ☐ | ChIP-seq |
| ☒ | ☐ | Flow cytometry |
| ☒ | ☐ | MRI-based neuroimaging |

# Antibodies

Antibodies used

The following antibodies have been used in this study (for more detailed information see Supplementary Table 3):
Rabbit α-SNAP (New England Bioloabs, P9310S)
Rabbit α-Halo (Promega, G9281)
Rabbit α-eGFP ( Sigma Aldrich, g1544
Rabbit α-PC (Sigma Aldrich, HPA043922 )
Mouse α-ACTB (Sigma Aldrich,A5441)
Mouse α-GAPDH (Thermo Scientific, GA1R)
Rabbit α-HDAC6 (Santa Cruz Biotechnology, H-300, sc-11420)
Mouse α-HDAC1 (Santa Cruz Biotechnology, sc-81698)
Rabbit α-HDAC3 (Santa Cruz Biotechnology H-99, sc-11417)
Rabbit α-KDM1 (Wolfram Antonin laboratory, FMI, Tuebingen, Germany)
Mouse α-NONO/p54/nrb (Santa Cruz Biotechnology sc-376865)
Goat a-mouse, HRP conjugate (Jackson Immuno Research, 115-035-003)
Goat a-mouse, HRP conjugate (Jackson Immuno Research, 111-035-003)
α-mouse AlexaFluor594 (Thermo Scientific A-11005)

Validation

Rabbit α-SNAP (New England Bioloabs, P9310S), validated in our laboratory via Western Blot of overexpressed SNAP-tag fusion proteins
Rabbit α-Halo (Promega, G9281), validated in our laboratory via Western Blot of overexpressed Halo-tag fusion proteins
Rabbit α-eGFP (Sigma Aldrich, g1544), validated in our laboratory via Western Blot of overexpressed of GFP-tag fusion proteins
Rabbit α-PC (Sigma Aldrich, HPA043922 ), validated in our laboratory by Western Blot after streptavidin enrichment of endogenous PC, also see PMID: 26302408
Rabbit α-HDAC6 (Santa Cruz Biotechnology, H-300, sc-11420) see PMID: 31270913
Mouse α-HDAC1 (Santa Cruz Biotechnology, sc-81698) see PMID: 31270913
Rabbit α-HDAC3 (Santa Cruz Biotechnology H-99, sc-11417) see PMID: 31270913
Rabbit α-KDM1 (Wolfram Antonin laboratory, FMI, Tuebingen, Germany) see PMID: 26224877
Mouse α-NONO/p54/nrb (Santa Cruz Biotechnology sc-376865) see PMID: 29165591
Mouse α-ACTB (Sigma Aldrich,A5441) see PMID: 30814728
Mouse α-GAPDH (Thermo Scientific, GA1R) see PMID: 31519936

# Eukaryotic cell lines

Policy information about cell lines and Sex and Gender in Research

Cell line source(s)

We generated cell lines derived from the parental Flp-In T-REx cell line (Catalog no. R78007, Thermo Fisher scientific):
- FlpIn Trex 293 NLS-eGFP-Halo-BASU
- FlpIn Trex 293 NES-eGFP-Halo-BASU
and HeLa cells (DSMZ, Braunschweig, Germany, no: ACC 57):
- Hela(BS)-Xlone-Puro_SNAPf-EGFP-BASU
- Hela(BS)-Xlone-Puro_NLS-SNAPf-EGFP-BASU

Authentication

Cell lines were obtained from commercial supplier. Cell lines were not additionally authenticated by us.

Mycoplasma contamination

HeLa and 293 FlpIn T-REx cells have been tested as mycoplasma-free in house.

Commonly misidentified lines
(See ICLAC register)

None.
