# Supplementary figures and images for: Profiling the interactome of oligonucleotide drugs by proximity biotinylation
Source: Nat Chem Biol. 2024 Jan 17;20(5):555–65. doi: 10.1038/s41589-023-01530-z (PMC11062921; doi:10.1038/s41589-023-01530-z)

**Fig. 2b**

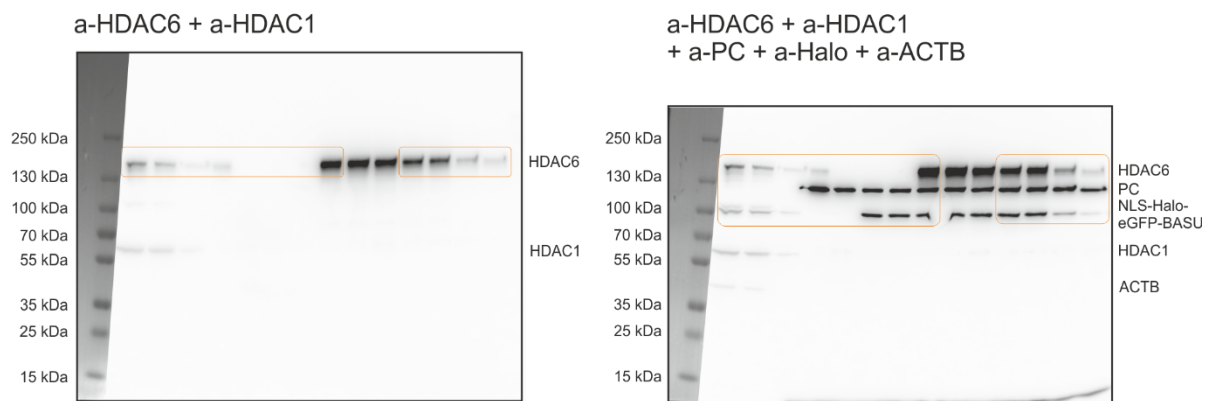

Supplement: Supplementary file 4 — Unprocessed western blot for Fig. 2b. [file 41589_2023_1530_MOESM4_ESM.pdf]
