# Supplementary material for: Profiling the interactome of oligonucleotide drugs by proximity biotinylation
Source: Nat Chem Biol. 2024 Jan 17;20(5):555–65. doi: 10.1038/s41589-023-01530-z (PMC11062921; doi:10.1038/s41589-023-01530-z)
Supplement: Supplementary file 5 — Unprocessed western blot for Fig. 3c. [file 41589_2023_1530_MOESM5_ESM.pdf]

Fig. 3c

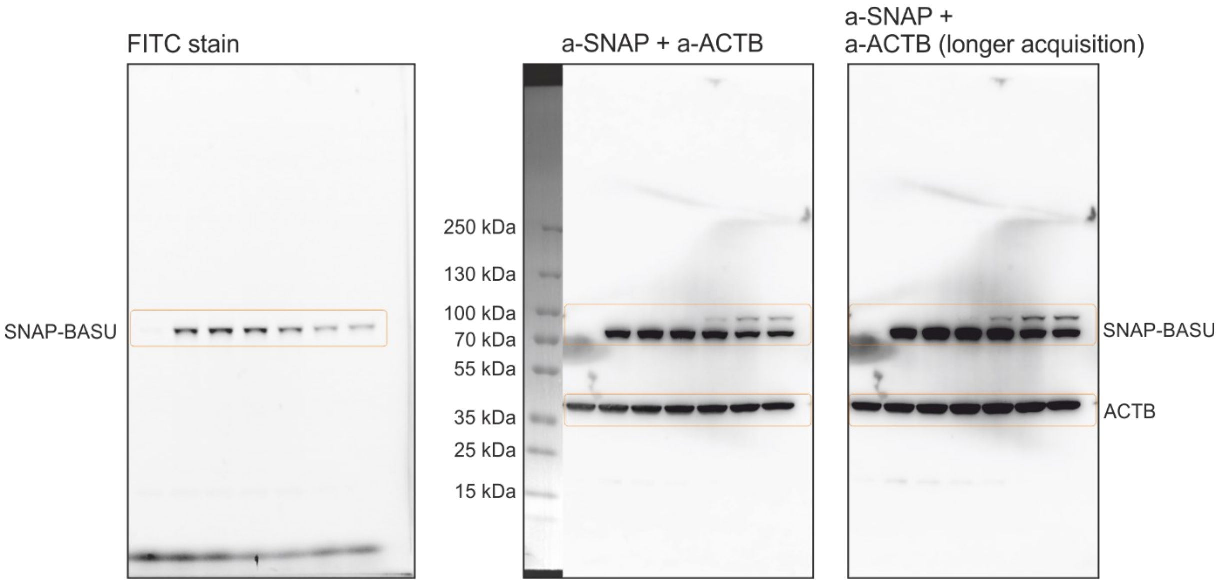

Orange boxes indicate the bands and lanes that are shown in the corresponding main figure. For BG-FITC stains lookup tables (LUTs) have been inverted and changed to linear green LUTs in the main figure.
